# Poorly perfused tumor regions harbor T cells with a glucose-dependent effector phenotype

**DOI:** 10.1038/s44319-026-00799-0

**Published:** 2026-05-27

**Authors:** Marta Riera-Borrull, Víctor Cerdán Porqueras, Sonia Tejedor Vaquero, Claudia Tomaselli González, Mauricio Guzmán, Donata Martinuzzi, Diego Ceacero-Heras, Andrea Cerutti, Jose Aramburu, Cristina López-Rodríguez

**Affiliations:** 1 Department of Medicine and Life Sciences, Universitat Pompeu Fabra, Barcelona, Spain; 2 Translational Clinical Research Program, Hospital del Mar Research Institute (HMRI), Barcelona, Spain; 3 Catalan Institute for Research and Advanced Studies (ICREA), Barcelona, Spain; 4 Present Address: Orikine Bio, Barcelona, Spain

**Keywords:** Cancer, Immunology, Metabolism

## Abstract

Effector T lymphocytes are avid nutrient consumers, but can function in nutrient-poor tumor microenvironments. Availability of key nutrients such as glucose inside the tumor is not homogeneous, and how tumor-infiltrating T lymphocytes (TILs) differ between regions with better and poorer blood perfusion is not well known. Here we show that in vitro-stimulated TILs can induce substantial production of hallmark glucose-dependent cytokines under glucose concentrations 20 times lower than in blood. In vivo, effector TILs in tumor regions with poor access to blood show comparable capacity for inducing IFNγ and granzyme B to TILs with fuller accessibility; exhibit an enhanced type I IFN response supported by local myeloid cells; and unexpectedly, have reduced expression of immune checkpoint and Treg-associated markers. TILs with poor blood accessibility also have lower biosynthetic activity than highly blood-accessible TILs, yet both compartments depend fundamentally on glucose for ATP production. Thus, effector T lymphocytes in poorly perfused tumor regions can maintain specific glucose-dependent responses, and might be partially protected from inhibitory and exhausting pressure from the tumor microenvironment.

## Synopsis

Compared to intratumor T cells (TILs) closer to blood vessels, distant TILs have reduced biosynthetic activity, an enhanced IFN-I response supported by local myeloid cells, and express lower levels of immune checkpoint and Treg-associated markers.

- Distant TILs show reduced biosynthetic activity as TILs closer to blood vessels, but both depend on glucose for ATP production.
- TILs with reduced access to blood are competent to induced Th1-like responses upon ex vivo stimulation.
- TILs in poorly perfused tumor regions exhibit an enhanced type I IFN response reminiscent of bystander resident memory cells.
- Distant TILs show reduced expression of immune checkpoint and Treg-associated markers.

## Abstract (web summary)

Compared to intratumor T cells (TILs) closer to blood vessels, distant TILs have reduced biosynthetic activity, an enhanced IFN-I response supported by local myeloid cells, and express lower levels of immune checkpoint and Treg-associated markers.

## Graphical abstract

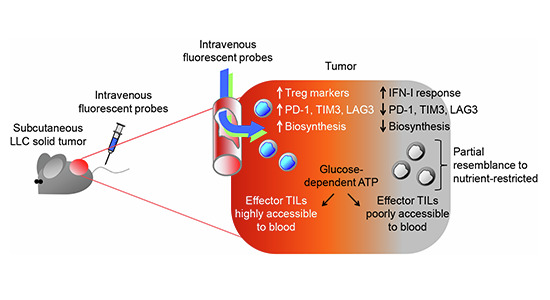

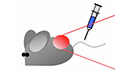

## Introduction

Activation of effector T lymphocytes is energetically demanding and requires a sufficient supply of specific nutrients. For instance, activation of naive T lymphocytes to effector T helper (Th) 1 and Th17 cells, considered the most nutrient- and energy-avid versions of Th cells, requires abundant glucose and glutamine (Macintyre et al, 2014; Michalek et al, 2011; Shi et al, 2011). Accelerated glucose consumption by aerobic glycolysis is also characteristic of the reactivation of memory to effector T cells upon T cell receptor restimulation (Menk et al, 2018; Gubser et al, 2013). By contrast, increased glucose processing through the Krebs cycle, and a lipid oxidation-dependent metabolism are more characteristic of regulatory T (Treg) cells (Beier et al, 2015; Macintyre et al, 2014; Michalek et al, 2011; de Kivit et al, 2020; de Candia et al, 2022). Glucose is a central and versatile nutrient for activated T lymphocytes, as it fuels rapid ATP production through glycolysis, serves as a basic building block for amino acid biosynthesis, and feeds the pentose phosphate pathway (PPP) (Lunt and Vander Heiden, 2011). The PPP provides nucleotide precursors necessary to sustain T lymphocyte growth and proliferati.on, and is also a main source of NADPH, a major reducing agent in biosynthetic reactions and for regenerating the pool of reduced glutathione (Lunt and Vander Heiden, 2011; Ma et al, 2018). The relevance of glucose for effector T lymphocytes is illustrated by the suppression of T cell activation by the glycolysis inhibitor 2-deoxyglucose (2DG) (Macintyre et al, 2014; Shi et al, 2011), and by work showing that T lymphocytes lacking the glucose transporter Glut1 fail to activate properly as effector cells and are biased toward Treg (Macintyre et al, 2014).

These observations connect with the problem that effector T lymphocytes encounter in metabolically hostile niches, such as solid tumors and infected tissues, where oxygen and glucose supply is limited (Semenza, 2013; Ackerman and Simon, 2014; Hirayama et al, 2009; Schroeder et al, 2005; Chang et al, 2013; Hurd et al, 1979; Pettersson et al, 1982; Valdés et al, 2010). In contrast with lymph nodes where T lymphocyte activation occurs under optimal nutrient availability, the tumor microenvironment (TME) is one of considerable competition for nutrients between multiple cell types. This is even more critical in poorly irrigated regions, where T lymphocytes might not be able to obtain enough nutrients to sustain antitumor function. The metabolite landscape in the TME can be quite complex, with not only limited glucose availability and low oxygen levels, but also elevated levels of lactate and acetate with respect to blood or lymph nodes (Schroeder et al, 2005; Zappasodi et al, 2021; Qiu et al, 2019; Watson et al, 2021; Chapman and Chi, 2022). In this regard, the low glucose and high lactate levels characteristic of highly glycolytic tumors can promote tumor-tolerant Treg cells instead of antitumor effector T lymphocytes (Watson et al, 2021; Zappasodi et al, 2021). On the other hand, a glucose-poor TME might not necessarily prevent TIL responsiveness to stimulation, as seen in the activation of bulk CD4 and CD8 TILs, as well as tumor-infiltrating Treg cells, by anti-checkpoint antibodies (Zappasodi et al, 2021; Kumagai et al, 2022; Reinfeld et al, 2021). In addition, TILs can resort to locally abundant metabolites in tumors, as shown for effector CD8 T lymphocytes metabolizing acetate to sustain interferon gamma (IFNγ) production and antitumor activity under reduced glucose availability (Qiu et al, 2019). Altogether, these observations raise the question of which functional features can be expected from TILs in tumor regions with abundant or limited access to blood-transported nutrients, where they face different metabolic conditions. Identifying these characteristics can help understand the heterogeneous landscape of T lymphocyte responses to tumor cells.

At present, it is not known to what extent T cells in tumor regions with poor access to blood are impaired or dysfunctional, and which characteristics distinguish them from effector T cells in well perfused/irrigated tumor areas. Here we have addressed these questions by characterizing effector CD4 and CD8 T lymphocytes isolated by their uptake of probes 2-NBDG and Hoechst 33342 intravenously delivered to the tumor, as a proxy for blood accessibility and exposure to different metabolic conditions. We have compared these cells with TILs activated ex vivo in well-defined glucose-restricted conditions, and with tumor-inexperienced CD4 T cells from lymph nodes. Our results reveal that both effector TILs and tumor-inexperienced T lymphocytes can achieve substantial activation and maintain energy sufficiency under very low levels of glucose. We also identify distinct gene signatures of effector TILs in poorly perfused tumor regions in vivo, and unexpectedly find that they have reduced expression of immune checkpoint, exhaustion and Treg-associated markers.

## Results

### CD4 T lymphocytes can express characteristic Th1 and Th17 glucose-dependent cytokines when activated under very low glucose levels

**Figure 1:**
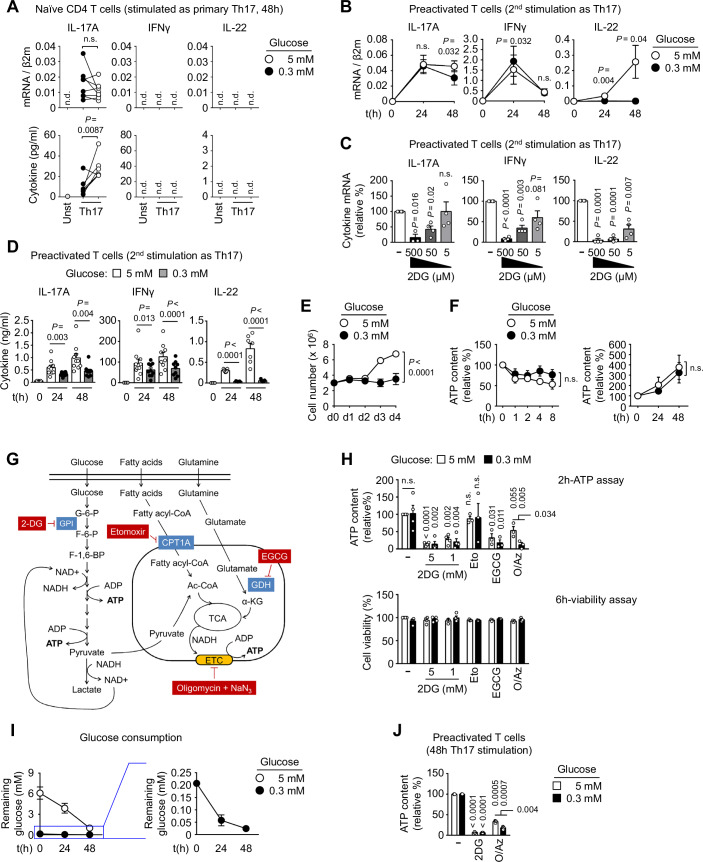
CD4 T lymphocytes can express glucose-dependent hallmark Th1 and Th17 cytokines when activated under low glucose levels. (**A**) Upper panels show the mRNA expression of the indicated cytokines in FACS-sorted naive CD4 T lymphocytes stimulated with anti-CD3 and anti-CD28 antibodies plus IL-2, TGFβ and IL-6 (primary Th17) in medium with normal (5 mM) or low (0.3 mM) glucose. Unstimulated controls were kept in medium with 5 mM glucose and IL-2, but without anti-CD3 and anti-CD28 antibodies nor TGFβ and IL-6. n.d. not detected. Bottom panels show IL-17A concentration in the culture supernatants, detected with Legendplex bead arrays. Results are from 7-8 biological replicates (different mice) in one experiment. Dots are connected by lines to better visualize differences between 5 and 0.3 mM glucose in each individual sample. (**B**) Cytokine mRNA expression in CD4 cells (from spleen and lymph nodes) that had been preactivated for 5 days in non-polarizing (Th0) conditions with anti-CD3 and anti-CD28 plus IL-2 and then restimulated as Th17 (2nd Th17) with anti-CD3 and anti-CD28 plus TGFβ and IL-6. Results show the mean ± SEM from ten biological replicates. (**C**) Inhibition of cytokine mRNA expression by the non-metabolizable glucose analog 2-deoxyglucose (2DG) in preactivated CD4 T cells restimulated as Th17 cells as in (**B**). Results show the mean ± SEM from four biological replicates. Cytokine mRNA expression was first normalized to β2m mRNA level in the same sample, and then referred to the control without 2DG (which was given a value of 100%). (**D**) Cytokine production in preactivated Th17 cells, as in (**B**), was measured by ELISA. Results show the mean ± SEM from 7 to 10 biological replicates. (**E**) Cell number of T lymphocytes preactivated as Th0 in 5 mM glucose and then restimulated as Th17, as in (**B**), for up to 4 days in 5 or 0.3 mM glucose. Results show the mean ± SEM from 2 to 4 biological replicates. (**F**) Intracellular ATP content in CD4 cells activated as in (**B**). Results are shown relative to the time of Th17 restimulation (*t *= 0). Results show the mean ± SEM from three to five biological replicates. (**G**) Diagram of cellular pathways producing ATP and points of pharmacological inhibition. (**H**) Intracellular ATP content in preactivated CD4 cells restimulated as Th17 as in (**B**) for 24 h and then treated with the indicated metabolic inhibitors for 2 h (upper panel). 2-deoxyglucose (2DG, 1 and 5 mM), epigallocatechin gallate (EGCG, 50 µM), etomoxir (Eto, 200 µM), and oligomycin plus sodium azide (0.1 µg/ml and 20 mM, respectively) (O/Az). Cell viability of parallel samples cultured for 6 h with the inhibitors is also shown (bottom panel). Results show the mean ± SEM from three to four biological replicates. *P* values for the comparisons between each condition and the 5 mM glucose control are indicated (vertical values). (**I**) Glucose remaining in the supernatant of cultures of preactivated CD4 cells restimulated as Th17 in 5 or 0.3 mM glucose medium up to 48 h as in (**B**). Results show the mean ± SEM from three to four biological replicates. (**J**) Contribution of glucose and mitochondrial respiration to intracellular ATP levels in preactivated CD4 cells after 48 h of Th17 restimulation in 5 or 0.3 mM glucose as in (**B**). Inhibitors 2-deoxyglucose (2DG, 5 mM) and oligomycin (0.1 µg/ml) plus sodium azide (20 mM) were added in the last 2 h of culture. Results show the mean ± SEM from three biological replicates. *P* values for the comparisons between each condition and its respective 5 mM or 0.3 mM glucose control (100%) are indicated (vertical values). Statistical significance was assessed by a paired *t* test (**A**, **B**, **D**), a two-way ANOVA test (**E**, **F**) or a one-sample *t* test for comparison with the reference sample (100%) in (**C**, **H**, **J**). Significant *P* values (<0.05) and *P* values between 0.05 and 0.1 are indicated. n.s. not significant. Source data are available online for this figure.

**Figure EV1:**
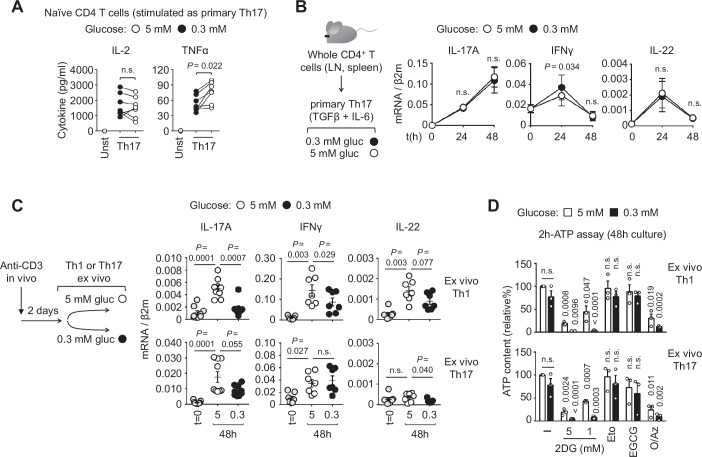
Glucose restriction has a variable impact on Th1 and Th17 cytokine expression in CD4 cells activated under different conditions. (**A**) Production of the indicated cytokines in naive CD4 T lymphocytes stimulated with anti-CD3 and anti-CD28 antibodies plus IL-2, TGFβ and IL-6 (primary Th17) in medium with normal (5 mM) or low (0.3 mM) glucose. Samples are the same as in Fig. 1A. Results show the mean ± SEM from seven biological replicates (different mice) in one experiment. Dots are connected by lines to better visualize differences between 5 and 0.3 mM glucose in each individual sample. Statistical significance was assessed with a paired *t* test. (**B**) Expression of the indicated cytokines in CD4 T lymphocytes freshly isolated from a mixture of lymph nodes and spleen, and stimulated with anti-CD3 and anti-CD28 antibodies plus TGFβ and IL-6 (primary Th17). Results show the mean ± SEM from two experiments, with two to three biological replicates (different mice) each. Statistical significance was assessed with a paired *t* test. (**C**) Cytokine mRNA expression in lymph node CD4 T cells preactivated in vivo with injected anti-CD3 antibody and isolated from the mice 2 days later to be restimulated in culture as Th1 (with IL-12) or Th17 (TGFβ and IL-6) for 48 h. Results show the mean ± SEM from seven to nine biological replicates. Statistical significance was assessed by a paired *t* test to compare the two conditions (0.3 and 5 mM glucose) for each sample. (**D**) Intracellular ATP content in Th1 or Th17 cells stimulated as in (**C**) for 48 h and then treated with the indicated metabolic inhibitors for the last 2 h. 2-deoxyglucose (2DG, 1 and 5 mM), epigallocatechin gallate (EGCG, 50 µM), etomoxir (Eto, 200 µM), and oligomycin plus sodium azide (0.1 µg/ml and 20 mM, respectively) (O/Az). Results show the mean ± SEM from three biological replicates. Statistical significance was assessed with a one-sample *t* test for comparison with the reference 5 mM glucose control sample without inhibitors. *P* values for the comparisons between each condition and the control are indicated with vertical values. Significant *P* values (<0.05) and *P* values between 0.05 and 0.1 are indicated. n.s. not significant. Source data are available online for this figure.

We analyzed how glucose limitation affected CD4 T cell activation by comparing 5 mM glucose, a normal concentration in blood and lymphoid tissues, with 0.3 mM glucose, in the range (0.1–1 mM) measured in tumors and inflamed tissues in various pathological conditions (Hirayama et al, 2009; Chang et al, 2013; Ho et al, 2015) (Fig. 1A). For these experiments we initially chose Th17 stimulatory conditions because they induce activated T cells that are markedly glycolytic and avid glucose consumers (Michalek et al, 2011). First, we tested naive CD4 T cells, FACS-sorted from lymph nodes and spleen as CD44 ^neg^ CD62L ^+^, and stimulated them in Th17 conditions for 48 h. While primary Th17 cells induced IL-17A mRNA comparably in 0.3 mM or 5 mM glucose, stimulation in low glucose reduced the production of IL-17A protein and TNFα (Figs. 1A and EV1A). We did not detect IFNγ and IL-22 mRNA in these cultures, or significant accumulation of either cytokine in the culture supernatant (Fig. 1A). We obtained similar results with freshly isolated whole CD4 T cells, whose induction of IL-17A mRNA during primary Th17 stimulation was not impaired by glucose limitation (Fig. EV1B). Induction of IFNγ mRNA in these cultures was modest, and expression of IL-22 mRNA was very low, and both were comparable between low and normal glucose (Fig. EV1B). Next, we tested CD4 T cells that had been preactivated in vitro as Th0 and then restimulated as Th17. These cells induced IL-17A and IFNγ mRNA similarly in normal and low glucose, but induction of IL-22 was substantially reduced in low glucose (Fig. 1B). We confirmed that expression of these cytokines was indeed glucose-dependent as it was suppressed by the glycolysis inhibitor 2-deoxyglucose (2DG) (Fig. 1C). Production of IL-17A and IFNγ protein in preactivated T cells restimulated as Th17 was partially inhibited under low glucose, but they still secreted substantial levels of both cytokines up to 48 h; however, production of IL-22 was clearly impaired (Fig. 1D). We also observed that glucose restriction arrested cell proliferation, although cultures could maintain their cellularity in low glucose for at least 4 days (Fig. 1E).

### Very low glucose concentrations can sustain energy production in activated CD4 T lymphocytes

We next asked whether activation of CD4 T lymphocytes in low glucose caused ATP stress or a shift in ATP production from alternative fuels such as glutamine or fatty acids. We assessed this in preactivated T lymphocytes restimulated as Th17 as in Fig. 1B–D. Intracellular ATP content was comparable between cells cultured in 0.3 and 5 mM glucose up to 2 days (Fig. 1F). Contribution of main ATP production pathways was assessed in the last 2 h of culture by adding different metabolic inhibitors without replacing the medium. We used 2-deoxyglucose (2DG) to block glycolysis, epigallocatechin gallate (EGCG) to inhibit conversion of glutamate to α-ketoglutarate, etomoxir for blocking the mitochondrial uptake of long-chain fatty acids, and oligomycin plus sodium azide to suppress ATP production through mitochondrial respiration and the electron transport chain (ETC) (Fig. 1G,H). These inhibitors did not impair cell viability for at least 6 h (Fig. 1H, bottom panel). We observed that preactivated T cells restimulated as Th17 cells in 0.3 mM glucose obtained most of their ATP from glucose, but were more dependent on mitochondrial respiration than cells in 5 mM glucose (Fig. 1H, upper panel). Both were comparably dependent on glutamate to maintain their ATP levels, and did not depend on mitochondrial uptake of long-chain fatty acids (Fig. 1H, upper panel). We were surprised that CD4 cells activated in 0.3 mM glucose for 48 h might have enough glucose to produce ATP. Indeed, they ended up with less than 50 μM extracellular glucose by 48 h (Fig. 1I), and even then, they kept using glucose as a main nutrient for ATP production (Fig. 1J).

To find whether a similar dependence on glucose happened in other stimulatory conditions, we analyzed CD4 T cells primed in vivo with anti-CD3 (Esplugues et al, 2011; Alberdi et al, 2017), and reactivated ex vivo as Th1 or Th17. In this experiment, we did not sort effector T cells, and therefore the responses seen comprise the contribution of cells in different activation states. These cells showed a variable dependence on glucose for the expression of specific cytokine mRNAs (Fig. EV1C). Regarding ATP, T cells activated as either Th1 or Th17 in 0.3 mM or 5 mM glucose were both highly dependent on glycolysis and mitochondrial respiration, and largely independent of glutaminolysis and fatty acid oxidation (Fig. EV1D), in line with our previous results (Fig. 1H). Altogether, our results indicate that CD4 T lymphocytes with different activation histories might exhibit different sensitivity to glucose limitation for the expression of specific cytokines, but still relied on glucose as a main source of ATP, even under severely limited glucose availability.

### Delayed attenuation of mTORC1 activity under low glucose influences cytokine expression in activated CD4 T lymphocytes

**Figure 2:**
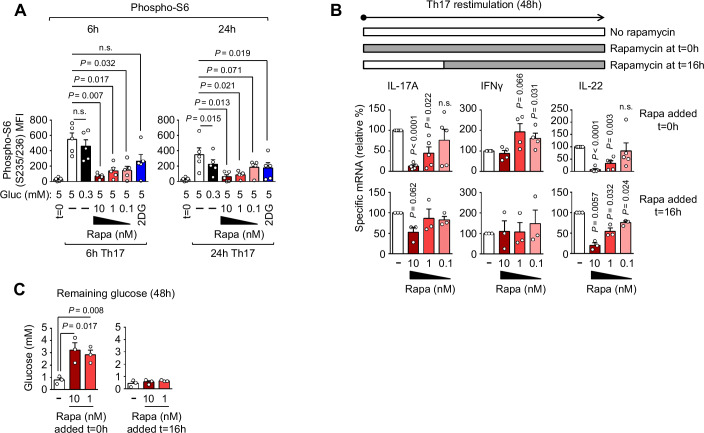
Activated CD4 T lymphocytes can maintain mTORC1 activity under very low glucose levels. (**A**) Intracellular levels of phosphorylated S6 in preactivated CD4 cells and then restimulated as Th17 for 6 or 24 h in 5 or 0.3 mM glucose and with the inhibitors 2DG (2 mM) and rapamycin (Rapa, 0.1 to 10 nM), as indicated. Phospho-S6 was analyzed by flow cytometry and its levels are shown as mean fluorescence intensity (MFI). Results show the mean ± SEM from five biological replicates. Statistical significance was assessed with a paired *t* test. (**B**) Cytokine mRNA expression in CD4 cells activated for 5 days in non-polarizing (Th0) conditions and then restimulated as Th17 in 5 mM glucose for 48 h, without or with rapamycin added at *t* = 0 h or *t* = 16 h (as in top diagram). Results are shown relative to the time of Th17 restimulation (*t* = 0 h). Results show the mean ± SEM from three to five biological replicates. Cytokine mRNA expression in was first normalized to β2m mRNA level in the same sample, and then referred to the control without 2DG (which was given a value of 100%). Statistical significance was assessed with a one-sample *t* test for comparison with the reference sample without rapamycin. *P* values for the comparisons between each condition and the 5 mM glucose control (100%) are indicated (vertical values). (**C**) Glucose remaining in supernatants of cultures of preactivated CD4 cells restimulated as Th17 in 5 mM glucose up to 48 h, without or with rapamycin as in (**B**). Results show the mean ± SEM from three biological replicates for each rapamycin time point. Statistical significance was assessed with an unpaired *t* test. Significant *P* values (<0.05) and *P* values between 0.05 and 0.1 are indicated. Source data are available online for this figure.

Glucose sufficiency is needed for the activity of the mammalian or mechanistic target of rapamycin (mTOR), a central sensor of nutrient and energy availability that plays a major role in T lymphocyte activation (Shi et al, 2011; Powell et al, 2012). We analyzed the effect of low glucose on mTORC1 by assessing the phosphorylation of the ribosomal protein S6, a target of the mTORC1-activated kinase S6K1 (Procaccini et al, 2010; Ray et al, 2015). We first confirmed that mTORC1 was glucose-sensitive in our assay, as it was inhibited by 2DG (Fig. 2A). Preactivated T lymphocytes restimulated as Th17 in low glucose exhibited a mild reduction of mTORC1 activity at 24 h, but not earlier at 6 h (Fig. 2A). The extent of mTORC1 inhibition under low glucose was comparable to that achieved by very low concentrations of rapamycin, between 0.1 and 1 nM (Fig. 2A). These results suggested that glucose limitation during T lymphocyte activation caused a slow and progressive attenuation of mTORC1. We then assessed the effect of mTORC1 inhibition on gene expression and glucose consumption, for which we treated cells with different concentrations of rapamycin added either just before stimulation or 16 h later to mimic a delayed inhibition of mTORC1 (Fig. 2B). Induction of IL-22 mRNA was the most mTORC1-dependent, requiring sustained mTORC1 activity; IL-17A mRNA was less mTORC1-dependent; and induction of IFNγ was mTORC1-independent, with its mRNA levels even increasing under sustained mTORC1 inhibition (Fig. 2B). Glucose consumption by these cells was also reduced by inhibiting mTORC1, but only when inhibition was done since the beginning of their stimulation (Fig. 2C). In sum, our results so far suggested that CD4 cells in a glucose-restricted environment are capable of maintaining an activated state for days, inducing diverse glucose-dependent cytokines, using glucose as a main fuel for ATP production, and sustaining mTORC1 activity.

### Identification of functionally distinct tumor-infiltrating T lymphocytes (TILs) in vivo by their uptake of the intravenously delivered deoxyglucose analog 2-NBDG

**Figure EV2:**
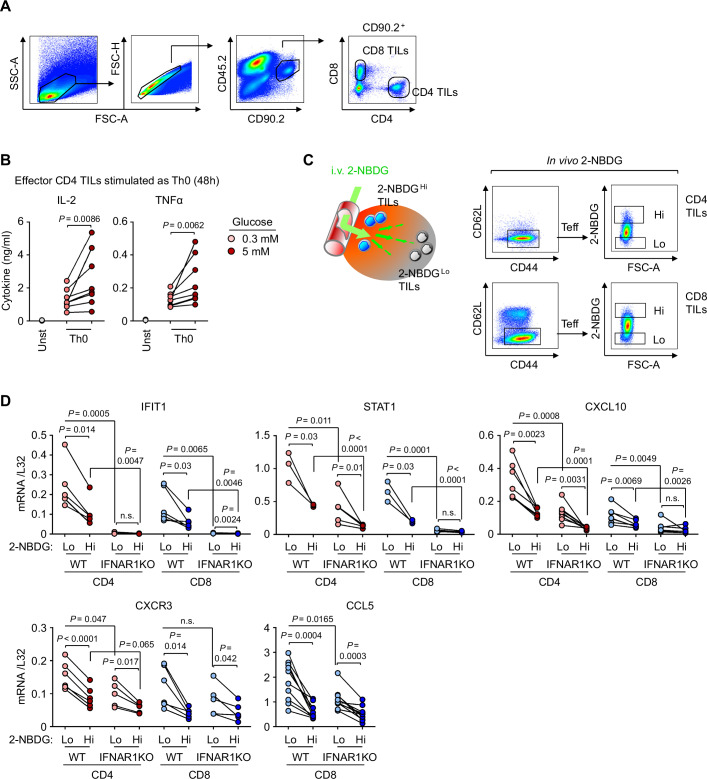
Gating strategies for isolating CD4 and CD8 T lymphocytes infiltrating LLC tumors, and cytokine production analysis in TILs activated ex vivo. (**A**) Flow cytometry gating strategies used to isolate CD4 and CD8 T lymphocytes infiltrating LLC tumors. (**B**) Production of IL-2 and TNFα by CD4 effector TILs freshly isolated by FACS sorting from LLC tumors and either left unstimulated or stimulated with anti-CD3 and anti-CD28 plus IL-2 (Th0 conditions) 48 h in medium with 5 mM or 0.3 mM glucose. Unstimulated controls were kept in medium with 5 mM glucose and IL-2 but without anti-CD3 and anti-CD28 antibodies. This experiment used TILs not traced with 2-NBDG nor Hoechst. Cytokine concentration was measured with Legendplex bead arrays. Results show 8 biological replicates (8 mice), and correspond to the samples shown in Fig. 3A. Statistical significance was assessed by a paired *t* test to compare the two conditions (0.3 and 5 mM glucose) for each sample. (**C**) Diagram illustrating the rationale of the experiment, and flow cytometry panels representative of effector memory (Teff) CD4 and CD8 TILs, and their uptake of intravenously injected 2-NBDG. (**D**) Expression of the indicated genes in effector CD4 and CD8 TILs sorted by their 2-NBDG uptake in vivo, in wild-type and IFNAR1-deficient mice (IFNAR1KO). Results are from three to ten biological replicates. Dots are connected by lines to better visualize differences between 2-NBDG ^Lo^ versus ^Hi^ cells within each individual tumor. Statistical significance was assessed with a paired *t* test to compare ^Lo^ and ^Hi^ cells within the same tumor, and with an unpaired *t* test to compare WT with IFNAR1KO. Significant *P* values (<0.05) and *P* values between 0.05 and 0.1 are indicated. n.s. not significant. Source data are available online for this figure.

**Figure 3:**
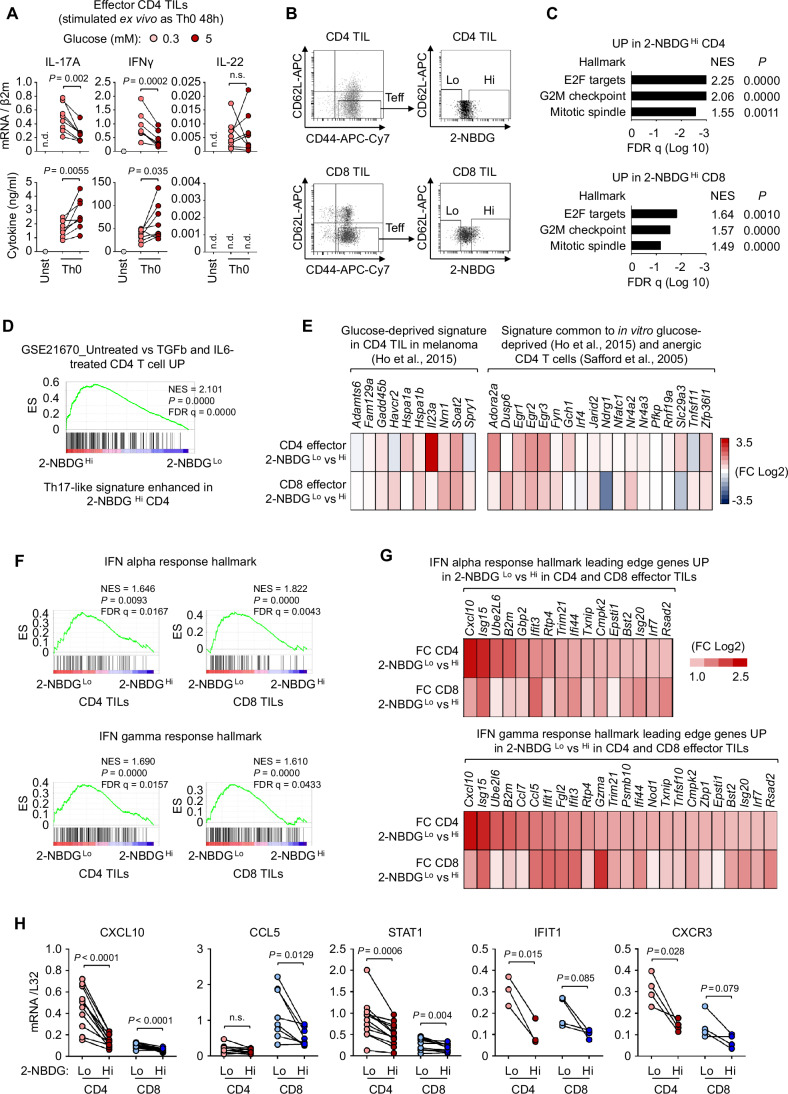
Tumor-infiltrating CD4 and CD8 effector T lymphocytes with reduced uptake of 2-NBDG in vivo exhibit an enhanced IFN response signature. (**A**) Upper panels show the mRNA expression of the indicated gene products in CD4 effector TILs freshly isolated by FACS sorting from LLC tumors and either left unstimulated or stimulated with anti-CD3 and anti-CD28 plus IL-2 (Th0 conditions) 48 h in medium with 5 mM or 0.3 mM glucose. The bottom panels show the protein levels of each cytokine in the supernatants of the same cultures, analyzed by Legendplex bead arrays. IL-22 protein was analyzed but was not reliably detected (n.d.), with values near the detection limit of the assay. Unstimulated controls were kept in medium with 5 mM glucose and IL-2 but without anti-CD3 and anti-CD28 antibodies. This experiment used TILs not traced with 2-NBDG nor Hoechst. Results show 8 biological replicates (8 mice). Statistical significance was assessed by a paired *t* test to compare the two conditions (0.3 and 5 mM glucose) for each sample. (**B**) Flow cytometry analysis of effector (CD44 ^+^, CD62L ^neg^) CD4 and CD8 TILs from LLC tumors in 2-NBDG-injected mice, gated as 2-NBDG ^Lo^ and ^Hi^. The sample shown is one of the four that were FACS-sorted for RNA-seq. (**C**) GSEA of RNA-seq data (GEO dataset GSE250248, this study) shows enriched hallmarks associated with cell division in 2-NBDG ^Hi^ CD4 and CD8 effector TILs. (**D**) GSEA of RNA-seq data using the Immunological gene set collection database of MSigDB identifies a Th17-like signature (CD4 T lymphocytes treated with TGFβ and IL-6) enriched in 2-NBDG ^Hi^ vs 2-NBDG ^Lo^ effector CD4 TILs. (**E**) Expression of gene signatures previously associated with glucose-deprived CD4 TILs is enhanced in 2-NBDG ^Lo^ vs ^Hi^ effector CD4 and CD8 TILs (GEO dataset GSE250248, this study). (**F**) GSEA of RNA-seq data shows upregulated IFN alpha and gamma response hallmarks in 2-NBDG ^Lo^ CD4 and CD8 effector TILs. (**G**) IFN alpha and IFN gamma response hallmark genes with highest differential expression (fold change Log2 > 1) in both CD4 and CD8 2-NBDG ^Lo^ vs 2-NBDG ^Hi^ effector TILs in the RNA-seq data. (**H**) Expression of the indicated genes in effector CD4 and CD8 TILs sorted by their 2-NBDG uptake in vivo. Results are from 3 to 12 biological replicates. Statistical significance was assessed by a paired *t* test to compare the two conditions (0.3 and 5 mM glucose) for each sample. Significant *P* values (<0.05) and *P* values between 0.05 and 0.1 are indicated. n.s. not significant. (**C**, **D**, **F**) The indicated statistics were computed by the GSEA program: ES enrichment score, NES normalized enrichment score, *P* nominal *P* value, FDR false discovery rate. Results in (**C**–**G**) are from one RNA-seq analysis. FC fold-change. Source data are available online for this figure.

Our previous experiments showed that tumor-inexperienced T cells were capable of substantial activity in glucose-restricted conditions in vitro, and then we asked about the cytokine production capacity of effector CD4 tumor-infiltrating T cells (TILs) under normal or low glucose (examples of the gating strategy used for TIL subsets are shown in Figs EV2A,C and 5A). CD62L ^neg^ CD44 ^+^ effector memory CD4 TILs (abbreviated as effector) isolated from subcutaneously implanted Lewis lung carcinoma (LLC) tumors were stimulated as Th0 in 5 mM or 0.3 mM glucose. TILs stimulated in normal glucose expressed less mRNA of IFNγ and IL-17A, but produced more of both proteins than in 0.3 mM glucose at the 48-h time point analyzed (Fig. 3A). Activated TILs also produced more IL-2 and TNFα in 5 mM than 0.3 mM glucose (Fig. EV2B). Overall, we found that TILs in the LLC tumor model displayed a Th1-like profile and had much lower expression of Th17 cytokines. TILs in normal glucose also produced more cytokine protein than in low glucose, but production in low glucose was still substantial with respect to unstimulated conditions, similarly to what we had seen with lymph node T cells (Figs. 1A,D and  EV1A).

Next, we tried to identify TILs with limited access to glucose in vivo, and isolated effector CD4 and CD8 TILs based on their uptake of the 2-deoxyglucose analog 2-NBDG injected intravenously (Fig. EV2C). We initially chose this approach based on multiple articles that had used 2-NBDG uptake by T lymphocytes as a proxy for glucose uptake (Watson et al, 2021; Klein Geltink et al, 2017; O’Sullivan et al, 2021; Gemta et al, 2019; Siska et al, 2017; Scharping et al, 2021; Sukumar et al, 2013). However, as we will discuss below, later works have shown that 2-NBDG and glucose are captured by T lymphocytes through different mechanisms (Sinclair et al, 2020; Pelgrom et al, 2023; Reinfeld et al, 2021). Nonetheless, as 2-NBDG uptake is thought to correlate with the metabolic activity of T cells (Sinclair et al, 2020), we reasoned that this analysis could be useful to identify different subsets of effector TILs in vivo.

We did RNA-seq of effector CD4 and CD8 TILs sorted into two populations: cells in the 15% highest 2-NBDG uptake, and those in the 15% lowest (2-NBDG ^Hi^ and 2-NBDG ^Lo^ respectively) (Fig. 3B). Gene set enrichment analysis (GSEA) suggested that 2-NBDG ^Hi^ cells, both CD4 and CD8, had higher expression of hallmarks associated with cell division and proliferation than 2-NBDG ^Lo^ cells, with upregulated E2F target genes, and G2M checkpoint and mitotic spindle hallmarks (Fig. 3C). We also found an enhanced Th17-like signature in 2-NBDG ^Hi^ versus 2-NBDG ^Lo^ CD4 cells, based on their similarity with T lymphocytes differentiated in vitro with TFGβ and IL-6 (GSE21670, (Durant et al, 2010)) (Fig. 3D). However, other characteristic Th17 markers such as IL-23R, IL-17A or IL-22 were expressed at very low levels in CD4 TILs in their steady state in the tumor, and were poorly detected in independent RT-qPCR mRNA analyses. Regarding TILs with poorer 2-NBDG uptake, they showed increased mRNA expression of several markers previously associated with glucose-limited TILs, and common between anergic and glucose-restricted T lymphocytes (Safford et al, 2005; Ho et al, 2015) (Fig. 3E).

### Effector TILs with reduced uptake of 2-NBDG in vivo show an enhanced type I interferon response signature

The GSEA also uncovered the upregulation of a set of genes common to both IFN alpha (type I, or IFN-I) and IFN gamma responses in 2-NBDG ^Lo^ CD4 and CD8 effector TILs (Fig. 3F,G). We confirmed higher mRNA expression of IFN response genes CXCL10, CCL5, STAT1 and IFIT1 in 2-NBDG ^Lo^ cells, and also found that they expressed more CXCR3 mRNA than 2-NBDG ^Hi^ cells (Fig. 3H). CXCR3 is the receptor of IFN-induced chemokines CXCL9, CXCL10 and CXCL11, and activation of CXCR3 signaling in CD8 TILs has been shown to enhance their antitumor function and anti-PD-1-mediated activity (Peng et al, 2015; Chow et al, 2019; Dangaj et al, 2019). Additional experiments in mice lacking the type I IFN (IFN-I) receptor (IFNAR1) showed that expression of IFIT1, STAT1, and CXCL10 mRNA was highly dependent on IFN-I signaling, whereas CXCR3 and CCL5 were less dependent or mostly independent (Fig. EV2D). These findings showed that a distinct feature of 2-NBDG ^Lo^ TILs was their enhanced expression of specific IFN-I target genes.

### Tumor-associated myeloid cells with reduced uptake of 2-NBDG in vivo show elevated expression of type I interferons

**Figure 4:**
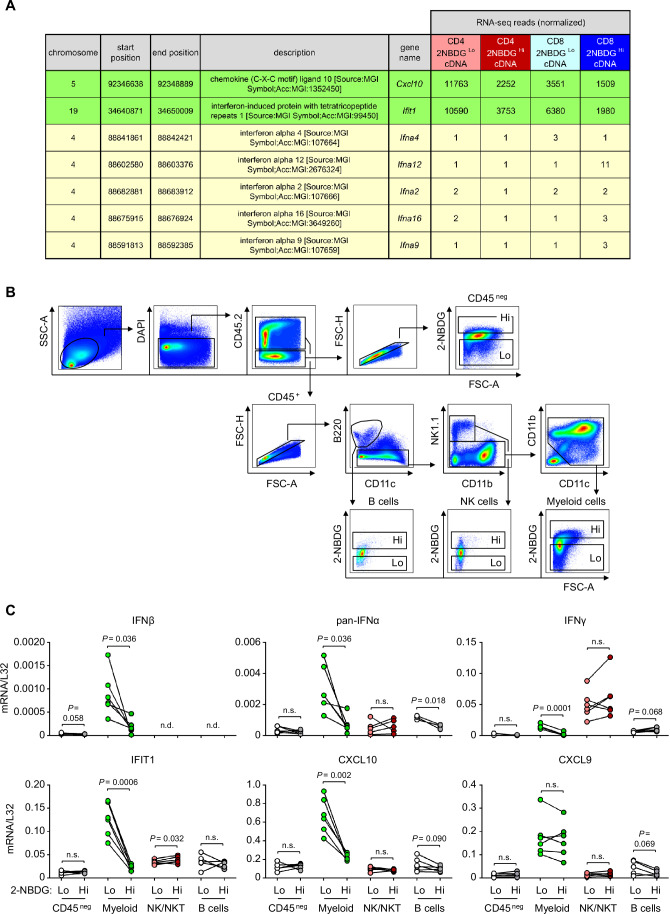
Myeloid cells in poorly perfused tumor regions express higher levels of type I IFNs (IFNβ and IFNα) and ISGs than those in more accessible regions. (**A**) Expression of type I IFN genes was not detected in effector CD4 and CD8 TILs sorted by their 2-NBDG uptake (data from GEO dataset GSE250248, this study). *Cxcl10* and *Ifit1* are included for comparison, as highly expressed IFN-I-response genes with differential expression between 2-NBDG ^Lo^ vs ^Hi^ effector TILs (see Figs. 3H and EV2D). (**B**) Flow cytometry gating strategy for isolating different cell populations in LLC tumors based on their uptake of intravenously injected 2-NBDG. (**C**) mRNA levels of IFNs and IFN-stimulated genes (ISGs) in CD45^neg^, myeloid, NK/NKT, and B cells, FACS-sorted as 2-NBDG ^Lo^ or ^Hi^. Results show 6 individual tumors (biological replicates) from one experiment. Statistical significance was assessed with a paired *t* test to compare ^Lo^ and ^Hi^ cells within the same tumor. Significant *P* values (<0.05) and *P* values between 0.05 and 0.1 are indicated. n.s. not significant. Source data are available online for this figure.

We then explored possible sources of IFN-I in the tumor microenvironment. We had seen that the mRNAs of IFN-I-responsive genes were hardly represented in the RNA-seq data of effector TILs (GSE250248) (Fig. 4A), suggesting that TILs themselves were very poor IFN-I producers. Therefore, we sorted cells from tumors labeled with 2-NBDG in vivo, into 2-NBDG ^Lo^ and ^Hi^ populations: CD45-negative cells, which would comprise tumor cells and stroma, myeloid cells (pooled as CD11b ^+^ CD11c ^+^ cells), NK and NKT cells (pooled as NK1.1 ^+^), and CD11c ^neg^ B220 ^+^ B cells (Fig. 4B). We found that IFNβ1 and IFNα were much more expressed in myeloid cells than in the other populations, and their expression was significantly higher in the 2-NBDG ^Lo^ fraction (Fig. 4C). Likewise, 2-NBDG ^Lo^ myeloid cells also expressed the highest levels of the IFN-I-induced gene products IFIT1, CXCL10, and CXCL9. B cells and NK/NKT cells expressed low levels of IFNα but not IFNβ1, and NK/NKT cells had the highest expression of IFNγ (Fig. 4C), something expected since NK, NKT and T cells are main IFNγ producers. Altogether, these results showed that tumor regions with poor uptake of 2-NBDG harbored populations of IFN-I-producing myeloid cells, and suggested that their expression of IFNβ1 and IFNα stimulated the enhanced IFN-I response we had identified in 2-NBDG ^Lo^ TILs.

### Comparison of TILs by their in vivo uptake of intravenously delivered probes 2-NBDG and Hoechst 33342

Our results identified distinct characteristics in TIL subsets differing in their in vivo uptake of 2-NBDG. Although some features of TILs with poorer 2-NBDG uptake resembled those identified in previous studies with glucose-restricted T cells (Safford et al, 2005; Ho et al, 2015), it is likely that in vivo, other nutrients and metabolites besides glucose will be limited in the tumor microenvironment. Also, recent works had shown that 2-NBDG cannot really be considered equivalent to glucose (Sinclair et al, 2020; Pelgrom et al, 2023; Reinfeld et al, 2021), and we wondered whether its uptake by TILs could parallel that of glucose or was affected by other variables. Here, we reasoned that a relevant factor influencing 2-NBDG uptake by TILs in vivo could be their accessibility to blood circulation in the tumor.

**Figure 5:**
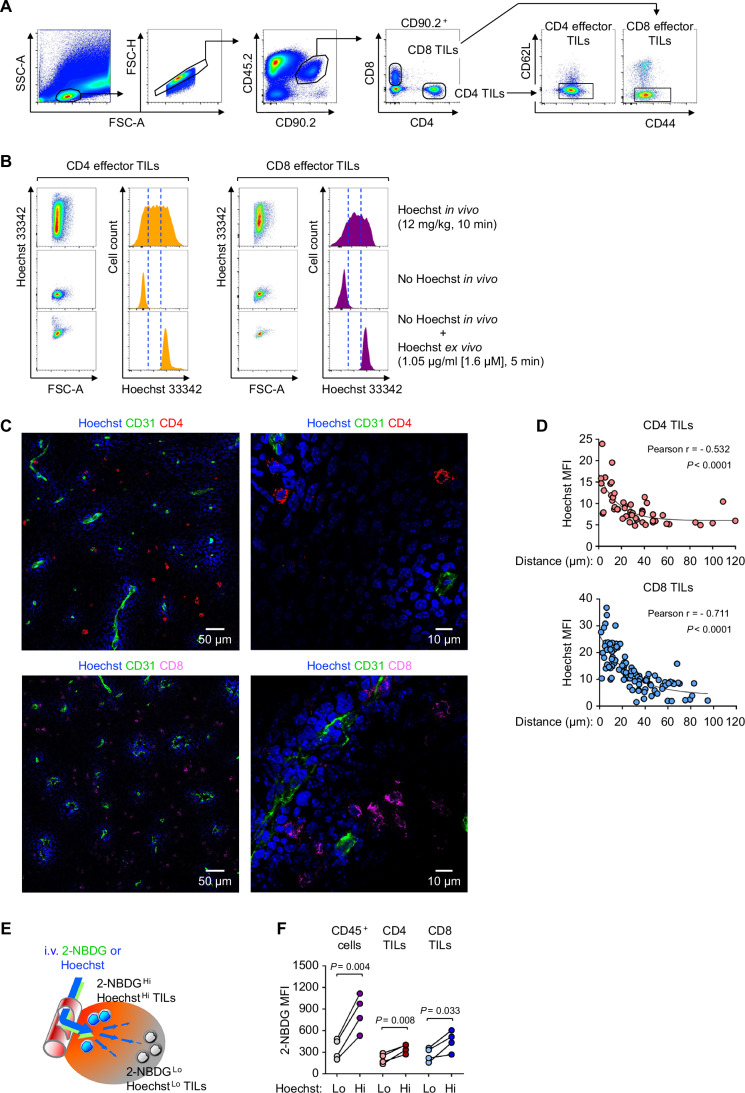
In vivo uptake of intravenously injected Hoechst 33342 by CD4 and CD8 TILs is inversely correlated with their distance from blood vessels. (**A**) Flow cytometry gating strategy to identify CD4 and CD8 effector T lymphocytes infiltrating LLC tumors. (**B**) (Upper panels) flow cytometry analysis of the in vivo uptake of intravenously injected Hoechst 33342 by CD4 and CD8 effector TILs. (Middle panels) background fluorescence in the Hoechst detection channel in TILs from mice that were not injected with Hoechst. (Bottom panels) ex vivo uptake of Hoechst by TILs in cell suspensions from freshly isolated and disaggregated tumors. Results shown are from 2 mice, one mouse with LLC tumor was injected with Hoechst, and another mouse only with PBS. TILs in this latter tumor were used to set up the gating panels in (**A**), the background Hoechst fluorescence in the middle panel in (**B**), and the ex vivo Hoechst uptake in the bottom panel in (**B**). (**C**) Confocal microscopy images showing the distribution of intravenously injected Hoechst 33342 (blue) in LLC tumor sections, together with endothelial cells (blood vessels, CD31 cells in green), CD4 (upper panels, in red), and CD8 (bottom panels, in magenta). Images are shown in two magnifications (scale bars are included), with a wider tumor section shown in the left panels, and a narrower section at higher magnification in the right panels to better visualize the CD4 and CD8 TILs. Results are representative from two biological replicates (2 mice). (**D**) The graphics show the quantification of Hoechst’s mean fluorescence intensity (MFI) of TILs with respect to their distance to the nearest vessel, done in sections of two biological tumor replicates (independent mice), with 25 cells in one tumor and 26 in another for CD4 cells, and 67 cells in one tumor and 35 in another for CD8 cells. Results are from 2 biological replicates (2 mice). The curved line shows the data fit for a non-linear fit, one-phase decay equation. The Pearson correlation coefficient and its *P* value are shown. (**E**) Diagram of the experiment, in which mice were injected with a mixture of 2-NBDG and Hoechst 33342 intravenously to analyze the simultaneous uptake of both probes. (**F**) Correlation between the uptake of Hoechst 33342 and 2-NBDG in immune cells (CD45 ^+^) or effector CD4 and CD8 T lymphocytes in LLC tumors, 10 min after intravenous injection of the probes. 2-NBDG fluorescence was quantified in the 15% lowest or highest Hoechst-stained populations. Result is from four biological replicates (4 mice). Dots are connected by lines to better visualize differences between Hoechst ^Lo^ vs ^Hi^ cells within each individual tumor. Statistical significance was assessed by a paired *t* test. Significant *P* values (<0.05) are indicated. Source data are available online for this figure.

**Figure EV3:**
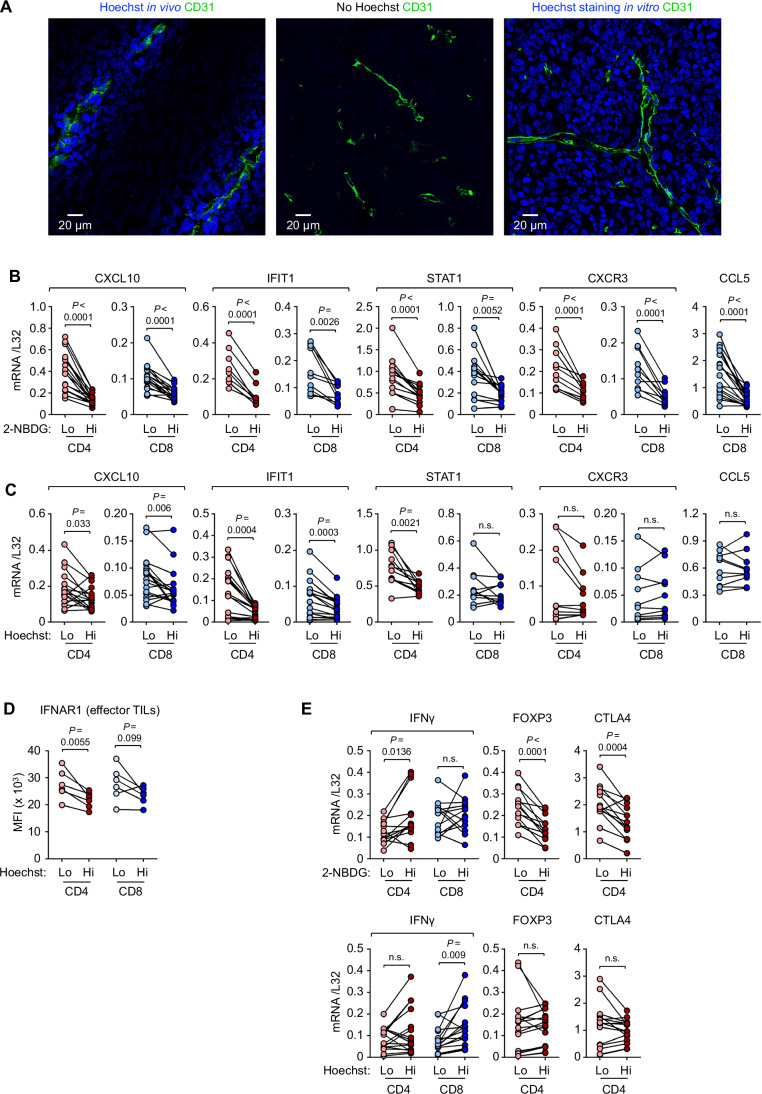
Comparison of CD4 and CD8 effector TILs based on their uptake of 2-NBDG and Hoechst for the expression of the mRNAs of interferon-stimulated genes (ISGs), CXCR3, CCL5, and the expression of IFNγ, FOXP3 and CTLA4 mRNA. (**A**) Confocal microscopy images showing: (left panel) the distribution of intravenously injected Hoechst 33342 (blue) in LLC tumor sections, together with endothelial cells (blood vessels, CD31 cells in green); (middle panel) blood vessels in a tumor section from a mouse not injected with Hoechst; and (right panel), blood vessels in a tumor section from a mouse not injected with Hoechst, but stained with Hoechst in vitro, showing a dense, homogenous distribution of cells (blue nuclei) in the tumor section. Scale bars are shown. Results are representative of two biological replicates (2 mice). (**B**, **C**) mRNA levels of the indicated genes in CD4 and CD8 effector TILs sorted as 2-NBDG ^Lo^ versus ^Hi^ cells (**B**) or Hoechst ^Lo^ versus ^Hi^ cells (C). Results in (**B**) comprise 9 to 18 biological replicates (3 to 5 experiments with 3 to 4 mice each). Results in (C) comprise 10 to 17 biological replicates (2 to 3 experiments with 4 to 7 mice each). (**B**) includes samples from the experiments shown in Fig. 3H plus additional ones, and (**C**) includes samples from experiments shown in Fig. EV5H (vehicle-treated mice) plus additional ones. Dots are connected by lines to better visualize differences between ^Lo^ vs ^Hi^ cells within each individual tumor. Statistical significance was assessed with a paired *t* test to compare ^Lo^ versus ^Hi^ cells within same tumor. (**D**) Surface expression of the IFN-I receptor chain IFNAR1 in Hoechst ^Lo^ versus ^Hi^ CD4 and CD8 effector TILs. Results are from 6 biological replicates (individual mice). (**E**) mRNA levels of IFNγ, FOXP3 and CTLA4 in CD4 and CD8 effector TILs sorted as 2-NBDG ^Lo^ versus ^Hi^ cells (upper panels) or Hoechst ^Lo^ versus ^Hi^ cells (lower panels). Results for 2-NBDG comprise 13 biological replicates (4 experiments with 3 to 4 mice each). Results for Hoechst comprise 15 to 16 biological replicates (3 experiments with 4 to 7 mice each). Data for 2-NBDG include samples from the experiments shown in Fig. 3H plus additional ones, and Hoechst data include samples from experiments shown in Fig. EV5H (vehicle-treated mice) plus additional ones. Dots are connected by lines to better visualize differences between ^Lo^ versus ^Hi^ cells within each individual tumor. Statistical significance was assessed with a paired *t* test to compare ^Lo^ and ^Hi^ cells within same tumor. Significant *P* values (<0.05) and *P* values between 0.05 and 0.1 are indicated. n.s. not significant. Source data are available online for this figure.

To assess this we compared 2-NBDG with an independent approach, using the DNA dye Hoechst 33342, which, when delivered intravenously, labels cells in tumors proportionally to their accessibility to blood vessels (Huang et al, 2012; Chaplin et al, 1985; Schroeder et al, 2005; Kumar et al, 2020; Zheng et al, 2018; Kumar et al, 2019). This approach showed that shortly after injecting Hoechst intravenously, effector TILs acquired the dye in a gradient-like distribution that spanned from highly labeled to essentially unlabeled cells (Fig. 5A,B). In contrast to the heterogenous Hoechst labeling in vivo, parallel labeling ex vivo of disaggregated cells from freshly excised tumors produced a much more homogeneous staining of effector TILs (Fig. 5B), suggesting that the highly dispersed uptake of Hoechst in vivo was due to the distribution of cells with respect to the tumor blood vessels rather than cell-intrinsic differences to capture Hoechst. To further validate this approach, we used confocal microscopy to quantify the uptake of intravenously-injected Hoechst 33342 by CD4 and CD8 TILs located in the proximity of blood vessels or farther from them (Figs. 5C,D and  EV3A). Our results showed that the Hoechst signal was highest near vessels (identified by the endothelial cell marker CD31) and decreased steeply with distance (Figs. 5C and  EV3A). Likewise, CD4 and CD8 TILs showed a clear and significant inverse correlation between Hoechst uptake and distance from blood vessels, and by 40 μm away from the nearest vessel, TILs were essentially unlabeled (Fig. 5D). Finally, we show that in mice injected intravenously with a mixture of both probes (Fig. 5E), the bulk of CD45 ^+^ leukocytes in the tumor, as well as effector CD4 and CD8 TILs showed a good correlation between 2-NBDG and Hoechst uptake (Fig. 5F), indicating that uptake of 2-NBDG was higher in tumor-infiltrating leukocytes with better accessibility to blood.

Having found a significant within-tumor correlation between Hoechst 33342 and 2-NBDG in leukocytes and TILs, we wanted to confirm that the enhanced IFN-I response signature that we had identified as a distinctive feature of 2-NBDG ^Lo^ TILs was also found in Hoechst ^Lo^ cells. We found that CXCL10, IFIT1 and STAT1 were more expressed in Hoechst ^Lo^ CD4 and CD8 effector TILs than in their Hoechst ^Hi^ counterparts, as we had seen earlier for 2-NBDG ^Lo^ cells, confirming that TILs in less perfused regions (Hoechst ^Lo^) had elevated expression of IFNAR1-dependent IFN-I response gene products CXCL10, IFIT1 and STAT1 (Fig. EV3B,C). However, expression of CXCR3 and CCL5 mRNA did not show consistent differences between Hoechst ^Lo^ and ^Hi^ cells (Fig. EV3B,C). We also found that the IFN-I receptor IFNAR1 had moderately higher surface expression in Hoechst ^Lo^ effector TILs (Fig. EV3D). These results aligned with those in Figs. 3 and  4, suggesting that the enhanced IFN-I response of TILs in less accessible tumor regions resulted from a higher IFN-I production by myeloid cells and higher intrinsic IFNAR1 expression in TILs.

We now asked whether effector TILs isolated from low accessibility regions in vivo differed in their expression of markers associated with tumor-facilitating Treg cells, or with antitumor activity, such as IFNγ. Recent work has shown that FOXP3 ^+^ (Treg) cells isolated from mouse B16 melanoma tumors cultured ex vivo exhibit lower intrinsic 2-NBDG uptake capacity than non-Treg cells (Watson et al, 2021). Our in vivo experiments comparing TILs with low versus high probe uptake within each individual tumor showed that 2-NBDG ^Hi^ CD4 effector TILs had moderately higher IFNγ mRNA levels than their 2-NBDG ^Lo^ counterparts (Fig. EV3E), whereas CD8 effector TILs in both ^Lo^ and ^Hi^ compartments expressed it comparably. By contrast, in the Hoechst experiments it was Hoechst ^Hi^ CD8 effector TILs that expressed more IFNγ mRNA (Fig. EV3E). Regarding FOXP3 and CTLA4 mRNA in CD4 effector TILs, both showed higher expression in 2-NBDG ^Lo^ cells, but were more similarly expressed in Hoechst ^Lo^ and ^Hi^ cells (Fig. EV3E). These differences could suggest that Hoechst uptake mainly depended on accessibility to blood, whereas the uptake of 2-NBDG in vivo might depend on both the accessibility of TILs to blood and other characteristics, such as their polarization state, in line with previous findings that Treg TILs cultured ex vivo have lower cell-intrinsic 2-NBDG uptake capacity than non-Treg TILs (Watson et al, 2021).

**Figure 6:**
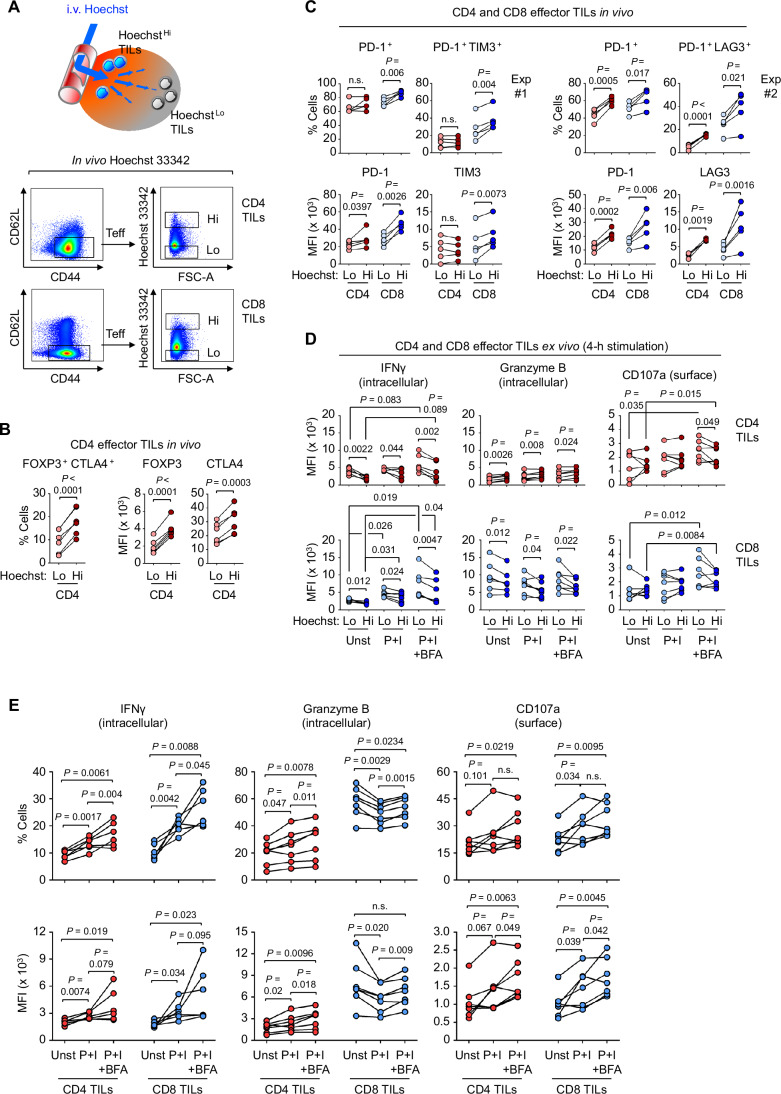
Comparison of activation, exhaustion and T regulatory features in TILs with reduced or higher uptake of Hoechst 33342 in vivo. (**A**) Diagram illustrating the experiment, and flow cytometry strategy to detect effector memory (Teff) CD4 and CD8 TILs in LLC tumors, and their in vivo uptake of intravenously injected Hoechst. (**B**, **C**) Expression of intracellular Treg proteins FOXP3 and CTLA4 (**B**) and exhaustion surface markers PD-1, TIM3 and LAG3 (**C**) in Hoechst ^Lo^ versus ^Hi^ CD4 and CD8 effector TILs. Expression of the proteins analyzed is shown as % of positive cells and mean fluorescence intensity (MFI), quantified in the 15% lowest or highest Hoechst-stained populations by flow cytometry. Results correspond to 6 biological replicates (**B**), five biological replicates (**C**, Exp #1, left panels) and five biological replicates (**C**, Exp #2, right panels). (**D**) Expression of IFNγ, granzyme B and CD107a analyzed by flow cytometry in effector CD4 and CD8 Hoechst ^Lo^ and ^Hi^ TILs is shown as mean fluorescence intensity (MFI), quantified in the 15% lowest or highest Hoechst-stained populations. Results correspond to six to seven biological replicates. PMA plus ionomycin (P + I), brefeldin A (BFA). (**E**) Expression of IFNγ, granzyme B, and CD107a analyzed by flow cytometry in the same CD4 and CD8 effector TILs as above, but without separating them into Hoechst ^Hi^ and ^Lo^ cells, shown as % cells and MFI. Results correspond to six to seven biological replicates. Statistical significance in (**B**–**E**) was assessed with a paired *t* test. Significant *P* values (<0.05) and *P* values between 0.05 and 0.1 are indicated. n.s. not significant. Source data are available online for this figure.

As these mRNA analyses were not fully conclusive as to the expression of IFNγ and Treg- markers, we analyzed their protein levels. These experiments were done with cells labeled with Hoechst in vivo (Fig. 6A), but not 2-NBDG because the permeabilization needed for intracellular protein detection caused the loss of 2-NBDG signal. We found that Hoechst ^Hi^ CD4 effector TILs expressed more FOXP3 and CTLA4 than their Hoechst ^Lo^ counterparts in the same tumor (Fig. 6B). Hoechst ^Hi^ effector TILs in vivo also expressed more surface PD-1, and the exhaustion-associated markers TIM3 (CD8) and LAG3 (both CD4 and CD8) than Hoechst ^Lo^ cells (Fig. 6C). We then assessed the ability to induce IFNγ protein, and the expression of markers of cytotoxic capacity granzyme B and CD107a in TILs (Fig. 6D,E). Hoechst ^Lo^ CD4 and CD8 effector TILs induced slightly more of the cytokine upon activation (Fig. 6D). This result suggested that TILs in low-accessibility tumor regions were as intrinsically capable of rapid response to stimulation as those with better accessibility to blood. As to how Hoechst ^Lo^ TILs produced more IFNγ protein than Hoechst ^Hi^ cells, this might be due to their having gone from the tumor microenvironment to a richer culture medium. Nonetheless, as shown in our longer-term (48 h) stimulation experiments in Fig. 3A, prolonged nutrient restriction would decrease their cytokine output. Our analysis also detected that Hoechst ^Lo^ and ^Hi^ CD8 effector TILs had quite similar granzyme B levels, and mobilized the lytic granule marker CD107a comparably upon ex vivo stimulation (Fig. 6D). Altogether, these results suggest that Hoechst ^Lo^ and ^Hi^ CD4 and CD8 effector TILs had comparable intrinsic competency to Hoechst ^Hi^ TILs for eliciting Th1 and cytotoxic responses. However, their actual activity in vivo might differ because they express different levels of immunomodulatory receptors (PD-1, CTLA4, TIM3, LAG3) to interact with other cells in their microenvironment. In this regard, our result that the population of Hoechst ^Hi^ TILs had more Treg cells and higher expression of immune checkpoints and exhaustion markers suggested that T cells in regions with better access to blood, and presumably nutrients, might experience greater pressure by immune inhibitory mechanisms.

### Sensitivity of effector TILs to acetyl-CoA synthetase 2 inhibitors

**Figure EV4:**
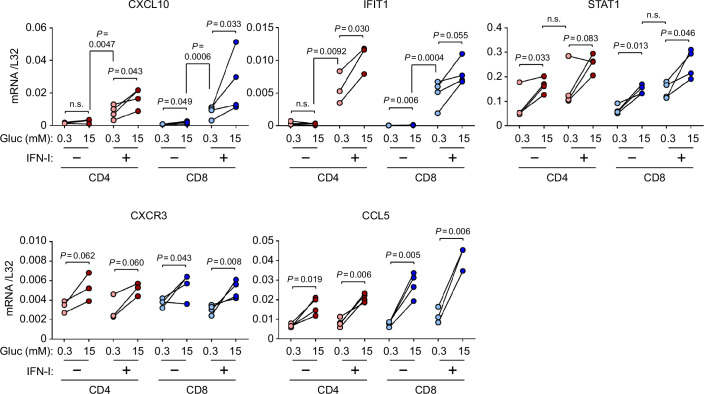
Response to IFN-I and glucose sensitivity of different genes in in vitro-activated lymph node T lymphocytes. mRNA expression of the indicated genes in T lymphocytes preactivated in vitro (5 days) and restimulated (24 h) with anti-CD3 and anti-CD28 without or with a cocktail of IFNα4 (600 U/ml) and IFNβ1 (2.5 ng/ml) (IFN-I) in culture medium with 0.3 or 15 mM glucose. T lymphocyte cultures were prepared from three to four biological replicates (independent mice). Statistical significance was assessed with a paired *t* test. Significant *P* values (<0.05) and *P* values between 0.05 and 0.1 are indicated. n.s. not significant. Source data are available online for this figure.

We wondered whether the expression of IFN-I response genes in activated T lymphocytes was regulated by glucose. In vitro assays showed that CXCL10 and IFIT1, both highly responsive to IFN-I, as well as the less IFN-I-responsive STAT1, CXCR3 and CCL5 could be induced in low glucose medium (0.3 mM), but were expressed better when cells had more available glucose (Fig. EV4). These experiments also revealed that adding exogenous IFN-I in a glucose-poor environment induced higher expression of IFN-stimulated genes (ISGs) CXCL10 and IFIT1 than just stimulating the cells in normal glucose but without extra IFN-I.

**Figure EV5:**
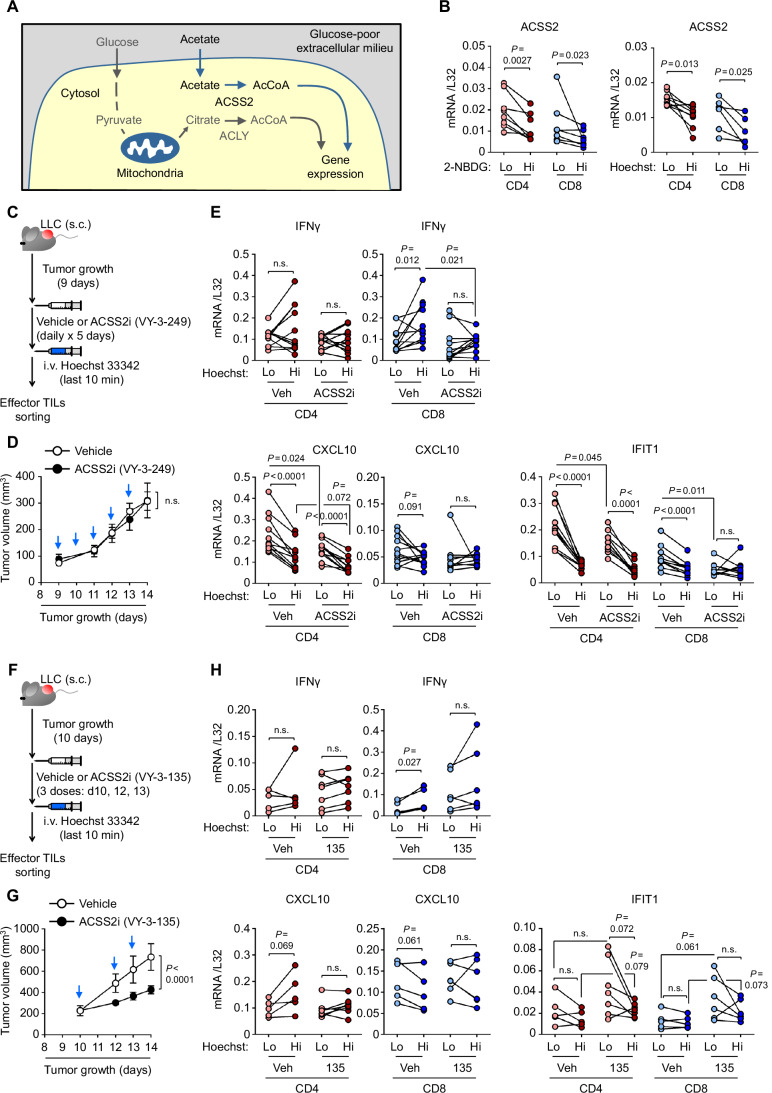
ACSS2 inhibitors have different effects on gene expression in CD4 and CD8 TILs with high or reduced access to blood in vivo. (**A**) Diagram illustrating that T cells in a glucose-poor environment can obtain cytosolic acetyl-CoA (AcCoA) from imported extracellular acetate by using the enzyme acetyl-CoA-synthetase 2 (ACSS2). Glucose normally contributes to cytosolic AcCoA when the glycolysis product pyruvate is processed through the mitochondrial Krebs cycle, from which citrate exported to the cytosol is converted to AcCoA by ATP citrate lyase (ACLY) (diagram concept adapted from Qiu et al, 2019). (**B**) Expression of ACSS2 mRNA in CD4 and CD8 TILs sorted by their uptake of 2-NBDG or Hoechst 33342. Results in (**B**) are from six to eight biological replicates (from two independent experiments for 2-NBDG and Hoechst, with 3 to 6 mice per experiment). (**C**, **D**) Diagram of the in vivo experiment (**C**) and tumor growth in mice treated with vehicle or ACSS2i/VY-3-249 (25 mg/kg) (**D**), blue arrows indicate when the mice were treated. (**E**) mRNA expression of the indicated gene products in CD4 and CD8 effector TILs from mice left untreated or treated with vehicle or ACSS2i/VY-3-249 (ACSS2i), and then sorted by their uptake of Hoechst in vivo. Results in (**D**, **E**) are from 9 to 11 biological replicates (mice) per experimental group (2 independent experiments with 4 and 7 mice each). (**F**, **G**) Diagram of the in vivo experiment (**F**) and tumor growth in mice treated with vehicle or VY-3-135 (100 mg/kg) (**G**), blue arrows indicate when the mice were treated. (**H**) mRNA expression of the indicated gene products in CD4 and CD8 effector TILs from mice left untreated or treated with vehicle or VY-3-135 (abbreviated as 135), and then sorted by their uptake of Hoechst in vivo. Results in (**G**, **H**) are from 5 to 7 biological replicates (mice) per experimental group (1 experiment). Statistical significance was assessed with a paired *t* test (**B**, **E**, **H**) and a two-way ANOVA test (**D**, **G**). Significant *P* values (<0.05) and *P* values between 0.05 and 0.1 are indicated. n.s. not significant. Source data are available online for this figure.

These results suggested that either sufficient glucose to induce IFN-I response genes might be available to TILs in regions with poor blood accessibility (2-NBDG ^Lo^ and Hoechst ^Lo^), or that cells could use alternative metabolites in this microenvironment. Both possibilities would agree with recent works, as it has been shown that TILs might be able to capture sufficient glucose to maintain their functionality (Reinfeld et al, 2021), and also use imported acetate abundant in the tumor microenvironment to produce acetyl-CoA, needed to transcribe several glucose-dependent genes, and thus compensate for a reduction in endogenous glucose-derived acetate (Qiu et al, 2019; Peng et al, 2016) (illustrated in the diagram of Fig. EV5A, adapted from Qiu et al, 2019).

**Figure 7:**
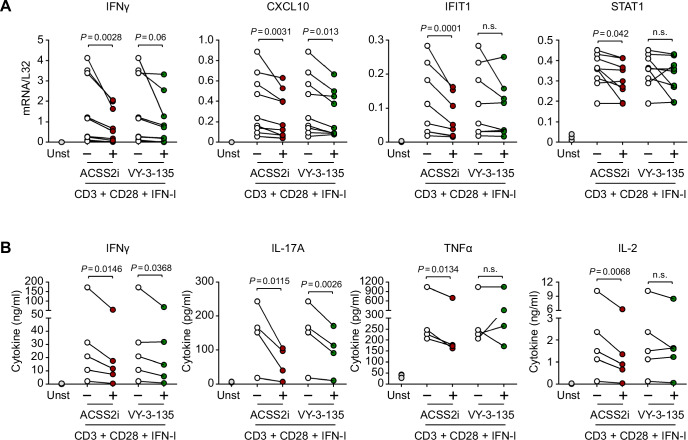
Sensitivity to ACSS2 inhibitors of T cell receptor- and IFN-I-induced ISGs and cytokines in CD4 effector TILs. (**A**, **B**) FACS-sorted CD4 effector TILs from LLC tumors were left unstimulated (Unst) or stimulated with anti-CD3 plus anti-CD28 and a mixture of IFNα4 (600 U/ml) and IFNβ1 (2.5 ng/ml) (IFN-I) for 24 h in medium with 5 mM glucose in the absence or presence of 10 µM ACSS2 inhibitors ACSS2i/VY-3-249 (ACSS2i) or VY-3-135 (135). (**A**) mRNA levels of IFNγ and ISGs analyzed in 3 (unstimulated cells) and 8–9 (CD3 and CD28-stimulated cells) biological replicates (from individual tumors). (**B**) Cytokine concentration in the culture supernatants of 4–5 of the samples analyzed in (**A**), measured by Legendplex bead array. Statistical significance was assessed with a paired *t* test. Significant *P* values (<0.05), and *P* values between 0.05 and 0.1 are indicated. n.s. not significant. Source data are available online for this figure.

We noticed that both 2-NBDG ^Lo^ and Hoechst ^Lo^ effector TILs expressed more acetyl-CoA synthetase 2 (ACSS2) mRNA than their 2-NBDG ^Hi^ and Hoechst ^Hi^ counterparts (Fig. EV5B). ACSS2 is the enzyme that produces acetyl-CoA from imported acetate (Qiu et al, 2019), and we analyzed its contribution to gene expression in effector TILs with different accessibility to blood. For this, we treated LLC tumor-bearing mice with vehicle or two chemically independent ACSS2 inhibitors, ACSS2i (also known as VY-3-249 or HY-104032) (Li et al, 2021), and VY-3-135 (Miller et al, 2023), and then isolated effector CD4 and CD8 TILs by their in vivo uptake of Hoechst 33342 (diagrams in Fig. EV5C,F). In these experiments, ACSS2i/VY-3-249 did not inhibit tumor growth (Fig. EV5D), whereas VY-3-135 did (Fig. EV5G), consistent with published work in other mouse tumor models (Miller et al, 2023). ACSS2i/VY-3-249 mildly inhibited the expression of IFNγ mRNA in Hoechst ^Hi^ CD8 effector TILs, CXCL10 in Hoechst ^Lo^ CD4, and IFIT1 in Hoechst ^Lo^ CD4 and CD8 TILs (Fig. EV5E). By contrast, VY-3-135 did not inhibit any of these genes and even caused a mild trend toward increasing IFIT1 mRNA expression in CD4 and CD8 TILs (Fig. EV5H). Seeing that both ACSS2 inhibitors had different, and modest, effects on TILs in vivo, we tested their effect directly on CD4 effector TILs activated ex vivo with anti-CD3 and anti-CD28 stimulation plus IFNα and β. We found that both compounds inhibited the induction of IFNγ and CXCL10 mRNA comparably, but ACSS2i/VY-3-249 caused a better inhibition of IFIT1, and STAT1 induction was mildly inhibited by ACSS2i/VY-3-249 and not by VY-3-135 (Fig. 7A). Both inhibitors also reduced the production of IFNγ and IL-17A protein, but only ACSS2i/VY-3-249 was moderately inhibitory for TNFα and IL-2 (Fig. 7B). These results indicated that ACSS2 could contribute to the expression of IFNγ, other cytokines, and several ISGs in effector TILs activated via T cell receptor and type I IFNs. However, of the two inhibitors only ACSS2i/VY-3-249 showed a similar effect in vivo and ex vivo, whereas VY-3-135 did not inhibit TILs, and yet it significantly reduced tumor growth. It is possible that these differences in vivo could be due to indirect effects on TILs from other cell types in the tumor microenvironment that could be variably sensitive to the inhibitors.

### TILs from regions with higher and lower blood accessibility can both maintain energy metabolism fundamentally from glucose

**Figure 8:**
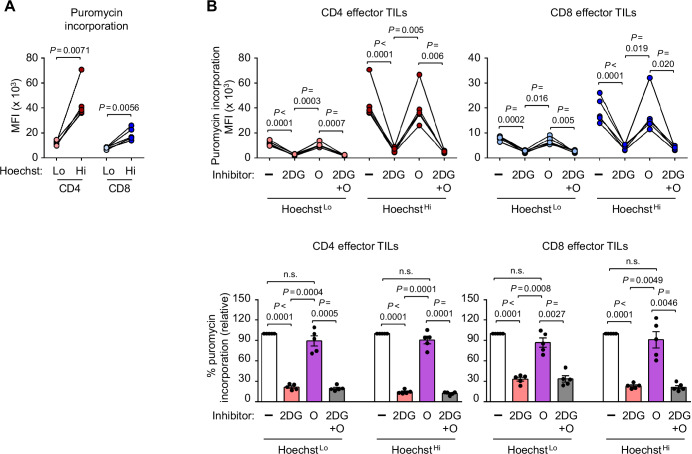
TILs in poorly perfused tumor regions have lower biosynthetic activity than those in more accessible regions, but the energy metabolism of both depends comparably on glycolysis. (**A**) SCENITH puromycin incorporation assay ex vivo in Hoechst ^Lo^ versus ^Hi^ CD4 and CD8 effector TILs freshly isolated from LLC tumors. (**B**) Dependence of protein synthesis (proportional to energy availability) of Hoechst ^Lo^ versus ^Hi^ CD4 and CD8 effector TILs on glycolysis (inhibited with 2-deoxyglucose (2DG)) versus mitochondrial respiration (inhibited with oligomycin (O)). The upper panels show the incorporation of puromycin as mean fluorescence intensity (MFI), and the bottom panels as percentage (mean ± SEM) relative to the control without inhibitors. Results in (**A**, **B**) are from five biological replicates (individual tumors). Statistical significance in (**A**) and upper panel in (**B**) was assessed with a paired *t* test to compare the different treatments within each individual sample. A one-sample *t* test was used in the bottom panels in (**B**) for comparison of each inhibitor with the control sample (100%), and a paired *t* test for comparisons between treatments. Significant *P* values (<0.05) are indicated. Source data are available online for this figure.

Our results showed that effector TILs in poorly perfused tumor regions had features suggestive of their being in a nutrient-limited environment (Fig. 3E), but at the same time seemed competent to express markers associated with antitumor capacity (Fig. 6D), and even had reduced expression of immune checkpoints (Fig. 6C). Therefore, we decided to ask about the dependence of effector CD4 and CD8 TILs on glycolysis and mitochondrial respiration in relation to their better or poorer accessibility to blood, for which we used the SCENITH assay (Argüello et al, 2020). In this assay, the metabolic proficiency of the cells and their dependence on different ATP sources (glycolysis, mitochondrial respiration) are assessed by their rate of puromycin incorporation, a measure of protein translation and biosynthetic capacity (Argüello et al, 2020; Lopes et al, 2021). We found that effector CD4 and CD8 TILs with poorer blood accessibility in vivo were metabolically less active than more accessible cells, as shown by their significantly lower protein translation rate (Fig. 8A), but we also observed that TILs in both low and high accessibility compartments were largely dependent on glucose rather than respiration for energy metabolism (Fig. 8B). This result was similar to our finding that activated T cells would still use glucose as a major substrate for ATP production even in glucose-poor medium (Figs. 1H and  EV1D).

## Discussion

In this work we first studied the capacity of activated T cells to function under substantial restriction, but not absence, of glucose, and then extended our analysis to an in vivo scenario, the tumor microenvironment, where effector T cells can find themselves under very limited access to blood-transported nutrients. We reasoned that TILs more likely to be subjected to a general nutrient deficiency would be found in tumor regions poorly perfused by blood, and used the uptake of intravenously injected fluorescent probes by T cells as an indicator of their accessibility to blood-transported nutrients. This approach has allowed us to isolate and characterize distinct populations of effector CD4 and CD8 TILs in vivo.

First, our in vitro experiments showed that effector CD4 T cells need little glucose to sustain glucose-dependent cytokine expression, energy sufficiency and mTORC1 activity, but present different glucose dependence thresholds for specific cytokines that vary with their activation history. These results are in line with recent work showing that lowering glucose down to 0.35 mM in human CD4 effector T cells severely reduces their proliferation but has a minimal impact on IFNγ production (Ecker et al, 2018). Others have also reported that activated mouse T cells produce comparable IFNγ levels when cultured in 0.6 to 1 mM glucose as in 10 mM (Blagih et al, 2015; Ho et al, 2015). Likewise, we found that TILs activated ex vivo in low-glucose medium produced lower amounts of several cytokines (IFNγ, IL-17A, IL-2 and TNFα) than in normal glucose, but still could produce substantial amounts of cytokine proteins, similarly to what we had seen with lymph node T cells. Nonetheless, in our in vitro experiments we had only modified the concentration of one nutrient, glucose, whereas in vivo, other nutrients and metabolites besides glucose will be limited in the tumor microenvironment. Therefore, we asked which characteristics could be expected in TILs with reduced access to blood in the tumor microenvironment.

We show that the uptake of intravenously injected Hoechst 33342 identifies CD4 and CD8 TILs close to (higher Hoechst uptake) or distant from blood vessels; and in vivo experiments showed a significant correlation between the uptake of Hoechst and the deoxyglucose analog 2-NBDG in tumor-infiltrating leukocytes and effector CD4 and CD8 T cells. This suggests that 2-NBDG uptake by TILs, at least in part, could parallel their general accessibility to blood-transported nutrients. When we initiated this approach, 2-NBDG uptake by T lymphocytes had been widely used as equivalent to glucose uptake (Watson et al, 2021; Klein Geltink et al, 2017; O’Sullivan et al, 2021; Gemta et al, 2019; Siska et al, 2017; Scharping et al, 2021; Sukumar et al, 2013), but later works showed that 2-NBDG and glucose are captured by T lymphocytes through different mechanisms (Sinclair et al, 2020; Pelgrom et al, 2023; Reinfeld et al, 2021). Nonetheless, our results indicate that some characteristics of effector TILs with poor uptake of 2-NBDG, such as the downregulation of cell proliferation and cell cycle-related gene hallmarks, remind of T lymphocytes with restricted access to blood-transported glucose and other nutrients (Ho et al, 2015; Safford et al, 2005; Watson et al, 2021). In this regard, it is likely that the phenotype we have seen in TILs with poor uptake of blood-delivered probes could result from their being exposed to a complex metabolic microenvironment (Sullivan et al, 2019; Lim et al, 2020; Chapman and Chi, 2022; Reinfeld et al, 2021), and not just one low in glucose.

Our observation that the mRNA levels of ACSS2 were higher in 2-NBDG ^Lo^ and Hoechst ^Lo^ TILs led us to explore the effect of ACSS2 inhibitors on gene expression in these cells. We found that expression of several T cell receptor- and IFN-I-induced cytokines and ISGs was reduced by two independent ACSS2 inhibitors in TILs stimulated ex vivo, showing that this enzyme is functional in TILs. However, our experiments in vivo yielded different, and relatively modest, effects of both inhibitors in TILs. These differences in vivo might involve indirect effects from other cell types in the tumor microenvironment that could be variably sensitive to the inhibitors, or differences in the pharmacokinetics and metabolization of these drugs. In this regard, results in the literature differ as to the role of ACSS2 in TILs. Qiu et al (Qiu et al, 2019) showed that reducing ACSS2 expression by shRNA knockdown in CD8 T cells made them poorer IFNγ producers and weaker tumor controllers in vivo, while Miller et al (Miller et al, 2023) found that ACSS2-deficient immune cells were as competent as wild-type ones in antitumor capacity, and concluded that the overall antitumor effect of the ACSS2 inhibitor VY-3-135 was attributable to the inhibition of ACSS2 in tumor cells, which freed available acetate to be used by TILs. The differing results between Miller et al and Qiu et al about the role of ACSS2 in the antitumor activity of TILs could be due to differences in the degree of inhibition of ACSS2 in different experimental systems, and the balance of acetate (the substrate for ACSS2) versus other metabolites in the TIL microenvironment. Work by the Hess laboratory showed that acetate can downregulate the expression of ACSS2 itself, and have pro- and anti-inflammatory effects, such as enhancing or inhibiting the expression of IFNγ, depending on the activation stage of the T cell (Balmer et al, 2020). In this context, a limitation of our work is that we did not determine intracellular levels of glucose and acetate, and the fate of their metabolites in in vivo effector TILs in tumor regions with poor or high blood accessibility. Future studies characterizing the intracellular metabolite landscape of TILs in poorly perfused tumor regions will help to elucidate their metabolic plasticity and adaptations.

The enhanced expression of signature IFN response genes in both CD4 and CD8 effector TILs with reduced access to blood was intriguing. Elevated IFN responses are characteristic of bystander effector and memory T cells in different tissues and physiopathological situations (Low et al, 2020; Thibaut et al, 2020; Hoekstra et al, 2020). Bystander T cells in tumors may not recognize tumor-specific antigens but exhibit an activated phenotype linked to their responsiveness to locally produced cytokines, and can contribute to antitumor immunity (Meier et al, 2022). As IFN-I can strengthen antitumor T lymphocyte activity (Zitvogel et al, 2015; Katlinski et al, 2017; Duong et al, 2022), a heightened IFN-I response in TILs with limited access to blood could help them retain antitumor capability. As to the mechanisms underlying this response, we found that CD11b ^+^ myeloid cells, but not TILs themselves, in tumor regions with lower uptake of 2-NBDG, and likely reduced blood accessibility, expressed higher levels of IFNβ1 and IFNα mRNA, as well as IFN-I-induced gene products IFIT1, CXCL9 and CXCL10, than myeloid cells in better perfused regions. In addition, TILs in poorly accessible regions expressed moderately higher levels of surface IFNAR1. Our in vitro experiments with lymph node T cells also showed that addition of IFN-I can overcome a glucose-poor environment to induce a higher expression of ISGs (CXCL10, IFIT1, STAT1) than just stimulating the cells in normal glucose but without extra IFN-I. Altogether, our results support the interpretation that myeloid cells in poorly perfused tumor regions are a likely source of IFN-I for TILs. Further research would be needed to identify specific IFN-I-producing myeloid populations, and how their higher expression of IFNβ1 and IFNα is stimulated in tumor microenvironments with reduced blood perfusion.

The elevated expression of the IFN-inducible chemokine CXCL10 in T lymphocytes and myeloid cells in poorly accessible tumor regions could help stimulate CXCR3 ^+^ effector CD8 cytotoxic TILs, as CXCR3 not only drives chemotaxis but also enhances their antitumor capacity in response to anti-PD-1 immunotherapy (Chow et al, 2019; Dangaj et al, 2019). On the other hand, CXCR3 in tumor-infiltrating CD44 ^+^ CD4 Treg cells helps them to interact with CXCL9-expressing type 1 dendritic cells (DC1), preventing DC1 from stimulating antitumor cytotoxic CD8 cells (Moreno Ayala et al, 2023). It remains to be determined whether TILs in less accessible tumor regions are poorer or better responders to CXCR3 ligands than those in well-perfused regions.

Despite that effector TILs in less perfused tumor regions had some features of glucose-restricted T lymphocytes, our results also indicate that they might not be starved of glucose, as we have found that Hoechst ^Lo^ TILs produced comparable amounts of IFNγ and granzyme B as their Hoechst ^Lo^ counterparts shortly after being extracted from the tumors. Our results are in line with work by the Rathmell lab showing that TILs might be able to capture sufficient glucose to maintain their functionality (Reinfeld et al, 2021), and with results with γδ TILs showing that, within the same tumor, IFNγ-producing antitumor cells are markedly glycolytic, whereas IL-17-producing pro-tumor γδ T cells depend on glucose and mitochondrial respiration (Lopes et al, 2021). In this regard, we found that TILs in LLC tumors were better producers of IFNγ than IL-17A, and, even in less perfused regions, were mainly dependent on glucose for energy metabolism. Nonetheless, our metabolic profiling with SCENITH (Argüello et al, 2020) indicates that TILs less accessible to blood subsist in a nutrient-limited microenvironment, as they are metabolically less active than cells with higher accessibility. These results align with our earlier finding that activated T cells would still use glucose as a major substrate for ATP production even in glucose-poor medium. They also suggest that TILs in poorly perfused regions, and likely suboptimal nutrient availability, might resort to lowering their metabolism to persist in their microenvironment, which would allow them to maintain glucose-dependent functions and remain capable of responding readily to T cell receptor stimulation.

We show that poorly perfused TILs expressed lower levels of T regulatory and exhaustion-associated proteins, which suggests that they might be less vulnerable to suppressor mechanisms from the tumor microenvironment. It is possible that effector TILs with lower accessibility to blood might respond to anti-checkpoint immunotherapy differently from more blood-accessible TILs, not only because they express less PD-1, TIM3 or LAG3, but also because they might be less accessible to blood-transported antibodies (Thurber et al, 2008). Also, differences in the proportion of effector and Treg cells between tumor regions with better or poorer access to blood could affect the outcome of anti-PD-1 treatment, as suggested by work showing that the suppressive capacity of human Tregs in glucose-low, lactate-rich media is enhanced by anti-PD-1 treatment (Kumagai et al, 2022). Our results are in line with work by De Ponte Conti et al (De Ponte Conti et al, 2021), who showed that more metabolically active TILs (higher puromycin incorporation) are found in oxygenated tumor regions, while cells in hypoxic regions had lower biosynthetic activity. These findings recall our own with Hoechst ^Hi^ TILs having greater biosynthetic capacity than Hoechst ^Lo^ TILs, as shown by the SCENITH assay. De Ponte Conti et al also showed that CD4 TILs with higher biosynthetic capacity and mTOR activity in vivo contained more FOXP3 ^+^, CTLA4 ^+^ Treg cells, and that CD8 TILs with higher translation capacity also expressed more PD-1 and TIM3 surface proteins (De Ponte Conti et al, 2021). This recalls our finding that TILs with better blood accessibility have higher protein translation activity yet express higher amounts of repressive receptors yet. Results by De Ponte Conti using the B16F10 mouse melanoma tumor model and us here with LLC lung carcinoma are complementary and suggest that TILs in areas better irrigated by blood might face stronger pressure from inhibitory (Treg, immune checkpoints) and exhaustion-inducing mechanisms. It will be important to determine the actual antitumor capacity of TILs extracted from poorly and highly irrigated tumor regions, and whether their distinctive features can be maintained if they are removed from their original microenvironment.

In sum, our work has uncovered different functional and metabolic characteristics of tumor-infiltrating effector T lymphocytes with better or poorer access to blood, and provides evidence supporting the functionality of T cells in a metabolically challenging tumor microenvironment. These results open new paths for exploring the functional heterogeneity and compartmentalization of TILs in different tumor regions. Such knowledge could help devise new ways for therapeutic manipulation of cellular interactions in the tumor microenvironment.

## Methods

**Table:** Reagents and tools table

Reagent/resource	Reference or source	Identifier or catalog number
**Experimental models**		
C57BL/6 J (*M. musculus*)	Charles River	C57BL/6NCrl. Strain Code 027
*Ifnar1* ^−/−^ mice: B6.129S2-Ifnar1tm1Agt/Mmjax	Dr. Manuel Rebelo, Gulbenkian Institute (Lisbon, Portugal)	The Jackson Laboratory, RRID: MMRRC_032045-JAX
LLC cell line (C57BL/6 Lewis lung carcinoma, mouse)	Dr. Ignacio Melero, CIMA, Universidad de Navarra (Pamplona, Spain)	ATCC, CRL-1642
**Antibodies**		
*Antibodies for flow cytometry, immunofluorescence, functional assays, and antibody-based kits (cell isolation beads, ELISA, LEGENDplex, SCENITH)*		
Anti-phospho-S6 (S235/236) antibody	Cell Signaling Technology	Catalog: 2211S
Secondary FITC-labeled antibody (donkey anti-rabbit)	Biolegend	Catalog: 40640
CD45.2 PE-Dazzle 594	Biolegend	Catalog: 109846
CD90.2-PE-Cy7	Biolegend	Catalog: 140310
CD4-PE-Cy5	eBioscience	Catalog: 15-0042-83
CD8-PE	Biolegend	Catalog: 100708
CD8a-Brilliant Violet 785	Biolegend	Catalog: 100749
CD44-APC-Cy7	Biolegend	Catalog: 103028
CD62L-APC	eBioscience	Catalog: 17-0621-83
CD62L-Brilliant Violet 510	Biolegend	Catalog: 104441
IFNAR-1 PE/Cyanine7	Biolegend	Catalog: 127325
IFNAR-1 PE	Biolegend	Catalog: 127311
CD16/32 antibody (clone 93)	eBioscience	Catalog: 14-0161-82
CD11b APC	Biolegend	Catalog: 101212
CD11c Brilliant Violet 785	Biolegend	Catalog: 117336
NK-1.1 PE	Biolegend	Catalog: 156504
CD45R/B220 Alexa Fluor 700	Biolegend	Catalog: 103232
FOXP3 Alexa Fluor 488	Biolegend	Catalog: 126405
FOXP3 PE	eBioscience	Catalog: 12-5773-82
CTLA4 (CD152) PE	Biolegend	Catalog: 106305
PD-1 (CD279) Brilliant Violet 605	Biolegend	Catalog: 135220
TIM-3 (CD366) PE/Fire 810	Biolegend	Catalog: 119745
LAG-3 (CD223) PE/Fire 700	Biolegend	Catalog: 125261
IFNγ PE	BD Pharmingen	Catalog: 554412
Granzyme B PE/Dazzle 594	Biolegend	Catalog: 396427
CD107a (LAMP-1) Brilliant Violet 711	Biolegend	Catalog: 121631
Hamster anti-mouse CD3 clone 145-2C11, for T cell stimulation	BD Biosciences	Catalog: 553058
CD31 (confocal microscopy)	R&D Systems	Catalog: FAB3628G
CD4 (confocal microscopy)	Abcam	Catalog: ab183685
CD8 (confocal microscopy)	Abcam	Catalog: ab217344
Secondary Cy3-labeled antibody (donkey anti-rabbit) (confocal microscopy)	Jackson ImmunoResearch	Catalog: 711-165-152
CD16/32 blockade for confocal microscopy	BioLegend	Catalog: 101302
Hamster anti-mouse CD28 for T cell stimulation	BD Biosciences	Catalog: 553295
Goat anti-hamster IgG, affinity-purified antibody for plate coating	MP Biomedicals	Catalog: 856984
Dynabeads™ Mouse T-activator CD3/CD28	Gibco Thermofisher	Catalog: 11456D
MagnisortTM Mouse CD4 T lymphocyte Enrichment Kit	eBioscience	Catalog: 8804-6821-74
Dynabeads flowcomp mouse pan T	Thermofisher	Catalog: 11465D
Immunomagnetic Dynabeads Mouse CD4 ^+^	Invitrogen	Catalog: 11445D
Rat anti-mouse CD8a hybridoma 53-6.7	Dr. Gabriel Gil. Hospital Mar Research Institute (Barcelona, Spain)	Ledbetter and Herzenberg, 1979
Dynabeads Sheep anti-Rat IgG	Invitrogen	Catalog: 11035
IL-17A Mouse ELISA Ready-Set GO® system	eBioscience	Catalog: 88-7371-88
IL-22 Mouse ELISA Ready-Set GO® system	eBioscience	Catalog: 88-7422-88
IFNγ Mouse ELISA Ready-Set GO® system	eBioscience	Catalog: 88-7314-88
LEGENDplex Mouse T Helper Cytokine Panel (12-plex) Version 3	Biolegend	Catalog: 741043
SCENITH kit KickOff 647	GammaOmics	https://www.scenith.com/
**Chemicals, enzymes and other reagents**		
Glucose-free DMEM	Gibco	Catalog: 11966025
Glucose	Life Technologies	Catalog: A2494001
Fetal bovine serum	Life Technologies	Catalog: 10270-106
Non-essential amino acids	Life Technologies	Catalog: 11140-035
L-glutamine	Life Technologies	Catalog: 25030024
β-mercaptoethanol	Gibco	Catalog: 31350-010
Hepes	Life Technologies	Catalog: 15630-056
Penicillin and streptomycin	Gibco	Catalog: 15140122
Recombinant human IL-2	Proleukin; Chiron, formerly Eurocetus, Emeryville, CA, USA	www.proleukin.com
Recombinant mouse IL-6	ImmunoTools	Catalog: 12340063
Recombinant human TGFβ	PeproTech	Catalog: 100-21
Recombinant mouse IL-12	Peprotech	Catalog: 210-12
Recombinant mouse IFNα4	R&D Systems	Catalog: 12115-1
Recombinant mouse IFN-β1 (carrier-free)	Biolegend	Catalog: 581302
DMSO	Sigma	Catalog: D2650
ACSS2 inhibitor ACSS2i/VY-3-249	MedChemExpress	Catalog: HY-104032; CAS: 508186-14-9
ACSS2 inhibitor VY-3-135	MedChemExpress	Catalog: HY-145953; CAS: 1824637-41-3
Polyethylene glycol 300	MedChemExpress	Catalog: HY-Y0873
Tween 80	MedChemExpress	Catalog: HY-Y1891
2-deoxyglucose (2-DG)	Sigma	Catalog: D8375-5G
Etomoxir	Sigma	Catalog: E1905-25
Epigallocatechin gallate (EGCG)	Sigma	Catalog: E4143
Oligomycin A	Sigma	Catalog: 75351
Sodium azide	Sigma	Catalog: 71289
Collagenase A	Roche	Catalog: 10103578001
DNase I	Sigma-Aldrich	Catalog: D4263-5VL
Erythrocyte lysis buffer	Biolegend	Catalog: 420301
Phosphate buffer saline (PBS, 10x) pH 7.4, sterile	Gibco	Catalog: 70011044
ProLong Gold Antifade Mountain	Thermo Fisher Scientific	Catalog: P36930
Superfrost^TM^ Plus Microscope Slides	Fisherbrand	Catalog: 22-037-246
**Software**		
FlowJo software	FlowJo BD Research	https://www.flowjo.com/flowjo/
FACSDiva versions 6.2 and 8 software	BD biosciences	http://www.bdbiosciences.com/us/instruments/research/software/flow-cytometry-acquisition/bd-facsdiva-software/
Spectroflo 3.3.0 software	Cytek	Release 12282023
SpectroFlo CS version 1.4.0	Cytek	Release 10282024
Sasquatch Software Version 1.19.47880.7 (Bigfoot sorter)	Invitrogen	https://www.thermofisher.com/es/es/home/life-science/cell-analysis/flow-cytometry/flow-cytometers/bigfoot-spectral-cell-sorter/features.html#automation
BD FACSChorus Version 6.1.0 (S8 cell sorter)	BD biosciences	https://www.bdbiosciences.com/en-us/products/software/instrument-software/bd-facschorus-software
Legendplex software Version 2025-05-01	Biolegend	https://www.biolegend.com/en-ie/immunoassays/legendplex/support/software
Microscopy software	FIJI	FIJI/ImageJ software
ZEN Blue software	Zeiss, version 3.x	https://www.zeiss.com/microscopy/en/products/software/zeiss-zen-lite.html
FastQC (version 0.11.5)		Brown et al, 2017
Skewer (version 0.2.2)		Jiang et al, 2014
RiboPicker (version 0.4.3)		Schmieder et al, 2012
STAR (version 2.5.3a)		Dobin et al, 2013
Qualimap (version 2.2.1)		Okonechnikov et al, 2016
Gene Set Enrichment Analysis (GSEA)		Subramanian et al, 2005
GraphPad Prism 6	GraphPad	https://www.graphpad.com/
**Other**		
*Real time quantitative PCR*		
High Pure RNA isolation kit	Roche	Catalog: 11828665001
NanoDrop spectrophotometer	Thermofisher	ND-1000
First Strand cDNA synthesis kit with random hexamers	Roche	Catalog: 04897030001
High-Capacity cDNA Reverse Transcription Kit with RNAse inhibitor	Applied Biosystems	Catalog: 4374967
LightCycler 480 SYBR Green I Master Mix	Roche	Catalog: 4729749001
LightCycler 480 Real-Time PCR System	Roche	Catalog: 05015243001
*Other equipment, labware*		
Confocal microscopy	ZEISS	LSM980 Airyscan 2
Cryostat	Leica Biosystems	CM3050
ELISA plate reader	TECAN	INFINITE 200 Pro
FB 12 Luminometer	Berthold detection systems	FB12-e-07/04
ATP Bioluminescence Assay Kit CLS II	Roche	Catalog: 11699695001
Glucose Assay kit I	Eton Bioscience	Catalog: 12000310
96 well-plates for ELISA	Costar	Catalog: 3590
Cell strainer (Pluristrainer 400 µm)	Pluriselect	Catalog: 43-50400-01
35-μm nylon mesh	Falcon	Catalog: 352235
*Flow cytometry reagents (not antibodies)*		
Fixation/permeabilization solution for intracellular staining	eBioscience	Catalog: 00-5521
Permeabilization buffer (Permwash)	eBioscience	Catalog: 00-8333-56
Foxp3/Transcription Factor Fixation/Permeabilization Concentrate and Diluent kit	eBioscience	Catalog: 00-5523-00
2-(N-(7-nitrobenz-2-oxa-1,3-diazol-4-yl) amino)-2-deoxyglucose (2-NBDG)	Life Technologies	Catalog: N13195
Hoechst 33342	Thermofisher	Catalog: H3570
*Flow cytometers (analyzers and cell sorters)*		
LSRII flow cytometer	BD biosciences	
LSR Fortessa	BD biosciences	
BD FACSAria II cell sorter	BD biosciences	
Aurora 4 L	Cytek	
Aurora 5 L	Cytek	
Aurora CS 5 L	Cytek	
Bigfoot Spectral Cell Sorter	Invitrogen	
BD FACSDiscover S8 cell sorter	BD biosciences	
*RNA sequencing*		
RNeasy Microkit	Qiagen	Catalog: 74004
Smart-Seq2 single-cell protocol		Picelli et al, 2014
SuperScript™ II reverse transcriptase	Invitrogen	Catalog: 18064014
Qubit dsDNA High Sensitivity assay	Invitrogen	Catalog: Q32851
Agilent Bioanalyzer	Agilent	Catalog: 5067-4626
Fragment analyzer High Sensitivity assay	Agilent	Catalog: DNF-474
NEBNext® Ultra DNA Library Prep for Illumina® kit	New England Biolabs (NEB)	Catalog: E7370
AgenCourt AMPure XP beads	Beckman Coulter	Catalog: A63882
NEBNext® Multiplex Oligos for Illumina	New England Biolabs (NEB)	Index Primers Set 1, Catalog: E7335), Index Primers Set 2, Catalog: E7500, Index Primers Set 3, Catalog: E7710, Index Primers Set 4, Catalog: E7730
KAPA Library Quantification Kit	KapaBiosystems	Catalog: KK4835
Illumina cBot	Illumina	https://support.illumina.com/downloads/cbot_user_guide_15006165.html
Illumina HiSeq 2500	Illumina	https://support.illumina.com/sequencing/sequencing_instruments/hiseq_2500/documentation.html
**Primers used for mRNA analysis by real-time quantitative PCR (RT-qPCR)**		
*Gene product*	*Forward primer (5’-3’)*	*Reverse primer (5’-3’)*
B2m (beta-2 microglobulin)	TTCTGGTGCTTGTCTCACTG	GGAACTGTGTTACGTAGCAG
IL-17A	TCAGACTACCTCAACCGTTC	AATTCATGTGGTGGTCCAGC
IFNγ	CTCAAGTGGCATAGATGTGG	CAGGTGTGATTCAATGACGC
IL-22	CCTGATGAAGCAGGTGCTAA	TCCTTCAGCCTTCTGACATTCTT
L32	ACCAGTCAGACCGATATGTG	ATTGTGGACCAGGAACTTGC
FOXP3	CAAGGGCTCAGAACTTCTAG	AGCTGATGCATGAAGTGTGG
CTLA4	TCTGAAGCCATACAGGTGACC	TGGTCATTTGTCTGCCGC
IFNβ1	AGCTCCAAGAAAGGACGAAC	TCTGGAGCATCTCTTGGATG
Pan-IFNα	CCTGCTGGCTGTGAGGA	GGAAGACAGGGCTCTCCAG
CXCL9	ATCTTCCTGGAGCAGTGTGG	GGCAGGTTTGATCTCCGTTC
CXCL10	AAGGGATCCCTCTCGCAAGGAC	ATCGTGGCAATGATCTCAACAC
CCL5	TTGTCACTCGAAGGAACCGC	AGAGCAAGCAATGACAGGGA
STAT1	TGGTGAAATTGCAAGAGCTG	CAGACTTCCGTTGGTGGATT
IFIT1	TACAGGCTGGAGTGTGCTGAGA	CTCCACTTTCAGAGCCTTCGCA
CXCR3	TGTACCTTGAGGTTAGTGAACG	GGAGTCAGAGAAGTCGCTCT
ACSS2	TCTGCTACAACGTGCTGGAT	CACCCTTCTGAATGCCCTGT

### Mice

C57BL/6 wild-type mice were bred and housed in specific pathogen-free conditions at the animal facility of Parc de Recerca Biomèdica de Barcelona (PRBB). *Ifnar1* ^−/−^ mice, described in (Huerga Encabo et al, 2020), were kept in a C57BL/6 background and were obtained from Manuel Rebelo at the Rodent Facility of the Gulbenkian Institute (Lisbon, Portugal). Male and female animals were used, and similar findings were obtained for both sexes.

### T lymphocyte isolation and culture

Naive CD4 T cells (CD62L ^+^ CD44 ^neg^) were FACS-sorted from a pool of spleen and lymph nodes and rested overnight in T cell medium (glucose-free DMEM, 10% fetal bovine serum (FBS), non-essential amino acids, 2 mM L-glutamine, 50 μM β-mercaptoethanol, 10 mM Hepes, and penicillin and streptomycin). Glucose (5 mM) and recombinant human IL-2 (5 ng/ml) were added during the overnight incubation. CD4 T cells were then stimulated by coating them with hamster anti-mouse CD3 (1 μg/10^6^ cells) plus hamster anti-mouse CD28 (1 μg/10^6^ cells) antibodies (for 1 h at 4 °C, and adding them to plates bound with goat anti-hamster IgG (9.5 μg/cm^2^). Cultures were done in flat-bottomed 96-well plates, 120 × 10^3^ cells per well in 150 μl, in low (0.3 mM) or normal (5 mM) glucose under Th17 polarizing conditions with 10 ng/ml IL-6 and 2.5 ng/ml TGFβ. In the low glucose condition, its only source was provided by the 10% FBS, and was measured to be 0.3 mM. After a 48-h stimulation, cells were harvested for RNA extraction and supernatants were saved frozen at −80 °C for later cytokine analysis with a Legendplex bead array. For cultures of fresh mouse CD4^+^ T lymphocytes, they were obtained from lymph nodes and spleen with the MagnisortTM Mouse CD4 T lymphocyte Enrichment Kit according to the manufacturer’s instructions. Cells (1 to 1.5 × 10^6^ cells/ml) were activated as Th0 with anti-mouse CD3 (1 μg/10^6^ cells) plus anti-mouse CD28 (1 μg/10^6^ cells) antibodies in plates coated with goat anti-hamster IgG (9.5 μg/cm^2^) in T cell medium without glucose (only 0.3 mM final glucose was provided by the 10% FBS) or with glucose supplementation as indicated in the respective figures. Th0 cultures also had 5 ng/ml recombinant human IL-2. After 48 h and depending on visual estimation of cell density, cells were split 1/2 or 1/3 in fresh medium supplemented with 5 ng/ml IL-2. For expansion of preactivated CD4 T lymphocytes under Th17 polarization, Th0 cells were restimulated at 10^6^ cells/ml with anti-CD3 and anti-CD28 antibodies as above, plus 10 ng/ml IL-6 and 2.5 ng/ml TGFβ for up to 4 days. For Th1 polarization T lymphocytes were cultured with 5 ng/ml of recombinant murine IL-12. For stimulation of preactivated T lymphocytes with type I IFN, T lymphocytes, both CD4 and CD8 isolated from lymph nodes with Dynabeads flowcomp mouse pan T, were first activated as Th0 with anti-CD3 and anti-CD28 plus IL-2 as indicated for CD4 cells above. After 5 days, T lymphocytes were harvested, adjusted at 10^6^ cells/ml in fresh medium (15 mM or 0.3 mM glucose) and restimulated with anti-CD3 and anti-CD28 antibodies plus IL-2 (5 ng/ml) for 24 h without or with additional stimulation with a cocktail of 600 U/ml recombinant mouse IFN alpha 4 (IFNα4) and 2.5 ng/ml recombinant mouse IFN-β1 (IFNβ1). Cells were then isolated as CD4 or CD8 respectively with immunomagnetic beads (Dynabeads Mouse CD4 ^+^, or Dynabeads Sheep anti-Rat IgG, coated with anti-mouse CD8 rat hybridoma 53-6.7). FACS-sorted effector CD4 TILs (CD62L ^neg^ CD44 ^+^) were used in Figs. 3A,  EV2B, and  7. TIL sorting is described later in “Flow cytometry and cell sorting of tumor-infiltrating T lymphocytes”. For Figs. 3 and EV2B, sorted CD4 effector TILs were rested overnight in 5 mM glucose T cell medium plus IL-2 (5 ng/ml). The next day they were washed and activated as Th0 with Dynabeads™ Mouse T-activator CD3/CD28 plus IL-2, in 0.3 or 5 mM glucose medium for 48 h. For Fig. 7, sorted cells were rested as above and the next day stimulated with Dynabeads™ Mouse T-activator CD3/CD28 in 100 μl with 600 U/ml IFNα4 and 2.5 ng/ml IFNβ1 for 24 h. DMSO (solvent, 0.02%) or ACSS2 inhibitors (10 μM) were added 10 min before the stimulation. Cells were harvested for RNA extraction and supernatants were saved frozen for later cytokine analysis with Legendplex.

### Gene expression analysis

Total RNA from T lymphocytes and other cell populations was isolated using the High Pure RNA isolation kit (Roche), quantified in a NanoDrop (ND-1000) spectrophotometer and 100 ng to 300 ng of total RNA was retrotranscribed to cDNA using the First Strand cDNA synthesis kit with random hexamers (Roche), or the High-Capacity cDNA Reverse Transcription Kit with RNAse inhibitor (Applied Biosystems). For the analysis of smaller numbers of sorted TILs we used Qiagen’s RNeasy Micro Kit and usually all of the RNA was retrotranscribed. Gene expression was analyzed by real-time quantitative PCR (RT-qPCR) using LightCycler 480 SYBR Green I Master Mix (Roche) and the LightCycler 480 Real-Time PCR System (Roche) according to the manufacturer’s instructions. Primers used for RT-qPCR are listed in the Reagents and Tools table.

### LEGENDplex cytokine analysis by flow cytometry

The LEGENDplex Mouse T Helper Cytokine Panel (12-plex) Version 3 (Biolegend) was used following the manufacturer’s instructions. Tests with several supernatants were done to determine the optimal sample dilutions: TIL culture supernatants were diluted 1/10 in assay buffer, and supernatants from naive CD4 T cells were undiluted. Flow cytometry data were acquired with Aurora 5 L flow cytometer, and analysis of the data was done with the Legendplex software Version 2025-05-01.

### Enzyme-linked immunosorbent assay (ELISA)

Cell-free supernatants from cultures of isolated CD4^+^ T lymphocytes (1  × 10^6^ cells/ml) were harvested and stored at −80 °C. Detection of IL-17A, IL-22 and IFNγ was done in duplicate in 96 well-plates (Costar) with the Mouse ELISA Ready-Set GO® systems (eBioscience) following the manufacturer’s instructions.

### ATP measurement

At different times upon culture in 5 or 0.3 mM glucose medium, T lymphocytes were adjusted at 50,000 cells/ml in their own culture medium and 0.5 ml of the cell suspension were transferred to 1.5-ml microcentrifuge tubes and incubated at 37 °C for 2 h in the absence or presence of metabolic inhibitors: 1 mM to 50 mM 2-deoxyglucose (2-DG), 200 µM etomoxir, 50 µM epigallocatechin gallate (EGCG), 0.1 µg/ml oligomycin and 20 mM sodium azide. Cells were harvested, washed with a solution of 150 mM NaCl and 50 mM Tris pH 8, resuspended in 100 µl of the same wash solution plus 0.2% Triton X-100 and frozen at −80 °C. ATP concentration in cell lysates was measured in a FB 12 Luminometer (Berthold detection systems) using the ATP Bioluminescence Assay Kit CLS II (Roche).

### Glucose measurement

Cell-free supernatants from cultures of isolated CD4 ^+^ T lymphocytes (1 × 10^6^ cells/ml) were harvested and stored at −80 °C. Glucose measurements were done in duplicate in 96 well-plates (Costar) using the Glucose Assay kit I (Eton Bioscience) as in the manufacturer’s instructions.

### In vivo anti-CD3 injection and ex vivo restimulation

In all, 8–12-week-old C57BL/6 female mice were injected intraperitoneally with anti-CD3 1 mg/kg (clone 145-2C11, DB Bioscience) to induce systemic inflammation and T lymphocyte activation (Esplugues et al, 2011; Alberdi et al, 2017). After 48 h, CD4^+^ T lymphocytes were isolated from peripheral lymph nodes, and restimulated ex vivo with anti-CD3 plus anti-CD28 (1 µg/10^6^ cells) in plates coated with goat anti-hamster antibody, with the glucose concentration and polarizing conditions indicated in Fig. EV1C.

### Intracellular staining for phospho-S6 and flow cytometry

T lymphocytes were fixed with a fixation/permeabilization solution (eBioscience, catalog: 00-5521) during 40 min to 1 h at 10^5^ cells/100 µl. They were then washed twice with permeabilization buffer (Permwash, eBioscience) and permeabilized for 20 min in the same solution. Anti-phospho-S6 (S235/236) antibody (Cell Signaling Technology) was then added at a 1:100 dilution and incubated for 2 h at 4 °C. After washing cells twice with permwash solution, cells were incubated with a secondary FITC-labeled antibody (donkey anti-rabbit) for 20 min at 4 °C. Cells were then washed twice, resuspended in PBS and analyzed with an LSR II flow cytometer. Data analysis was done using the FlowJo software (TreeStar).

### Lewis lung carcinoma (LLC) tumors, in vivo labeling with 2-NBDG and Hoechst 33342

LLC cells derived from C57BL/6 mice were kindly provided by Dr. I. Melero (Center for Applied Medical Research, Pamplona, Spain). LLC cells were grown in complete medium and maintained at subconfluency by passing them with gentle pipetting. LLC were routinely tested and confirmed to be mycoplasma-free. For solid tumor development, 5 × 10^5^ LLC cells per mouse were injected subcutaneously in the right back flank of 8 to 12-week-old male or female C57BL/6 mice. Starting at day 7 post-inoculation, tumors were measured every other day using a caliper and the tumor volume was calculated using the formula L × W^2^ × 0.52, where L = maximal length and W = maximal width. Only one tumor was implanted per mouse. All mice were euthanized at the same endpoint in each experiment, which was between days 12 to 15 after tumor inoculation, when tumors reached ~500 mm^3^ on average.

In vivo labeling of tumor-infiltrating T lymphocytes (TILs) with 2-(N-(7-nitrobenz-2-oxa-1,3-diazol-4-yl) amino)-2-deoxyglucose (2-NBDG, Life Technologies, catalog: N13195) was done by intravenous (via retroorbital route) injection of 2-NBDG (20 mg/kg of mouse body weight; 3.33 mg/ml in PBS). 2-NBDG was allowed to circulate for 10 min before euthanizing the mice and excising the tumors to isolate TILs. Labeling with Hoechst 33342 (Thermofisher; 12 mg/kg of mouse body weight; 2.7 mg/ml in PBS) was done in the same way.

Excised LLC tumors were minced in 1.5 ml tubes containing 0.5 ml complete DMEM without glutamine and β-mercaptoethanol. The content was transferred to a 50 ml tube containing 2.5 ml of the same medium to which 0.5 mg/ml of collagenase A (Roche) and 0.01% DNase I (Sigma-Aldrich) were added. Tumors were digested for 45 min at 37 °C under continuous rotation, filtered through 70-μm cell strainers to remove undigested fragments, and transferred to 15-ml tubes. Cells were then centrifuged (350×*g* for 5 min), incubated with 1 ml erythrocyte lysis buffer (Biolegend) for 7 min at 4 °C, then washed with 10 ml PSA buffer (PBS with 10% FBS and 0.1% sodium azide) and centrifuged again. Tumor pellets were resuspended in PSA to obtain single-cell tumor suspensions for flow cytometry staining or sorting.

### Immunofluorescence staining of frozen LLC tumor sections

Tumor tissues were collected and embedded in optimal cutting temperature (OCT) compound following snap-freezing. Prior to tissue collection, mice had been intravenously perfused with Hoechst dye as described elsewhere. Frozen OCT blocks were sectioned at 8 µm thickness using a Leica CM3050 S cryostat (Leica Biosystems) and mounted on glass slides (Fisherbrand™ Superfrost™ Plus Microscope Slides).

After sectioning, slides were allowed to air dry at room temperature for approximately 30 min prior to fixation. Tissue sections were fixed with 4% paraformaldehyde (PFA) in PBS for 10 min at room temperature, followed by three washes in PBS. Sections were then permeabilized with 0.1% Triton X-100 in PBS for 5–10 min and blocked for 1 h at room temperature in PBS containing Triton X-100, bovine serum albumin (BSA), and donkey serum. To reduce non-specific antibody binding mediated by Fcγ receptors, anti-CD16/CD32 antibody (BioLegend) was included during the blocking step.

Primary antibodies were incubated overnight at 4 °C. The following antibodies were used: anti-CD31 (goat polyclonal IgG, AF488-conjugated, R&D Systems), anti-CD4 (rabbit, Abcam, ab183685, clone EPR19514, 1:50), and anti-CD8a (rabbit, Abcam, ab217344, clone EPR21769, 1:50). For CD4 and CD8 staining, a donkey anti-rabbit Cy3 secondary antibody (Jackson ImmunoResearch, 1:500) was applied for 1 h at room temperature. Sections were mounted using ProLong Gold Antifade Mountant (Thermo Fisher Scientific) and stored at room temperature until imaging.

Because Hoechst was administered in vivo prior to tissue collection, preservation of the dye distribution within the tissue was time-sensitive. Therefore, staining and imaging were performed rapidly after sectioning, and image acquisition was completed within 24 h to minimize diffusion of the Hoechst signal within the tissue.

### Confocal microscopy

Image acquisition was performed using a ZEISS LSM980 Airyscan 2 confocal microscope equipped with 20×/0.8 NA and 63×/1.4 NA oil immersion objectives, GaAsP detectors (for AF488 and Cy3 channels) and Airyscan detection (for Hoechst). Data were acquired using ZEN Blue software (Zeiss, version 3.x).

Fluorophores were excited using the following laser lines: 405 nm (Hoechst), 488 nm (AF488), and 561 nm (Cy3/AF546). Typical acquisition settings were: laser power 1.5% and gain 650 for Hoechst, 2% and 620 for AF488, and 1.5% and 680 for Cy3. Images were acquired at a resolution of 2048 × 2048 pixels, with a pixel size of ~0.3 µm, 8-bit depth, 2× digital zoom, and tile scan mode. Scan speed was set to 5. Imaging was performed at room temperature.

Images were processed and analyzed using FIJI/ImageJ. Linear adjustments of brightness and contrast were applied uniformly across the entire image for each channel to improve visualization (Hoechst: 10–50; AF488: 5–150; Cy3: 10–50). No non-linear transformations (e.g., gamma correction), pseudocoloring, or selective manipulations were applied. Relative fluorescence intensities were preserved across samples.

### Quantification of nuclear Hoechst intensity relative to vascular distance

To assess the relationship between tissue perfusion and T cell localization, Hoechst fluorescence intensity was quantified in individual T cells relative to their distance from blood vessels. CD4⁺ or CD8⁺ T cells were first identified manually in a blinded manner based on immunofluorescence staining. For each cell, the distance to the nearest CD31⁺ blood vessel was determined by measuring the shortest linear vector from the center of the T cell nucleus to the closest CD31-positive vascular structure. A region of interest (ROI) was then manually drawn around the nucleus of each identified T cell using the Hoechst channel. The mean fluorescence intensity (MFI) of Hoechst within each nuclear ROI was quantified using FIJI/ImageJ. These measurements were compiled to generate correlation plots comparing distance from the nearest CD31⁺ vessel and nuclear Hoechst MFI for individual T cells, providing a quantitative assessment of the relationship between vascular proximity and dye perfusion within the tumor tissue.

### Flow cytometry and cell sorting of tumor-infiltrating T lymphocytes

Throughout this study, different flow cytometers were used: LSRII cytometer (BD Biosciences) equipped with 355, 405, 488, and 633 nm lasers, LSR Fortessa (BD Biosciences) equipped with 405, 488, 561, and 633 nm lasers, Aurora 4 L, Aurora 5 L, and Aurora CS 5 L (all three Cytek’s). FACSDiva versions 6.2 and 8 software (BD Biosciences), SpectroFlo 3.3.0 (Aurora 4 L and 5 L), and SpectroFlo CS version 1.4.0 (Aurora CS 5 L) software were used. For cell sorting, cell suspensions were filtered through a 35-μm nylon mesh (Falcon) and isolated with a BD FACSAria II (BD biosciences) cell sorter equipped with 355, 405, 488, 561, and 633 nm lasers, with FACSDiva 6.2 and 8 analysis software, a Bigfoot Spectral Cell Sorter (Invitrogen) with Sasquatch analysis software version 1.19.47880.7, and a BD FACSDiscover S8 cell sorter (BD biosciences) with analysis software BD FACSChorus version 6.1.0.

Effector CD4 and CD8 TILs were identified as CD44 ^+^ CD62L ^neg^ within alive T lymphocytes cells gated as CD45.2 ^+^, CD90.2 ^+^ (Thy1.2), and CD4 ^+^ or CD8 ^+^ respectively. The antibodies used were: CD45.2 PE-Dazzle 594 (Biolegend), CD90.2-PE-Cy7 (Biolegend), CD4-PE-Cy5 (eBioscience), CD8-PE (Biolegend) CD8a-Brilliant Violet 785 ™ (Biolegend), CD44-APC-Cy7 (Biolegend) CD62L-APC (eBioscience), CD62L-Brilliant Violet 510™ (Biolegend). Other antibodies used for TIL characterization were: unconjugated anti-CD16/32 antibody (clone 93) for blocking Fcγ receptors (eBioscience), IFNAR-1 PE/Cyanine7 (Biolegend), IFNAR-1 PE (Biolegend). Other immune cell populations (CD45.2 ^+^) in the LLC tumors were identified with CD11b APC (Biolegend) and CD11c Brilliant Violet 785 (Biolegend) for myeloid cells, NK-1.1 PE (Biolegend) for NK and NKT cells, and CD45R/B220 Alexa Fluor 700 (Biolegend) for B cells.

Antibodies to assess Treg, immune checkpoint and exhaustion-associated markers were:

FOXP3 Alexa Fluor® 488 (Biolegend), FOXP3 PE (eBioscience), CTLA4 (CD152) PE (Biolegend), PD-1 (CD279) Brilliant Violet 605 (Biolegend), TIM-3 (CD366) PE/Fire™ 810 (Biolegend), LAG-3 (CD223) PE/Fire™ 700 (Biolegend). PD-1, TIM3 and LAG3 were analyzed as surface proteins, and FOXP3 and CTLA4 as intracellular proteins, using the Foxp3/Transcription Factor Fixation/Permeabilization Concentrate and Diluent kit, eBioscience. After FcγR blocking and staining of surface markers, cells were fixed for 40 min on ice, then permeabilized 30 min on ice, and stained for intracellular proteins 1 h on ice.

Analysis of intracellular IFNγ, granzyme B, and surface CD107a in ex vivo-stimulated TILs used the same protocol for cell preparation as in the SCENITH assay described below. Cells were immediately placed in medium with 5 mM glucose, and either left unstimulated, stimulated with PMA (20 ng/ml) and ionomycin (1 µg/ml) (P + I) for 4 h, or stimulated with (P + I) for 4 h, with brefeldin A (BFA, 1 µg/ml) added since the beginning of the stimulation to ensure maximal accumulation of the cytokine. After stimulation, cells were washed with cold PBS, resuspended in DMEM, and cell clusters were further digested with 0.5 mg/ml of collagenase A (Roche) plus 0.01% DNase I (Sigma-Aldrich) at 37 °C under continuous rotation for 30 min to obtain single-cell suspensions. BFA was also added to the P + I + BFA samples during this incubation. Effector TILs were identified with antibodies to CD90.2, CD4, CD8, CD44, CD62L; and their expression of IFNγ, granzyme B, and CD107a was analyzed with: IFNγ PE (BD Pharmingen), granzyme B PE/Dazzle™ 594 (Biolegend), and CD107a (LAMP-1) Brilliant Violet 711™ (Biolegend). IFNγ, and granzyme B were detected in permeabilized cells as described above for intracellular FOXP3 and CTLA4. For surface CD107a, the antibody was present during the duration of the assay to enhance detection of the antigen.

Cell sorting of 2-NBDG and Hoechst 33342 high or low populations was done on the 15% of CD4 and CD8 effector cells with the highest or lowest staining with each respective dye. Gating strategies are illustrated in Figs. 3B,  4B,  5A,  6A, and  EV2A,C. Further details about antibodies combinations used in each of the different types of experiments are available from the authors.

### SCENITH assay and metabolic profiling of TILs by flow cytometry

This assay measures the metabolic proficiency of the cells and their dependence on different ATP sources (glycolysis, mitochondrial respiration), by their rate of puromycin incorporation, which in T cells, TILs, and other immune cells is proportional to the ATP content of the cells and a measure of protein translation and biosynthetic capacity (Argüello et al, 2020; Lopes et al, 2021). The SCENITH kit KickOff 647 (GammaOmics; https://www.scenith.com/) was used following the original description of the methodology (Argüello et al, 2020). Briefly, 0.2–0.4 g of tumor were excised from mice that had received a retroorbital injection of Hoechst 33342 10 min before being euthanized. Tumor pieces were minced with scissors and passed through a 400 µm cell strainer (Pluristrainer 400 µm). Each tumor suspension was split in 5 aliquots, and plated in T cell medium with 5 mM glucose in 100 µl at 37 °C, in round-bottom, 96-well plates. Each of the five wells received either no inhibitor (control), the glycolysis inhibitor 2-deoxyglucose (2DG, 100 mM), the mitochondrial respiration inhibitor oligomycin A (1 µM), 2DG + oligomycin A, or harringtonine (2 µg/ml), a translation inhibitor to determine the background puromycin incorporation without active translation. Ten minutes after the inhibitors, puromycin (20 µg/ml) was added without washing out the inhibitors, and incubated for 30 more minutes. After this incubation cells were washed with cold PBS, resuspended in DMEM and cell clusters were further digested with 0.5 mg/ml of collagenase A (Roche) plus 0.01% DNase I (Sigma-Aldrich) at 37 °C under continuous rotation for 45 min to obtain single-cell suspensions, followed by blockade of FcγR, staining of surface antigens, and fixation/permeabilization for staining puromycylated nascent peptide chains with anti-puromycin AF647 antibody (included in the SCENITH kit KickOff 647) by flow cytometry.

### RNA sequencing and bioinformatics analysis

Effector CD4 or CD8 TILs from LLC tumor-bearing mice were sorted as 2-NBDG ^Hi^ or 2-NBDG ^Lo^ from 4 independent mice. The 15% of effector TILs with the highest 2-NBDG staining were sorted as 2-NBDG ^Hi^ cells, and the lowest 15% were sorted as 2-NBDG ^Lo^. For each population, a pool of 2000 cells (500 cells per mouse) was done. RNA was extracted with the RNeasy Microkit (Qiagen, Catalog: 74004), and cDNA libraries were prepared from poly A RNA (mRNA) using the Smart-Seq2 single-cell protocol (Picelli et al, 2014) with some modifications. Briefly, reverse transcription was done with SuperScript™ II reverse transcriptase (Invitrogen, Catalog: 18064014), oligo-dT30VN (1 µM; 5′-AAGCAGTGGTATCAACGCAGAGTACT30VN-3′), template-switching oligonucleotides (1 µM) and betaine (1 M). Template switching was done using a locked nucleic acid (LNA) without the purification step before preamplification PCR to obtain an increased cDNA yield. cDNA concentration was measured with the Qubit dsDNA High Sensitivity assay (Invitrogen, Catalog: Q32851) and analyzed with Agilent Bioanalyzer or Fragment analyzer High Sensitivity assay (Agilent, Catalog: 5067-4626 or DNF-474) to check the size distribution profile. cDNA libraries were prepared using NEBNext® Ultra DNA Library Prep for Illumina® kit (NEB, catalog: E7370) according to the manufacturer’s protocol. 5 ng of cDNA were fragmented at a range size of 200–500 bp using Covaris S2, then subjected to end repair and addition of “A” bases to 3′ ends, ligation of adapters and USER excision. All purification steps were performed using AgenCourt AMPure XP beads (Beckman Coulter, catalog: A63882,). Library amplification was performed by PCR using NEBNext® Multiplex Oligos for Illumina (Index Primers Set 1, Catalog E7335), (Index Primers Set 2, Catalog: E7500), (Index Primers Set 3, Catalog E7710) or/and (Index Primers Set 4, Catalog: E7730). Final libraries were analyzed with Agilent Bioanalyzer or Fragment analyzer High Sensitivity assay to estimate the quantity and check size distribution, and were then quantified by qPCR using the KAPA Library Quantification Kit (KapaBiosystems, Catalog: KK4835,) prior to amplification with Illumina’s cBot. Libraries were sequenced 1 ×50 + 8 bp on Illumina’s HiSeq 2500.

The quality of the raw data was checked using FastQC (version 0.11.5) (Brown et al, 2017) and trimmed using Skewer (version 0.2.2) (Jiang et al, 2014). The percentage of reads mapping to ribosomal RNA was assessed using riboPicker (version 0.4.3) (Schmieder et al, 2012). RNA-seq raw reads (31–36 × 10^6^ reads/sample) were mapped to the *Mus musculus* reference genome (Gencode M21 release; mm10; files GRCm38.primary_assembly.genome.fa.gz and gencode.vM21.annotation.gtf) and counted at the gene level using STAR (version 2.5.3a) (Dobin et al, 2013) and parameters ‘--outSAMunmapped None --outSAMtype BAM SortedByCoordinate --quantMode GeneCounts’. As the library preparation protocol was reverse stranded, the 4th column of the STAR gene counts output of each sample was taken as input for DESeq2. Qualimap (version 2.2.1) (Okonechnikov et al, 2016) was used to check the quality of the aligned reads. Pre-Ranked Gene Set Enrichment Analysis (GSEA) (Subramanian et al, 2005) was used to obtain the functional annotation of gene lists, using the Molecular Signatures Database (MSigDB) collections Hallmark and Immunological signature gene sets. Genes with less than 30 reads across all samples were filtered out before processing the data. The accession number for the RNA-seq datasets is GSE250248.

### In vivo treatment with ACSS2 inhibitors ACSS2i (VY-3-249) and VY-3-135

For experiments with the ACSS2i (VY-3-249/HY-104032, MedChemExpress), mice bearing LLC subcutaneous tumors were injected intraperitoneally with 300 µl of ACSS2i (25 mg/kg body weight) prepared in vehicle (10% DMSO, 40% PEG 300, 5% Tween 80, 45% saline) as per manufacturer’s instructions (https://www.medchemexpress.com/Ac-CoA_Synthase_Inhibitor1.html). ACSS2i was injected starting at day 7 or 9 after tumor inoculation, daily during 5 days (Li et al, 2021). Mice were euthanized 16 h after the last administration of vehicle or ACSS2i/VY-3-249. For the experiment with the ACSS2 inhibitor VY-3-135 (MedChemExpress) (Miller et al, 2023), LLC-bearing mice were injected intraperitoneally with 300 µl of ACSS2 inhibitor (100 mg/kg body weight) or vehicle (10% DMSO, 40% PEG 300, 5% Tween 80, 45% saline), at days 10, 12 and 13 after tumor inoculation. Mice were euthanized 16 h after the last administration of vehicle or VY-3-135 inhibitor. In all experiments, mice were injected intravenously (retroorbital) with Hoechst 33342 10 min before euthanasia, to label TILs with higher or lower proximity to blood vessels.

### Statistical analysis

Statistical analyses were done with GraphPad Prism 6. Significance of the differences between sets of experimental data was determined with an unpaired Student’s *t* test, with Welch’s correction when variances were significantly different between the compared groups. An ANOVA test was used as indicated in figure legends. A one-sample *t* test was used when samples were compared to a reference control sample. Paired *t* tests were used to compare 2-NBDG-high versus -low and Hoechst 33342-high versus -low cells, sorted from the same tumor, and when comparing different conditions of the same culture tested simultaneously.

### Study approval

Animal handling and experiments were in accordance with approved protocols by the Parc de Recerca Biomèdica de Barcelona Animal Care and Use Ethics Committee and carried out in accordance with the Declaration of Helsinki and the European Communities Council Directive (86/069/EEC). The registry numbers of the procedures used are CLR-23-0026 and CLR-23-0028.

## Supplementary information

Peer Review File

Source data Fig. 1

Source data Fig. 2

Source data Fig. 3

Source data Fig. 4

Source data Fig. 5

Source data Fig. 6

Source data Fig. 7

Source data Fig. 8

Figures EV1 to EV5 Source data

Expanded View Figures

## Data Availability

RNA sequencing datasets of in vivo labeled, FACS-sorted 2-NBDG ^Hi^ and ^Lo^ CD4 and CD8 effector T cells isolated from tumors have been uploaded to GEO under the accession number GSE250248. Links to the externally deposited source data: fcs flow cytometry files in Fig. 5A,B are deposited in BioStudies: https://www.ebi.ac.uk/biostudies/studies/S-BSST2873; and flow cytometry files in Fig EV2A,C in BioStudies: https://www.ebi.ac.uk/biostudies/studies/S-BSST2210. Confocal images in Figs. 5C and EV3A are deposited in BioStudies: https://www.ebi.ac.uk/biostudies/studies/S-BIAD3159. The source data of this paper are collected in the following database record: biostudies:S-SCDT-10_1038-S44319-026-00799-0.
