# Supplementary figures and images for: Poorly perfused tumor regions harbor T cells with a glucose-dependent effector phenotype
Source: EMBO Rep. 2026 May 27;27(12):3454–87. doi: 10.1038/s44319-026-00799-0 (PMC13303879; doi:10.1038/s44319-026-00799-0)

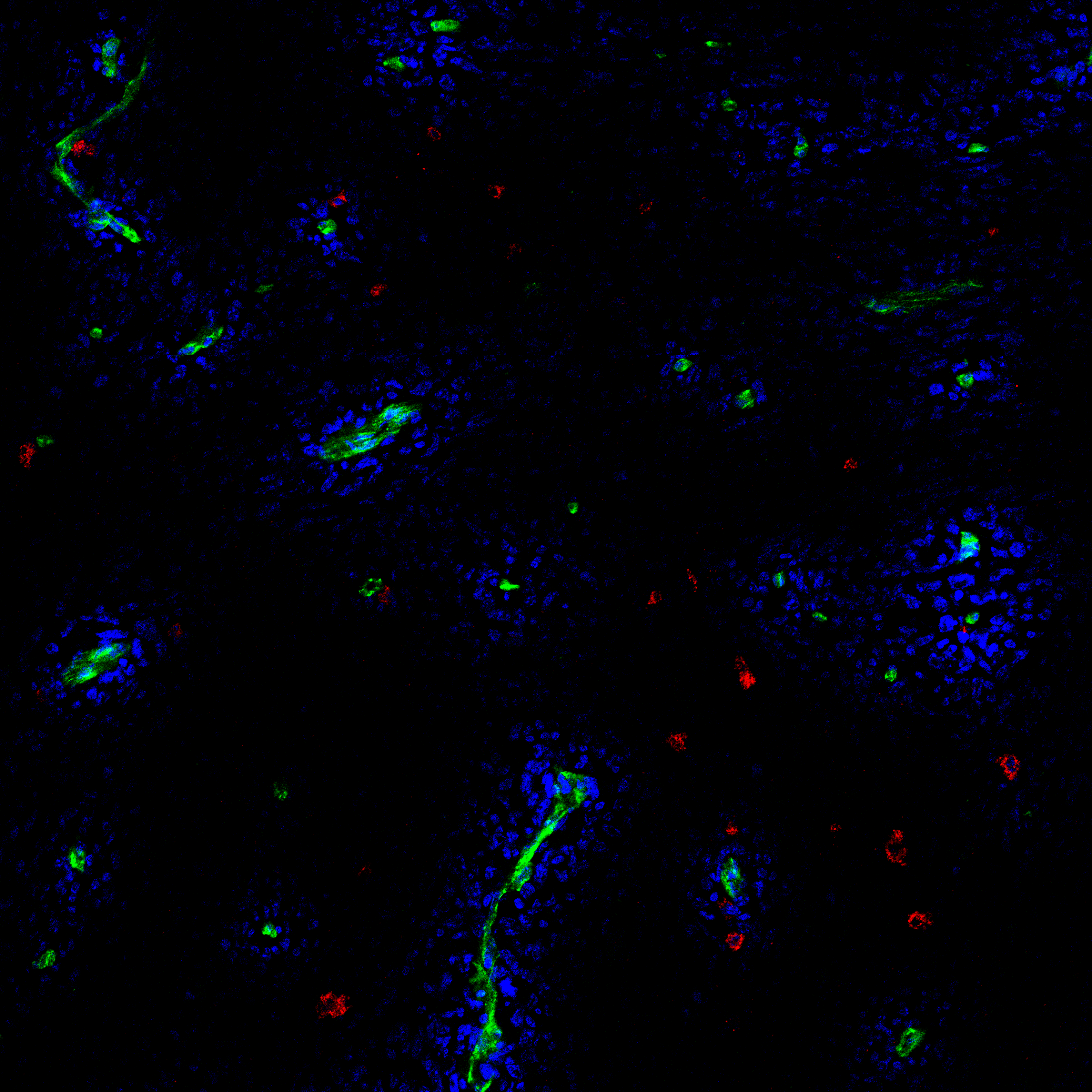

Supplement: Supplementary file 6 — Source data Fig. 5 [file 44319_2026_799_MOESM6_ESM.zip › SourceData Fig5/Fig 5C_.png images/Figure 5 C 1.png]

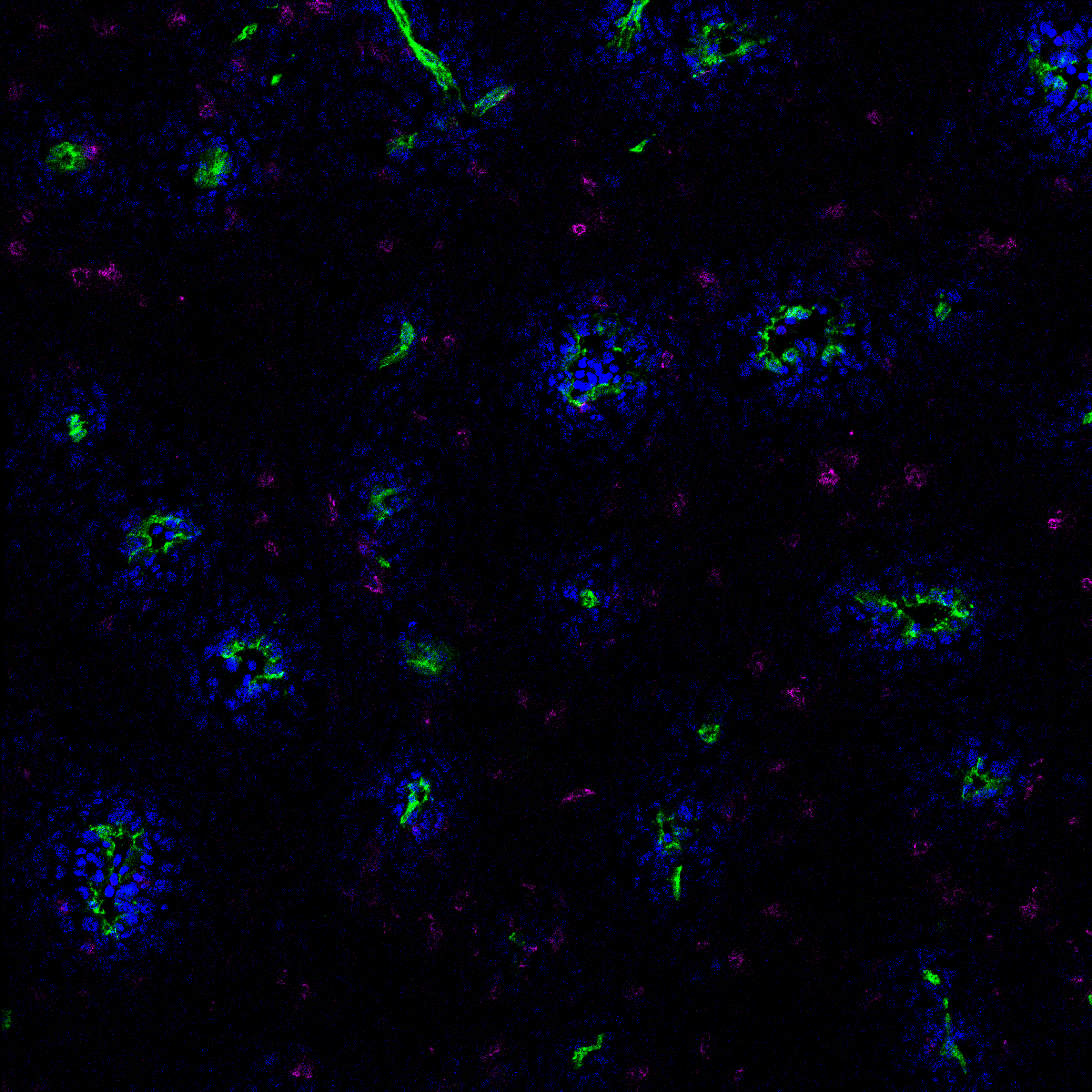

Supplement: Supplementary file 6 — Source data Fig. 5 [file 44319_2026_799_MOESM6_ESM.zip › SourceData Fig5/Fig 5C_.png images/Figure 5 C 3.png]

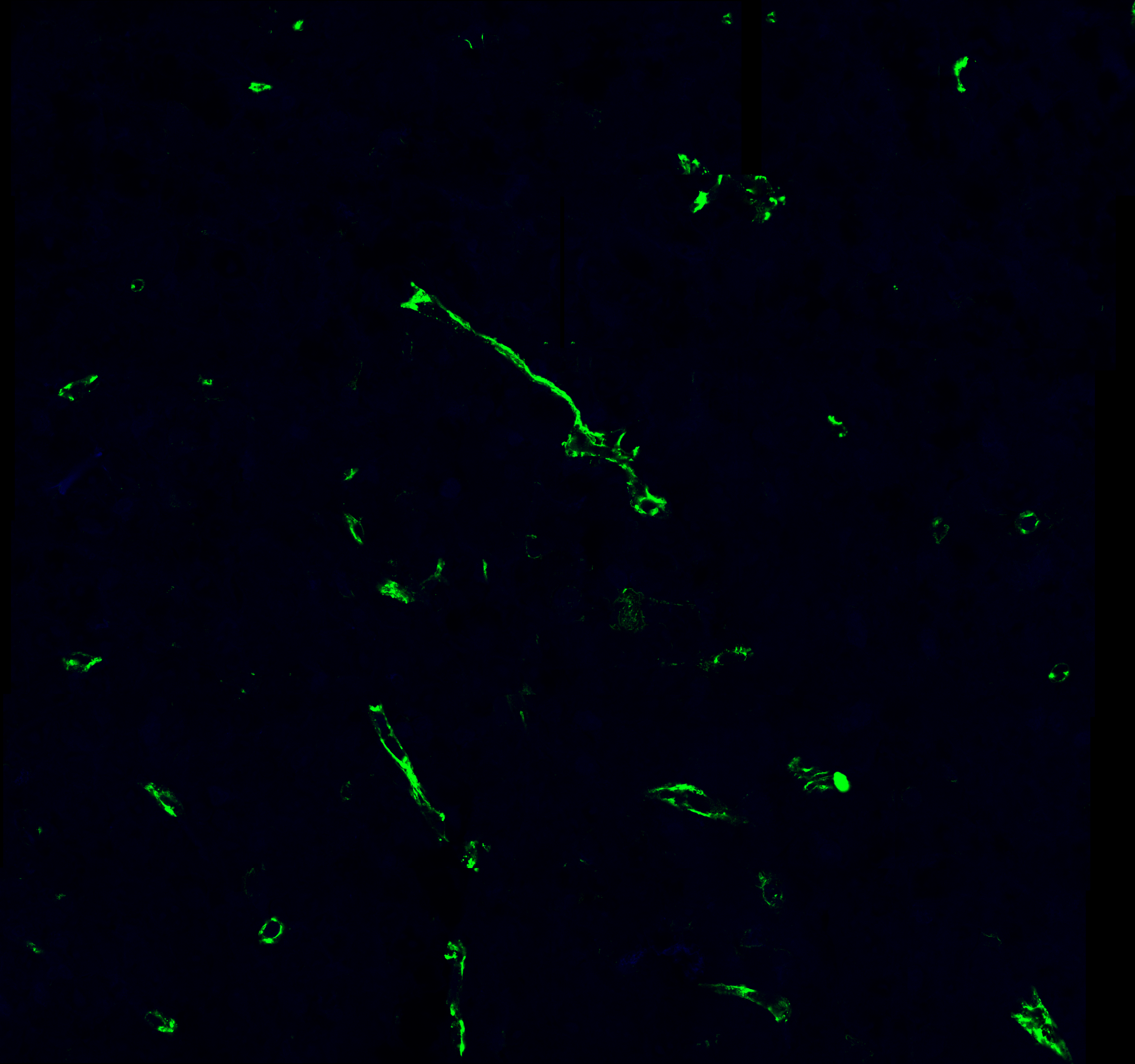

Supplement: Supplementary file 10 — Figures EV1 to EV5 Source data [file 44319_2026_799_MOESM10_ESM.zip › SourceData EV Figs/SourceData Fig EV3/Fig EV3A_.png images/Figure EV3 A 2.png]

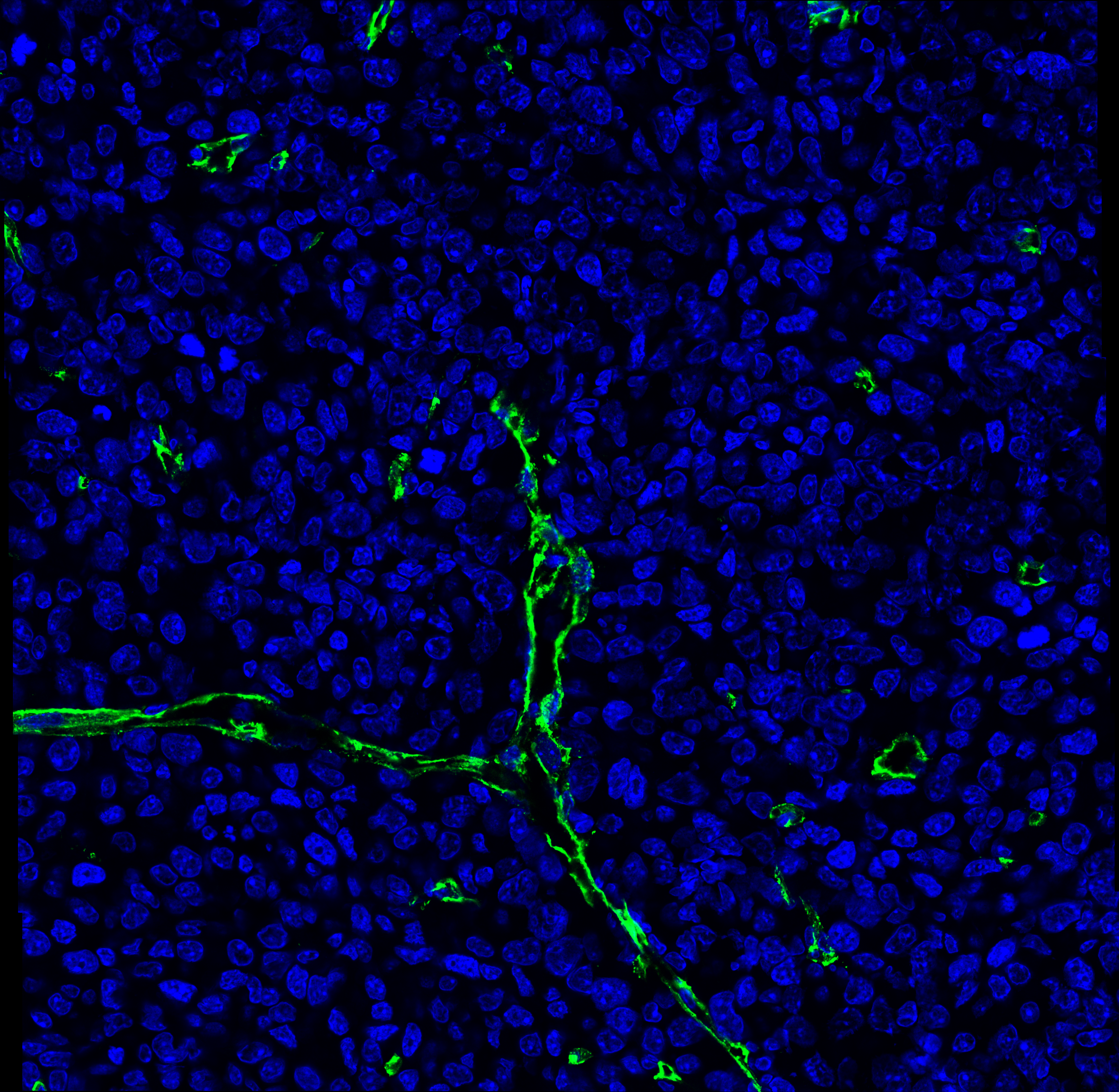

Supplement: Supplementary file 10 — Figures EV1 to EV5 Source data [file 44319_2026_799_MOESM10_ESM.zip › SourceData EV Figs/SourceData Fig EV3/Fig EV3A_.png images/Figure EV3 A 3.png]
