# Supplementary material for: Poorly perfused tumor regions harbor T cells with a glucose-dependent effector phenotype
Source: EMBO Rep. 2026 May 27;27(12):3454–87. doi: 10.1038/s44319-026-00799-0 (PMC13303879; doi:10.1038/s44319-026-00799-0)
Supplement: Supplementary file 11 — Expanded View Figures [file 44319_2026_799_MOESM11_ESM.pdf]

Expanded View Figures

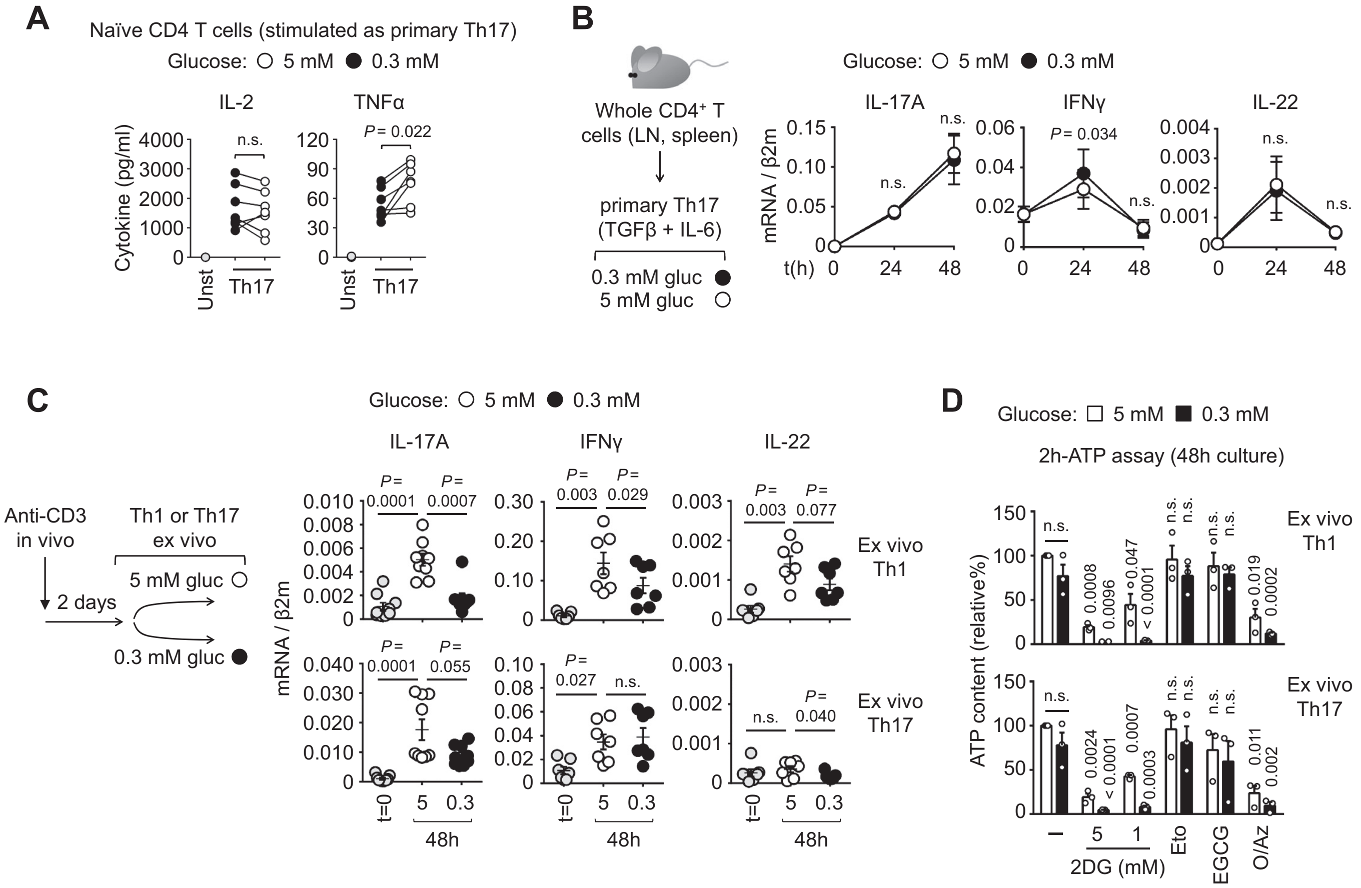

Figure EV1. Glucose restriction has a variable impact on Th1 and Th17 cytokine expression in CD4 cells activated under different conditions.

(**A**) Production of the indicated cytokines in naive CD4 T lymphocytes stimulated with anti-CD3 and anti-CD28 antibodies plus IL-2, TGFβ and IL-6 (primary Th17) in medium with normal (5 mM) or low (0.3 mM) glucose. Samples are the same as in Fig. 1A. Results show the mean ± SEM from seven biological replicates (different mice) in one experiment. Dots are connected by lines to better visualize differences between 5 and 0.3 mM glucose in each individual sample. Statistical significance was assessed with a paired *t* test. (**B**) Expression of the indicated cytokines in CD4 T lymphocytes freshly isolated from a mixture of lymph nodes and spleen, and stimulated with anti-CD3 and anti-CD28 antibodies plus TGFβ and IL-6 (primary Th17). Results show the mean ± SEM from two experiments, with two to three biological replicates (different mice) each. Statistical significance was assessed with a paired *t* test. (**C**) Cytokine mRNA expression in lymph node CD4 T cells preactivated in vivo with injected anti-CD3 antibody and isolated from the mice 2 days later to be restimulated in culture as Th1 (with IL-12) or Th17 (TGFβ and IL-6) for 48 h. Results show the mean ± SEM from seven to nine biological replicates. Statistical significance was assessed by a paired *t* test to compare the two conditions (0.3 and 5 mM glucose) for each sample. (**D**) Intracellular ATP content in Th1 or Th17 cells stimulated as in (**C**) for 48 h and then treated with the indicated metabolic inhibitors for the last 2 h. 2-deoxyglucose (2DG, 1 and 5 mM), epigallocatechin gallate (EGCG, 50 µM), etomoxir (Eto, 200 µM), and oligomycin plus sodium azide (0.1 µg/ml and 20 mM, respectively) (O/Az). Results show the mean ± SEM from three biological replicates. Statistical significance was assessed with a one-sample *t* test for comparison with the reference 5 mM glucose control sample without inhibitors. *P* values for the comparisons between each condition and the control are indicated with vertical values. Significant *P* values (<0.05) and *P* values between 0.05 and 0.1 are indicated. n.s. not significant. Source data are available online for this figure.

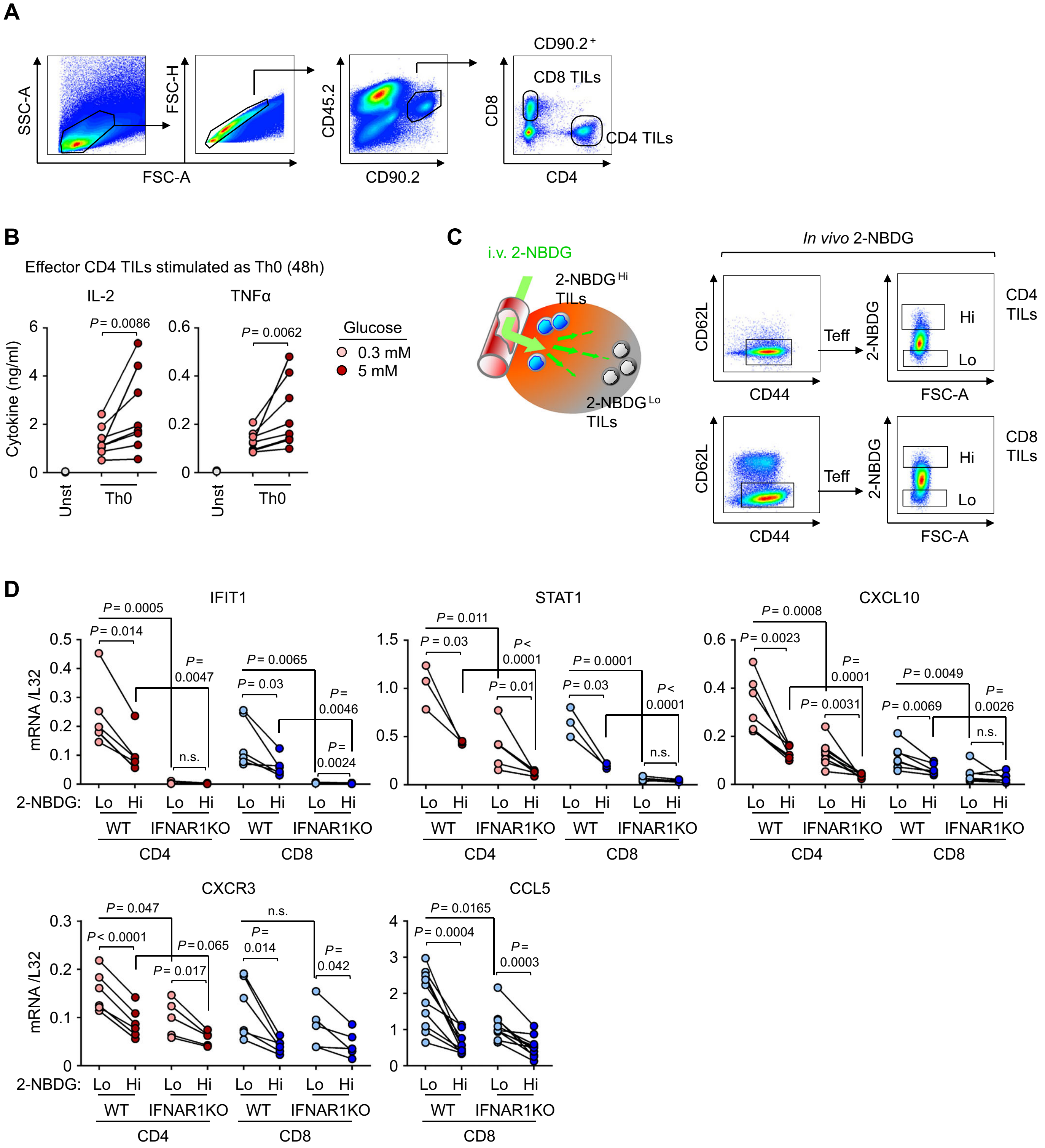
A
SSC-A
FSC-A
FSC-H
CD45.2
CD90.2
CD90.2⁺
CD8 TILs
CD4 TILs
CD8
CD4
B
Effector CD4 TILs stimulated as Th0 (48h)
IL-2
TNFα
Cytokine (ng/ml)
P = 0.0086
P = 0.0062
Glucose
0.3 mM
5 mM
Unst
Th0
C
i.v. 2-NBDG
2-NBDG Hi TILs
2-NBDG Lo TILs
In vivo 2-NBDG
CD62L
CD44
Teff
2-NBDG
FSC-A
Hi
Lo
CD4 TILs
CD8 TILs
D
IFIT1
STAT1
CXCL10
CXCR3
CCL5
mRNA /L32
P = 0.0005
P = 0.014
P = 0.0047
P = 0.0065
P = 0.03
P = 0.0046
P = 0.0024
n.s.
P = 0.011
P = 0.03
P < 0.0001
P = 0.01
P = 0.0001
P = 0.03
P < 0.0001
P = 0.0008
P = 0.0023
P = 0.0001
P = 0.0031
P = 0.0049
P = 0.0069
P = 0.0026
P = 0.047
P < 0.0001
P = 0.065
P = 0.017
P = 0.014
P = 0.042
P = 0.0165
P = 0.0004
P = 0.0003
2-NBDG:
Lo
Hi
WT
IFNAR1KO
CD4
CD8

 © The Author(s)

Figure EV2. Gating strategies for isolating CD4 and CD8 T lymphocytes infiltrating LLC tumors, and cytokine production analysis in TILs activated ex vivo.

(**A**) Flow cytometry gating strategies used to isolate CD4 and CD8 T lymphocytes infiltrating LLC tumors. (**B**) Production of IL-2 and TNFα by CD4 effector TILs freshly isolated by FACS sorting from LLC tumors and either left unstimulated or stimulated with anti-CD3 and anti-CD28 plus IL-2 (Th0 conditions) 48 h in medium with 5 mM or 0.3 mM glucose. Unstimulated controls were kept in medium with 5 mM glucose and IL-2 but without anti-CD3 and anti-CD28 antibodies. This experiment used TILs not traced with 2-NBDG nor Hoechst. Cytokine concentration was measured with Legendplex bead arrays. Results show 8 biological replicates (8 mice), and correspond to the samples shown in Fig. 3A. Statistical significance was assessed by a paired *t* test to compare the two conditions (0.3 and 5 mM glucose) for each sample. (**C**) Diagram illustrating the rationale of the experiment, and flow cytometry panels representative of effector memory (Teff) CD4 and CD8 TILs, and their uptake of intravenously injected 2-NBDG. (**D**) Expression of the indicated genes in effector CD4 and CD8 TILs sorted by their 2-NBDG uptake in vivo, in wild-type and IFNAR1-deficient mice (IFNAR1KO). Results are from three to ten biological replicates. Dots are connected by lines to better visualize differences between 2-NBDG ^{Lo} versus ^{Hi} cells within each individual tumor. Statistical significance was assessed with a paired *t* test to compare ^{Lo} and ^{Hi} cells within the same tumor, and with an unpaired *t* test to compare WT with IFNAR1KO. Significant *P* values (<0.05) and *P* values between 0.05 and 0.1 are indicated. n.s. not significant. Source data are available online for this figure.

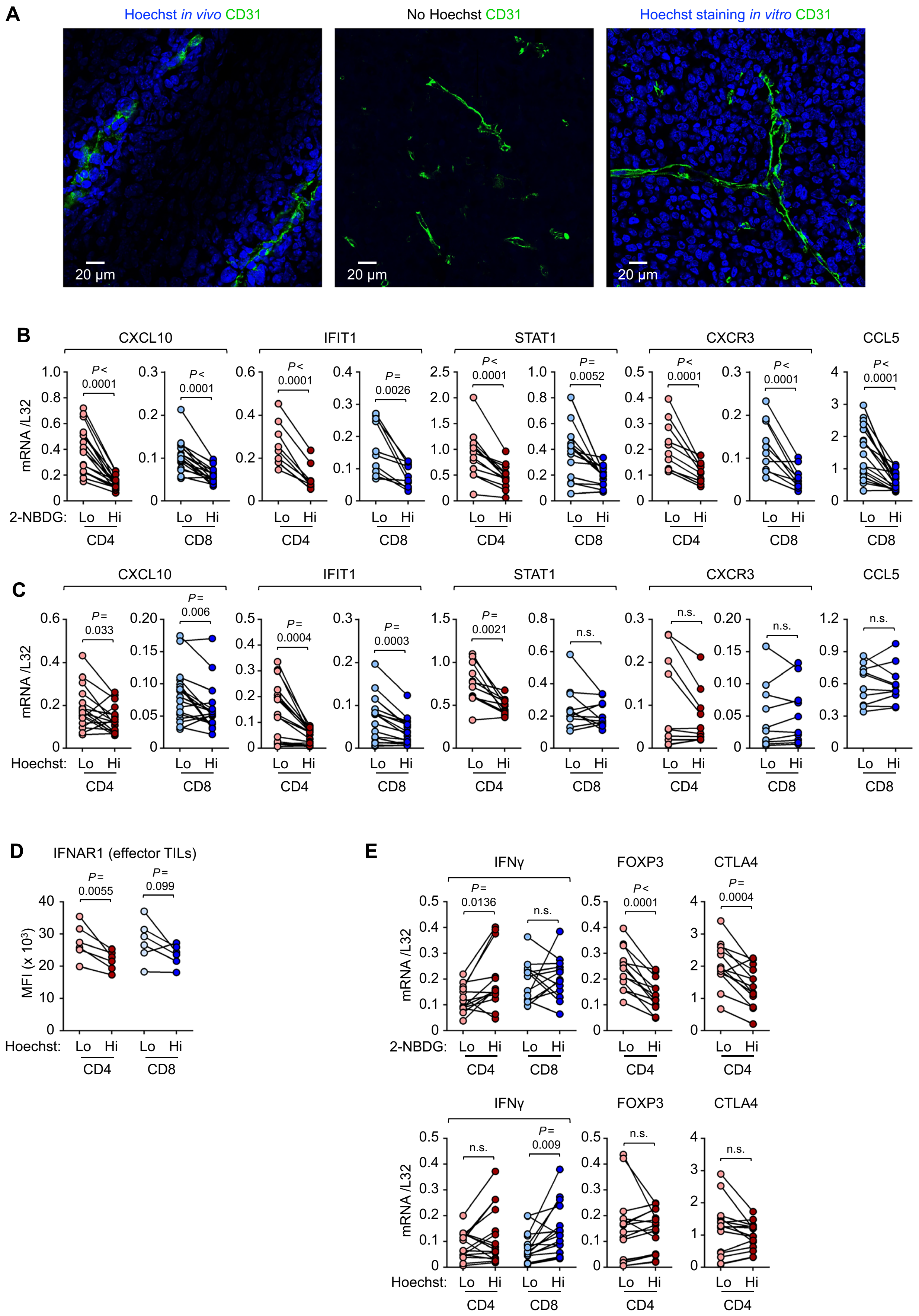
A
Hoechst in vivo CD31
No Hoechst CD31
Hoechst staining in vitro CD31
20 µm
20 µm
20 µm
B
CXCL10
IFIT1
STAT1
CXCR3
CCL5
mRNA /L32
P< 0.0001
P< 0.0001
P< 0.0001
P= 0.0026
P< 0.0001
P= 0.0052
P< 0.0001
P< 0.0001
P< 0.0001
2-NBDG: Lo Hi
CD4 CD8 CD4 CD8 CD4 CD8 CD4 CD8 CD8
C
CXCL10
IFIT1
STAT1
CXCR3
CCL5
mRNA /L32
P= 0.033
P= 0.006
P= 0.0004
P= 0.0003
P= 0.0021
n.s.
n.s.
n.s.
n.s.
Hoechst: Lo Hi
CD4 CD8 CD4 CD8 CD4 CD8 CD4 CD8 CD8
D
IFNAR1 (effector TILs)
MFI (x 10³)
P= 0.0055
P= 0.099
Hoechst: Lo Hi Lo Hi
CD4 CD8
E
IFNγ
FOXP3
CTLA4
mRNA /L32
P= 0.0136
n.s.
P< 0.0001
P= 0.0004
2-NBDG: Lo Hi Lo Hi
CD4 CD8 CD4 CD4
IFNγ
FOXP3
CTLA4
mRNA /L32
n.s.
P= 0.009
n.s.
n.s.
Hoechst: Lo Hi Lo Hi
CD4 CD8 CD4 CD4

 © The Author(s)

Figure EV3. Comparison of CD4 and CD8 effector TILs based on their uptake of 2-NBDG and Hoechst for the expression of the mRNAs of interferon-stimulated genes (ISGs), CXCR3, CCL5, and the expression of IFNγ, FOXP3 and CTLA4 mRNA.

(**A**) Confocal microscopy images showing: (left panel) the distribution of intravenously injected Hoechst 33342 (blue) in LLC tumor sections, together with endothelial cells (blood vessels, CD31 cells in green); (middle panel) blood vessels in a tumor section from a mouse not injected with Hoechst; and (right panel), blood vessels in a tumor section from a mouse not injected with Hoechst, but stained with Hoechst in vitro, showing a dense, homogenous distribution of cells (blue nuclei) in the tumor section. Scale bars are shown. Results are representative of two biological replicates (2 mice). (**B**, **C**) mRNA levels of the indicated genes in CD4 and CD8 effector TILs sorted as 2-NBDG ^{Lo} versus ^{Hi} cells (**B**) or Hoechst ^{Lo} versus ^{Hi} cells (C). Results in (**B**) comprise 9 to 18 biological replicates (3 to 5 experiments with 3 to 4 mice each). Results in (C) comprise 10 to 17 biological replicates (2 to 3 experiments with 4 to 7 mice each). (**B**) includes samples from the experiments shown in Fig. 3H plus additional ones, and (**C**) includes samples from experiments shown in Fig. EV5H (vehicle-treated mice) plus additional ones. Dots are connected by lines to better visualize differences between ^{Lo} vs ^{Hi} cells within each individual tumor. Statistical significance was assessed with a paired *t* test to compare ^{Lo} versus ^{Hi} cells within same tumor. (**D**) Surface expression of the IFN-I receptor chain IFNAR1 in Hoechst ^{Lo} versus ^{Hi} CD4 and CD8 effector TILs. Results are from 6 biological replicates (individual mice). (**E**) mRNA levels of IFNγ, FOXP3 and CTLA4 in CD4 and CD8 effector TILs sorted as 2-NBDG ^{Lo} versus ^{Hi} cells (upper panels) or Hoechst ^{Lo} versus ^{Hi} cells (lower panels). Results for 2-NBDG comprise 13 biological replicates (4 experiments with 3 to 4 mice each). Results for Hoechst comprise 15 to 16 biological replicates (3 experiments with 4 to 7 mice each). Data for 2-NBDG include samples from the experiments shown in Fig. 3H plus additional ones, and Hoechst data include samples from experiments shown in Fig. EV5H (vehicle-treated mice) plus additional ones. Dots are connected by lines to better visualize differences between ^{Lo} versus ^{Hi} cells within each individual tumor. Statistical significance was assessed with a paired *t* test to compare ^{Lo} and ^{Hi} cells within same tumor. Significant *P* values (<0.05) and *P* values between 0.05 and 0.1 are indicated. n.s. not significant. Source data are available online for this figure.

© The Author(s)

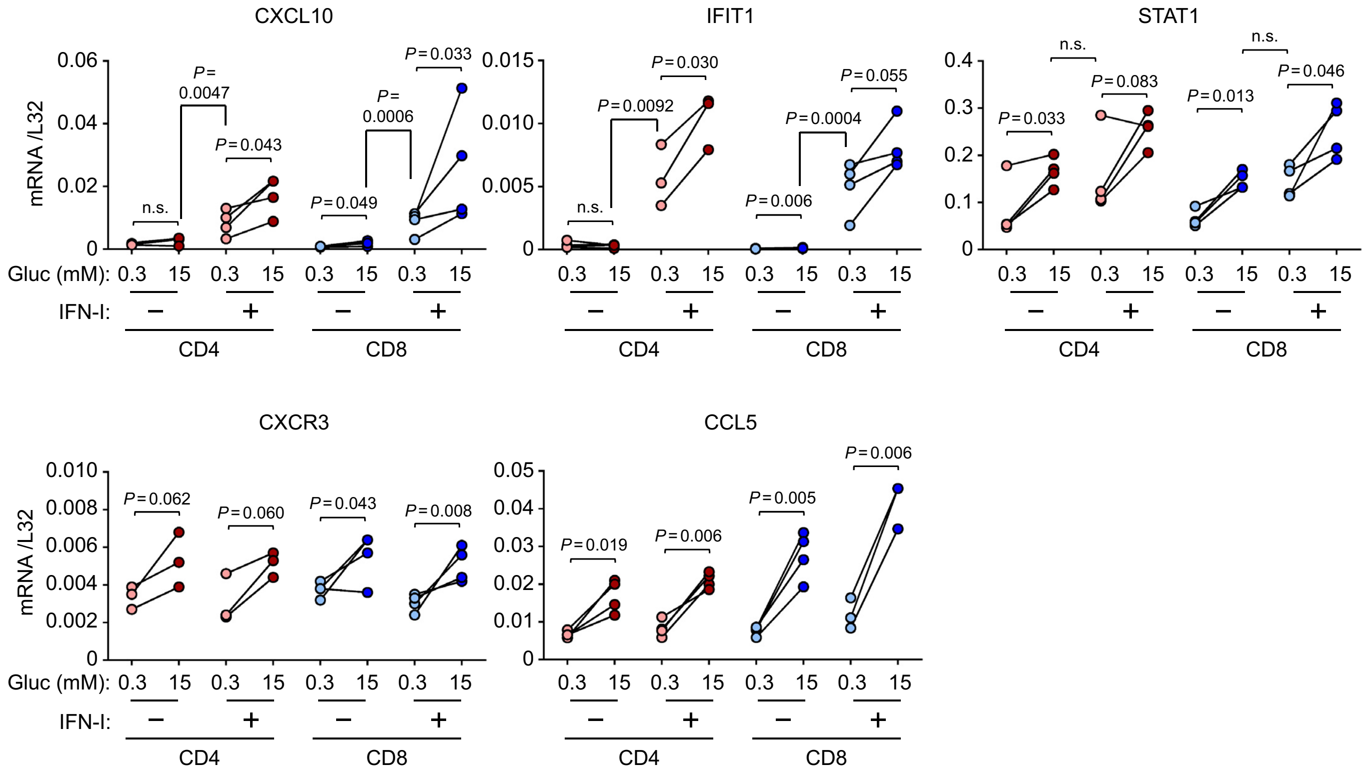

Figure EV4. Response to IFN-I and glucose sensitivity of different genes in in vitro-activated lymph node T lymphocytes.

mRNA expression of the indicated genes in T lymphocytes preactivated in vitro (5 days) and restimulated (24 h) with anti-CD3 and anti-CD28 without or with a cocktail of IFNα4 (600 U/ml) and IFNβ1 (2.5 ng/ml) (IFN-I) in culture medium with 0.3 or 15 mM glucose. T lymphocyte cultures were prepared from three to four biological replicates (independent mice). Statistical significance was assessed with a paired *t* test. Significant *P* values (<0.05) and *P* values between 0.05 and 0.1 are indicated. n.s. not significant. Source data are available online for this figure.

 © The Author(s)

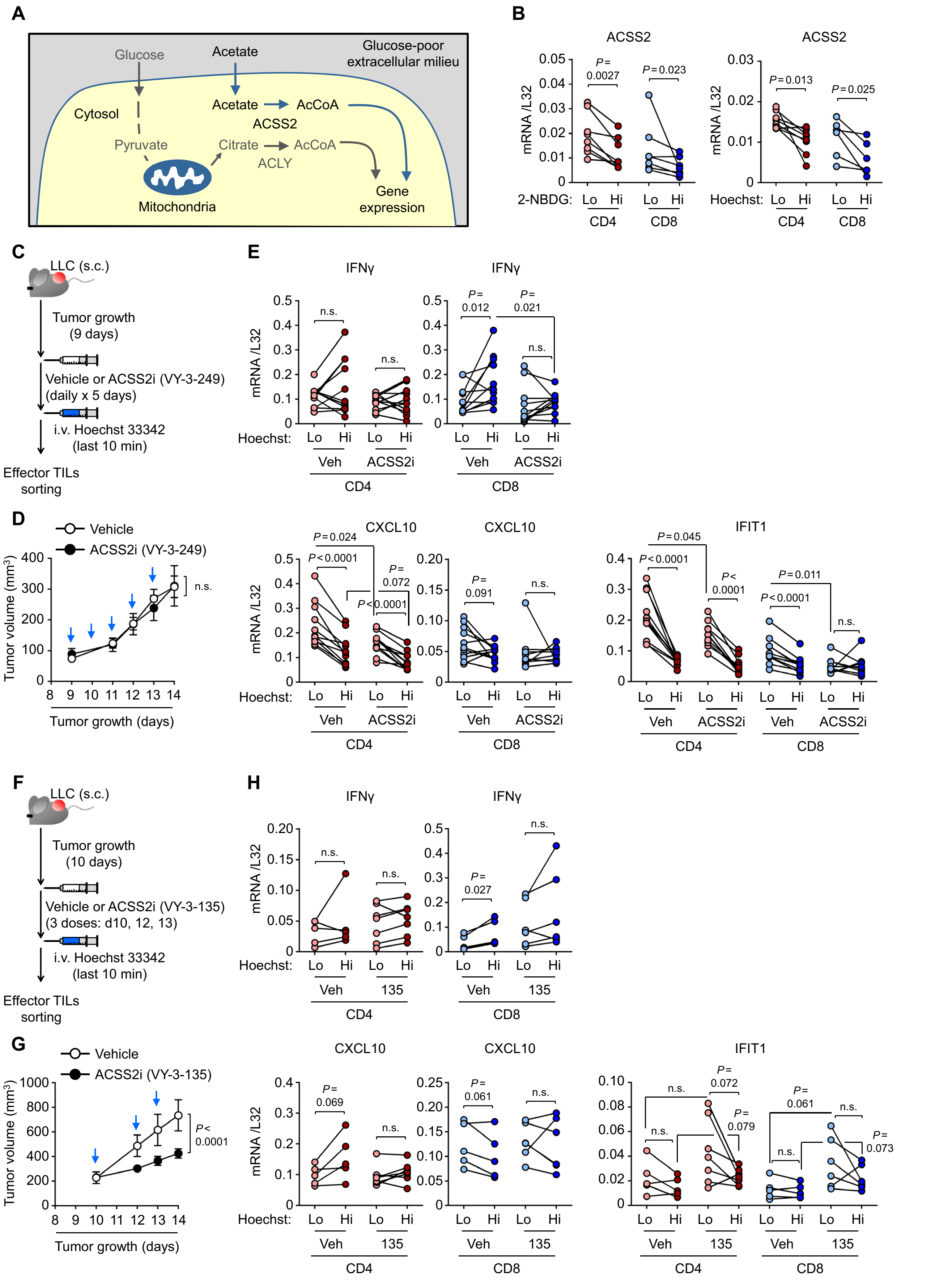
A
Glucose
Acetate
Glucose-poor extracellular milieu
Cytosol
Acetate → AcCoA
ACSS2
Pyruvate
Citrate → AcCoA
ACLY
Mitochondria
Gene expression
B
ACSS2
mRNA /L32
2-NBDG: Lo Hi Lo Hi
CD4 CD8
Hoechst: Lo Hi Lo Hi
C
LLC (s.c.)
Tumor growth (9 days)
Vehicle or ACSS2i (VY-3-249) (daily x 5 days)
i.v. Hoechst 33342 (last 10 min)
Effector TILs sorting
D
Vehicle
ACSS2i (VY-3-249)
Tumor volume (mm3)
Tumor growth (days)
n.s.
E
IFNγ
CXCL10
IFIT1
Veh ACSS2i
F
LLC (s.c.)
Tumor growth (10 days)
Vehicle or ACSS2i (VY-3-135) (3 doses: d10, 12, 13)
i.v. Hoechst 33342 (last 10 min)
Effector TILs sorting
G
Vehicle
ACSS2i (VY-3-135)
H
Veh 135

◀ **Figure EV5. ACSS2 inhibitors have different effects on gene expression in CD4 and CD8 TILs with high or reduced access to blood in vivo.**

(**A**) Diagram illustrating that T cells in a glucose-poor environment can obtain cytosolic acetyl-CoA (AcCoA) from imported extracellular acetate by using the enzyme acetyl-CoA-synthetase 2 (ACSS2). Glucose normally contributes to cytosolic AcCoA when the glycolysis product pyruvate is processed through the mitochondrial Krebs cycle, from which citrate exported to the cytosol is converted to AcCoA by ATP citrate lyase (ACLY) (diagram concept adapted from Qiu et al, 2019). (**B**) Expression of ACSS2 mRNA in CD4 and CD8 TILs sorted by their uptake of 2-NBDG or Hoechst 33342. Results in (**B**) are from six to eight biological replicates (from two independent experiments for 2-NBDG and Hoechst, with 3 to 6 mice per experiment). (**C**, **D**) Diagram of the in vivo experiment (**C**) and tumor growth in mice treated with vehicle or ACSS2i/VY-3-249 (25 mg/kg) (**D**), blue arrows indicate when the mice were treated. (**E**) mRNA expression of the indicated gene products in CD4 and CD8 effector TILs from mice left untreated or treated with vehicle or ACSS2i/VY-3-249 (ACSS2i), and then sorted by their uptake of Hoechst in vivo. Results in (**D**, **E**) are from 9 to 11 biological replicates (mice) per experimental group (2 independent experiments with 4 and 7 mice each). (**F**, **G**) Diagram of the in vivo experiment (**F**) and tumor growth in mice treated with vehicle or VY-3-135 (100 mg/kg) (**G**), blue arrows indicate when the mice were treated. (**H**) mRNA expression of the indicated gene products in CD4 and CD8 effector TILs from mice left untreated or treated with vehicle or VY-3-135 (abbreviated as 135), and then sorted by their uptake of Hoechst in vivo. Results in (**G**, **H**) are from 5 to 7 biological replicates (mice) per experimental group (1 experiment). Statistical significance was assessed with a paired *t* test (**B**, **E**, **H**) and a two-way ANOVA test (**D**, **G**). Significant *P* values (<0.05) and *P* values between 0.05 and 0.1 are indicated. n.s. not significant. Source data are available online for this figure.

 © The Author(s)
