# Supplementary material for: Poorly perfused tumor regions harbor T cells with a glucose-dependent effector phenotype
Source: EMBO Rep. 2026 May 27;27(12):3454–87. doi: 10.1038/s44319-026-00799-0 (PMC13303879; doi:10.1038/s44319-026-00799-0)
Supplement: Supplementary file 1 — Peer Review File [file 44319_2026_799_MOESM1_ESM.pdf]

EMBO reports

Poorly perfused tumor regions harbor T cells with a glucose-dependent effector phenotype

Marta Riera-Borrull, Víctor Cerdán Porqueras, Sonia Tejedor Vaquero, Claudia Tomaselli González, Mauricio Guzman, Donata Martinuzzi, Diego Ceacero-Heras, Andrea Cerutti, Jose Aramburu, and Cristina López-Rodríguez

Corresponding author(s): Cristina López-Rodríguez (cristina.lopez-rodriguez@upf.edu) , Jose Aramburu (jose.aramburu@upf.edu)

Review Timeline:

Transfer Date:	11th Dec 24
Editorial Decision:	17th Dec 24
Revision Received:	28th Sep 25
Editorial Decision:	12th Dec 25
Revision Received:	9th Apr 26
Accepted:	30th Apr 26

Editor: Achim Breiling

Transaction Report: This manuscript was transferred to EMBO Reports following peer review at Review Commons.

Review COMMONS

(Note: With the exception of the correction of typographical or spelling errors that could be a source of ambiguity, letters and reports are not edited. Depending on transfer agreements, referee reports obtained elsewhere may or may not be included in this compilation. Referee reports are anonymous unless the Referee chooses to sign their reports.)

Revision Plan

Manuscript number: RC-2025-02596
Corresponding author(s): Cristina López-Rodríguez

[The "revision plan" should delineate the revisions that authors intend to carry out in response to the points raised by the referees. It also provides the authors with the opportunity to explain their view of the paper and of the referee reports.

The document is important for the editors of affiliate journals when they make a first decision on the transferred manuscript. It will also be useful to readers of the reprint and help them to obtain a balanced view of the paper.

If you wish to submit a full revision, please use our "Full Revision" template. ***It is important to use the appropriate template to clearly inform the editors of your intentions.****]*

1. General Statements [optional]

This section is optional. Insert here any general statements you wish to make about the goal of the study or about the reviews.

Summary of the goal and rationale of our study, as well as its main findings.

A main goal of our work was to analyze the characteristics of effector T cells in tumor regions with substantially restricted access to blood. Previous works have studied metabolic and nutrient utilization characteristics of T cells in tumors, but had not distinguished between cells with higher and lower access to blood-transported nutrients. Addressing this question is relevant to better define the heterogeneity of T lymphocytes in the tumor and to understand their potential in response to immunotherapeutic approaches.

Effector T cells are avid nutrient consumers, and in the tumor microenvironment they compete for nutrients with cancer cells and other immune cells. This competition is more intense in poorly perfused regions, where T cells may not obtain enough nutrients to sustain antitumor function; and it is generally thought that the anti-tumor capability of T cells in inner tumor regions will be largely limited due to nutrient insufficiency. However, previous works have shown that T cells in nutrient-poor tumor microenvironments can respond to stimulation with anti-checkpoint antibodies, which led us to think that T cells might possibly be more competent than commonly thought in metabolically challenging microenvironments. Here we have asked which features identify effector CD4 and CD8 T lymphocytes in different regions within the tumor, where they face different metabolic conditions, and compared them with tumor-inexperienced CD4 T cells activated *in vitro* in well-defined glucose-restricted conditions.

In the first part of our manuscript, we used *in vitro* assays to ask how CD4 T cells preactivated in different ways would respond to restimulation via T cell receptor in glucose-poor conditions. We chose glucose restriction because activated effector T cells are heavy glucose consumers and much dependent on glucose to maintain their functions. We found that CD4 T cells activated as Th17 or Th1 under severe glucose restriction were capable of maintaining an activated state for days and even induce diverse glucose-dependent cytokines. Notably, T cells activated in low glucose, down to levels reported in tumors and less than 50 times lower than in blood, still used glucose as their main fuel for ATP production, which altogether suggested that they can display substantial functionality with little glucose.

These results were intriguing, but glucose is just one nutrient, and so we asked which characteristics could be expected in T cells *in vivo* in a metabolically challenging milieu with reduced access to blood-transported nutrients. To address this, we reasoned that tumor-infiltrating T cells (TILs) more likely to be subjected to a general nutrient deficiency would be found in tumor regions poorly perfused by blood, and used the uptake of intravenously injected fluorescent probes by T cells as an indicator of their accessibility to blood-transported nutrients. This approach allowed us to isolate and characterize different populations of effector CD4 and CD8 TILs *in vivo*.

We have found that effector T lymphocytes infiltrating tumors express different gene signatures in regions with different accessibility to blood, and can maintain specific glucose-dependent responses even in poorly perfused tumor regions. The novelty of our work is the identification of specific features that distinguish effector TIL subsets by their accessibility to blood, and the finding that effector CD4 and CD8 T cells in poorly accessible tumor regions do not just show some features expected in nutrient-restricted cells, but exhibit characteristics of activated T cells that also suggest potentially enhanced antitumor capacity. Our results open new paths for exploring the functional heterogeneity and compartmentalization of TILs in tumor regions with higher or lower accessibility to blood.

2. Description of the planned revisions

Insert here a point-by-point reply that explains what revisions, additional experimentations and analyses are planned to address the points raised by the referees.

We appreciate the work done by the reviewers and their thoughtful comments and recommendations for improvement. As detailed in the description of the planned revision, we are confident that we can address experimentally most of the reviewers' questions, which we expect it will enhance the significance and interest of our manuscript.

Point-by-point reply to reviewers with the plan to address their points:

Reviewer #1 (Evidence, reproducibility and clarity (Required)):

The research article "Effector T cells in poorly perfused tumor regions exhibit a distinct signature of augmented IFN response and reduced PD-1 expression" by Riera-Borrull et al. investigates the glucose-dependent functions of Th17 and Th1 cells activated under very low glucose concentrations similar to those encountered in tumors. This research has the potential to significantly impact our understanding of T cell function in tumor microenvironments. Their main claim is that T lymphocytes can achieve substantial activation and maintain energy sufficiency under deficient levels of glucose. In addition, they also claim that effector TILS with very low accessibility to glucose (blood) express multiple glucose-dependent genes and markers associated with enhanced antitumor capacity.

The study is clear and easy to follow, significant in the field, and has well-described and, in principle, adequate experimental procedures. However, the authors need to perform additional experiments to fully support their claims.

We thank the Referee for their appreciation of our work and their positive overall assessment. We are also grateful for the insightful comments and suggestions below.

Major comments:

-Figure 1. The authors start the experiments by placing polyclonally activated CD4 lymphocytes under basal (5 mM) or low glucose levels (0.3 mM) and studying the expression of hallmark Th17 and Th1 cytokines. They compared the expression of these cytokines using two conditions: 1) CD4 T cells isolated from LN and spleen activated with anti-CD3 and anti-CD28 and differentiated towards Th17 (TGF-β and IL-6) with low or high glucose concentrations. 2) Activation of lymphocytes from LN in the presence of IL-2 for 5 days, followed by the re-activation with anti-CD3 and CD28 and polarization toward Th17. The experimental design is incorrect. Cells from condition 1) are correctly termed 1ry Th17, but the cells so-called 2ry Th17 are not Th17. In order to really compare the two types of Th17, a condition where cells are polarized twice toward Th17. Can Th17 cells, freshly activated and differentiated in a normal glucose medium, produce the same amount of cytokines as cells in a low glucose medium? Are the cells from condition 1 naïve or only isolated CD4 T cells?

We thank the Referee for the correction about our having named the cells 1ry and 2ry Th17 cells. This will be corrected in the revised version of the manuscript and preactivated T cells restimulated with TGF-β and IL-6 will not be referred to as 2ry Th17. Also, cells from condition 1 were freshly isolated from LNs, but not naïve. This will be made clear in the revised manuscript.

Regarding the question of whether freshly activated Th17 cells would produce the same amount of cytokines in normal or low glucose medium, our results show they do at the mRNA level. Measurement of cytokine protein levels produced by these cultures in 5 or 0.3 mM glucose will

be included in our revision plan.

-Figure 2. The authors use a very nice approach, injecting anti-CD3 antibodies to activate T cells in vivo. Nevertheless, they take total CD4 T cells without looking for the activated ones, for example, by sorting using activation markers such as CD25, CD69, or even CD62L and CD44 to take the Teff cells. In addition, the Isotype IgG control is missing, and the control t=0 included in the figures is insufficient.

We agree with the referee that an analysis of sorted effector T cells activated *in vivo* would be needed. In this regard, we think that the results in Figure 2 might be a bit redundant with those in Figure 1 and Figure S1C for the analysis of ATP sources, as the three figures show that T cells stimulated in culture in low glucose can still use glucose as a main fuel for ATP production and also increase their dependence on mitochondrial respiration. Likewise, both Figures 1 and 2 show that expression of several cytokines is affected to a greater or lesser extent by low glucose depending on how T cells were stimulated. As the next figures and the main focus of the manuscript are on tumor-infiltrating T cells (TILs), we propose to do our revision with TILs sorted as effector, which already show characteristics of activated T cells.

-Figure 3. The title is misleading as the authors study both CD8 and CD4. The gating strategy described in Sup2 is insufficient. What is the expression of other immune checkpoint genes such as TIM-3, PD-1, or LAG-3? Can authors complement Figure 3D with cytometry to know the protein levels? The same with Figure 6, can the authors show protein levels (IFNγ, CTLA4, Foxp3)

We see the point of the Reviewer with the figure title, and we will change it to reflect that the figure shows results in CD4 and CD8 cells. Likewise, we will include the complete stepwise gating strategy used for the sorting of 2-NBDG ^{Hi} and ^{Lo} cells. Regarding the expression of TIM-3, PD-1 and LAG-3, PD-1 was analyzed in Figure 7, where we show that 2-NBDG ^{Lo} and Hoechst ^{Lo} CD4 and CD8 T cells have lower surface expression of PD-1 than their 2-NBDG ^{Hi} and Hoechst ^{Hi} counterparts. For TIM-3 and LAG-3, our RNA-seq data show moderately higher expression of TIM-3 (*Havcr2*) and LAG-3 in 2-NBDG ^{Lo} effector CD8 TILs than in 2-NBDG ^{Hi} ones, but fewer reads for both in 2-NBDG ^{Lo} than in 2-NBDG ^{Hi} effector CD4 TILs. Our revision plan will analyze protein levels for these markers in Hi/Lo CD4 and CD8 TILs. Likewise, we plan to analyze the expression of IFNγ, CTLA4, and Foxp3 proteins in the experiments of Figure 6.

-Figure 7. To determine the functionality of the CD4 and CD8 TILS, the authors need to transfer those cells to congenic (CD45.1) tumor-bearing mice and see if they can conserve the described phenotype.

We agree with the Reviewer and appreciate this suggestion. However, a substantial limitation that we will find in this approach is the very small number of effector TILs in the 2-NBDG (or Hoechst) Hi and Lo subsets we can get after sorting. This number is in the hundreds of cells of each subset per tumor, far below to what we would need for adoptive transfer assays.

Nonetheless, we think that even with few cells we could try some experiments to test their functionality and infer their antitumor potential better than by only analyzing gene expression. For instance, testing CD107a degranulation as an indicator of cytotoxic capacity, which would also complement our protein expression analyses in the previous point. We plan to carry out these assays in our revision plan. In addition, we plan to expand TILs in culture in normal or nutrient-depleted medium, which we hope would yield sufficient cells for testing their anti-tumor capacity in adoptive transfer assays. One potential problem with this experiment though is that TILs expanded *ex vivo* might adapt to the new culture conditions and lose part of the original characteristics they had in the tumor. Nonetheless, we think it is worth trying it and see how they compare in the expression of relevant ISGs and activation and polarization markers with freshly isolated 2-NBDG (or Hoechst) Hi and Lo TIL subsets, as this approach could inform about their distinct functional profiles.

- Are the claims and the conclusions supported by the data or do they require additional experiments or analyses to support them?

The conclusions are supported, despite the needed experiments and some flaws in the controls. However, further investigation would be beneficial.

- Please request additional experiments only if they are essential for the conclusions. Alternatively, ask the authors to qualify their claims as preliminary or speculative, or to remove them altogether.

See above

- Are the suggested experiments realistic in terms of time and resources? It would help if you could add an estimated time investment for substantial experiments.

The suggested experiments are easy to perform, and maybe the authors already have some of them. I would say that three months would be enough.

- Are the data and the methods presented in such a way that they can be reproduced?

Yes, they are well described and can be reproduced.

- Are the experiments adequately replicated and statistical analysis adequate?

Yes

Reviewer #1 (Significance (Required)):

Provide contextual information to readers (editors and researchers) about the novelty of the study, its value for the field and the communities that might be interested.

The study is novel and studies an important subject: the conditions encountered by TILS, poor glucose access, and, therefore, metabolic pressure. The subject is important, and the approach is adequate. There are some flaws in the experimental design, mostly in the in vitro/ex vivo experiments, but they can be resolved by the authors.

We thank the Reviewer for their positive assessment of our manuscript and for their thoughtful ideas to better strengthen our original findings.

The following aspects are important:

- Audience: basic and translational research in cancer but also applicable to other diseases such as autoimmune disease where T cell metabolism is pivotal.

- Please define your field of expertise with a few keywords to help the authors contextualize your point of view. Indicate if there are any parts of the paper that you do not have sufficient expertise to evaluate.

Immunology of T cells, cancer, immunotherapy

Reviewer #2 (Evidence, reproducibility and clarity (Required)):

Summary
In this manuscript, Riera-Borrull et al. first analyzed how glucose restriction might affect T lymphocyte function in vitro and found that T cells were capable of expressing certain cytokines such as IFNy and IL17A after stimulation, even with limited glucose availability. They then conducted in vivo experiments to assess expression profiles in tumor infiltrating T cells (TILs) in vivo with differential uptake of the glucose analog (i.e. 2-NBDG) and their accessibility to blood. Their findings revealed that 2-NBDGlo cells (i.e., presumably cells with less glucose uptake) exhibited a pronounced type I interferon signature compared to the 2-NBDGhi cells. Additionally, the uptake of 2-NBDG correlated with accessibility of TILs to blood vessels. The authors concluded that TILs with reduced access to blood upregulate ACSS2 to produce cellular acetyl-CoA, thereby sustaining gene expression, including ISGs. Lastly, the author observed that limited access to blood increased CXCR3 expression but decreased PD-1 expression in effector CD4 and CD8 TILs.

Major comments:

Overall, the story is highly descriptive. For example, cells consuming more glucose exhibit the same cellular ATP levels as the cells with limited glucose (Figures S1B and C). Why is this the

case? Do these cells have the same mitochondrial respiration, TCA cycle activity and etc? Additionally, although glucose restriction exerts similar effects on ATP levels in Th1 and Th17 cells ex vivo, the cells display different cytokine expression changes in response to varying glucose concentrations, suggesting that glucose-derived ATP may not be the sole factor impacting cytokine expression in those cells. Therefore, additional experiments such as seahorse and 13C-labeled glucose tracing are required to further characterize the role of glucose in mediating cytokine expression in those cells. Moreover, the cytokine expression in stimulated T cells can be dynamic. Thus, a few more time points would be helpful to more precisely assess how glucose availability affects cytokine expression.

We appreciate the Reviewer's summary of our manuscript and the interesting points raised. We agree that our story is highly descriptive, but we think that the novelty of the findings we describe, as well as that they were not obviously predictable nor expected from the current literature makes our results highly original. Regarding their comments, some we can discuss here, whereas others we plan to address experimentally.

Our experiments show that T cells activated in culture in low glucose still use glucose as a main nutrient for ATP production, but are also more dependent on mitochondrial respiration (sensitivity to oligomycin and sodium azide) than cells in higher glucose (Figures 1F, 2B and S1C). In view of these results, we think that Seahorse experiments would provide similar conclusions, that cells in low glucose would be more dependent on respiration to sustain ATP. In this regard, early experiments by the Thompson lab showed that T cells activated with anti-CD3 plus anti-CD28 antibodies in 0.4 mM versus 11 mM glucose increased oxygen consumption, which the authors discussed as being necessary for maximal ATP generation under limited glucose supply (Frauwith K et al., (2002) *Immunity* 16:769). Also, Argüello and colleagues used SCENITH analysis, which quantifies protein translation in the presence of metabolic inhibitors as the equivalent to ATP availability, and found that it recapitulates Seahorse metabolic profiling, also showing that CD8 and CD4 T cell naïve versus effector memory subsets were all substantially and comparably dependent on glucose as a main energy source, but varied widely in their dependence on mitochondrial respiration (Argüello R et al., (2020) *Cell Metab*. 32:1063). Here we have used the same conceptual idea and tested the dependence of ATP on specific pathways by using metabolic inhibitors, as it is done with the SCENITH or Seahorse methods, but measuring intracellular ATP directly.

As to the question of how cells in low glucose can maintain similar ATP levels as those in higher glucose, our interpretation is that this can be so because they spend less ATP in low glucose conditions. For example, they do not proliferate (Figure 1D), and also have reduced mTORC1 activity (Figure S1D). Both effects would be expected to reduce overall translation and transcription rates, in turn reducing ATP expenditure (Argüello R et al., (2020) *Cell Metab.* 32:1063; He S., et al (2011) *PLoS ONE* 6:e20107).

We agree with the Reviewer that glucose-derived ATP may not be the sole factor impacting cytokine expression in our experiments, and that other glucose-derived metabolites could

influence the expression of different genes to varying degrees. However, regarding 13C-glucose tracing, if we wanted to trace glucose to cytokine expression, we would probably need to identify quantitative changes in glucose-derived metabolites affecting transcription regulatory processes in cells with low glucose availability. One parameter to analyze would be the incorporation of 13C-glucose-derived acetyl-CoA or glycosylation intermediates in histones and transcription factors, since T cell gene expression can be regulated by histone acetylation (Peng M et al., (2016) *Science* 354:481), and glycosylation and acetylation of transcription factors, for instance NF-kappaB and FOXP3 (Chen LF et al (2002) *EMBO J* 21:6539; Ramakrishnan P et al., (2014) *Science Signaling* 6:ra75; Loosdregt J et al., (2010) *Blood* 115:965; Gao C et al., (2023) *BBA-Gene Regulatory Mechanisms* 1866:194992). However, the problem here is to know how the label is distributed in promoters of glucose-sensitive genes, as it would be necessary to separate a precise chromatin region of interest from the rest of the cell chromatin and analyze glucose-derived posttranslational modifications in histones and transcription factors there. Techniques of reverse chromatin immunoprecipitation coupled with high-resolution mass spectrometry has been described to identify protein complexes at specific chromatin regions (MacKenzie T et al., (2023) *Cells* 12:1860). However, this is a very difficult methodology when non-repetitive regions such as promoters or enhancers are studied, and we have neither the expertise nor the instrumentation for approaching it and identifying the 13C labels in the relevant proteins. We respectfully think that these experiments, while interesting, would be out of our reach and perhaps would not contribute substantially to other points of the manuscript raised by the Reviewer, which we think are more relevant to address.

We appreciate the Reviewer's suggestion that more time points could give us more precise information about cytokine expression, which will complement our current results. Nonetheless, our ELISA experiments in Figure 1C, measuring the cytokines accumulated in T cell culture supernatants 24 and 48 hours after activation, already suggest that at least IFNγ and IL-17 seem to plateau by 48 hours under either low or normal glucose.

The authors identified enhanced type I interferon signaling in 2-NBDGlo cells but did not investigate this further. Questions remain, such as the source of IFN (tumors, T cells or other cells) and why 2-NBDGlo cells more responsive to IFN. The lack of explanations or mechanistic studies renders these results less compelling.

We show that enhanced expression of type I interferon-response signature genes is a distinctive feature of 2-NBDG ^{Lo} and Hoechst ^{Lo} cells, and assessed the degree of dependence of these genes on the IFN alpha receptor (IFNAR) in experiments with IFNAR1 knockout mice. In other contexts, it has been found that bystander T cells that are not actually responding to antigens can be activated by local IFN-I, for instance in bystander-activated resident memory T cells in the lung (Low JS et al., (2020) *J Exp Med* 217:e20192291), or in CD8 infiltrating MC38 tumors (Lee KJ., (2024) *Cell Reports Med* 5:101567); and consequently display a characteristic ISG signature.

We also coincide with the Reviewer in the importance of knowing the sources of IFN-I in the tumor. In order to identify possible sources of IFN-I in the tumor, in our revision plan we will isolate different immune cell types, as well as tumor and stromal cells to analyze their expression of type I IFNs. As to why 2-NBDG ^{Lo} T cells respond better, the experiments above could explain it if we find that T cells or other cells in low accessibility regions express higher levels of IFNs. In addition, we will test whether 2-NBDG ^{Lo} TILs express more IFNAR than 2-NBDG ^{Hi} ones, and will analyze their intracellular levels of phospho-STAT1 as a potential indicator of stronger IFNAR signaling. We have already seen that 2-NBDG ^{Lo} TILs have higher expression of STAT1 mRNA (an IFN-I-induced gene) (Figures 4C, 4D and 6C), which makes it possible that they could accumulate more STAT1 protein to transduce IFNAR signals.

The authors stated "2-NBDG uptake is thought to correlate with the metabolic activity of T cells (37), and our results below indicate that TILs with poor 2-NBDG uptake resemble T cells under glucose limitation" (Page 10, lines 200-201). However, the author should validate that 2-NBDGlo cells indeed have lower glucose concentration compared to the 2-NBDGhi cells. Otherwise, conclusions based on this assumption are not convincing. For instance, ISGs were reduced in T cells in low glucose medium but augmented in 2-NBDGlo cells, contradicting the statement that "our results below indicate that TILs with poor 2-NBDG uptake resemble T cells under glucose limitation"!

We thank the Reviewer for pointing this out, as it needs to be rewritten in a clearer way to avoid confusing statements. In view of our results, we would conclude that TILs with poor 2-NBDG uptake exhibit some features found in T cells under glucose limitation (as we wrote at the end of that section of results), but also exhibit higher expression of diverse glucose-responsive ISGs. One possible explanation, which we plan to address experimentally, could be that there was more IFN-I in the local microenvironment of 2-NBDG ^{Lo} cells and even if they had low glucose, they would still induce higher expression of ISGs than cells in a more blood-accessible region. This is consistent with our results in Supplementary Figure S2 showing that adding IFN-I to T cells activated in low-glucose induces a higher expression of ISGs (CXCL10, IFIT1, STAT1) than stimulating cells in normal glucose but without extra IFN-I. We should also include in the paper the discussion that T cells displaying signs of being under limited glucose availability does not mean that they are starved of glucose and therefore they could maintain some glucose-dependent functions while downregulating others. This notion connects with recent results of the Rathmell lab (reference 24 in our manuscript) showing that on average TILs in subcutaneous MC38 tumors take as much glucose per cell as the bulk of the whole tumor, and even more than T cells in the spleen do, which suggests that TILs could have enough glucose to maintain significant functionality. However, this work did not separate TILs in high versus low glucose uptake, and it is likely that our 2-NBDG ^{Lo} / Hoechst ^{Lo} cells might represent the subsets with the lowest, but not entirely lacking, nutrient availability in a generally glucose-sufficient microenvironment. Besides rewriting these sentences, we hope that the experiments we will do to address the previous and next points would provide additional answers.

Some conclusions lack solid supporting evidence. Although the authors cited previous studies, certain experiments are necessary to substantiate their conclusions. For example, the authors concluded that 2-NBDGlo cells upregulate ACSS2 to produce acetyl-CoA for gene expression without actually measuring acetyl-CoA in those cells. We do not know whether: 1) 2-NBDGlo cells have low glucose; 2) 2-NBDGlo cells have low acetyl-CoA (Acetyl-CoA can be generated through other routes, such as fatty acid oxidation); 3) Increased ACSS2 necessarily leads to more acetyl-CoA production in those cells.

ACSSi experiments are also unconvincing, as the authors do not provide any data to demonstrate that the inhibitors function properly and that the downstream effects are not due to off-target effects. Moreover, it is possible that TILs with different accessibility to blood take up different amounts of the ACSSi, which could affect the comparison of cytokine expression in Hoechst hi/lo cells.

These are valid points and indeed we thought about them while working on this manuscript. One of our main concerns here when trying to measure intracellular acetyl-CoA or glucose in Hoechst or 2-NBDG ^{Hi} versus ^{Lo} (Hi vs Lo) effector CD4 or CD8 cells is that the sorting of these subsets yields very few cells (in the range of a few hundreds) from each tumor, and so we might be unable to detect these molecules. In case we could not detect acetyl-CoA or glucose in so few cells after sorting, we can ask about acetyl-CoA and glucose levels in TILs expanded *ex vivo*, which we hope would allow us to obtain more cells, and then cultured in nutrient-rich or nutrient-poor medium, without or with acetate supplementation. If these experiments worked, we could use them as well to test the effect of ACSS2 inhibitors on the intracellular levels of acetyl-CoA in TILs grown in glucose-sufficient or restricted conditions.

As to the possibility that TILs with different accessibility to blood take up different amounts of ACSS2i, we can discuss that our results show that whereas IFNγ is more inhibited by ACSS2i in more accessible cells, the opposite is seen with IFIT1, whose expression is more repressed by ACSS2i in less accessible cells (Figure 6C). In the same experiments, expression of CXCL10, CTLA4 and FOXP3 mRNA in CD4 effector TILs was comparably inhibited by ACSS2i in higher and lower accessibility cells. Our interpretation of these results is that ACSS2i, after an administration regimen of 5 consecutive daily injections, could inhibit the expression of sensitive genes in both high and low accessibility regions. Nonetheless, the sensitivity of specific genes to ACSS2 inhibitors could be tested in TILs expanded in culture to ensure homogenous access to the inhibitors. With regard to the off-target effects of ACSS2i, we plan to use two chemically independent drugs, ACSS2i (VY-3-249), and the more recently developed and more potent VY-3-135, which we expect will reduce the likelihood that convergent results are due to off-target ACSS2i effects.

Reviewer #2 (Significance (Required)):

Significance
The major issue of this study is the lack of mechanistic insights, which significantly diminishes

both impact and novelty of the findings. Much of the data is descriptive. The findings are not particularly exciting, also partially due to the absence of mechanisms. With more mechanistic insights and solid characterizations, the study could be of interest in the fields of immunometabolism and tumor immunotherapy.

We agree that carrying out the diverse experiments proposed by the reviewers will yield new mechanistic insights to improve our work. However, we would like to point out the novelty of our manuscript in uncovering the remarkable efficiency of activated T cells to maintain glucose-dependent functions under very low glucose levels, and identifying markers of tumor-infiltrating, effector CD4 and CD8 T cells that differ between cells with higher and lower accessibility to blood, such as the expression of IFN-I response signatures, the chemokine receptor CXCR3, and PD-1. Previous works analyzing the function and metabolic characteristics of tumor-infiltrating T cells had not identified specific features of effector TIL subsets distinguishable by their accessibility to blood. Our findings here offer an alternative scenario to the commonly held view that T cells in poorly perfused tumor regions are largely inactive due to nutrient insufficiency.

My expertise includes metabolism and cell signaling. I find that the metabolic studies in this manuscript are disappointing and require further investigations.

Reviewer #3 (Evidence, reproducibility and clarity (Required)):

In the present study, Riera-Borrull et al aimed to characterize the functional and transcriptional signatures of tumor infiltrating CD4+ T cells, in the context of distinct location or differential access to blood-delivered nutrients, in particular the glucose (mainly based on 2-NBDG staining pattern). The impact of glucose on Th1 or Th17 differentiation has been well studied. I have the following major concerns:

We thank the reviewer for their positive assessment of our study and we acknowledge their suggestions below for strengthening our findings.

1. The cytokine production profile was mainly measured by real-time PCR assay, and the key factors should also be measured at the protein level, either with FACS or ELISA or CBA.

We thank the Reviewer for this comment, and in our revision plan we will analyze the expression of key proteins as suggested.

2. The authors aimed to compare T cell characteristics with differential access to blood-delivered glucose, however, the authors did not provide any metabolic assay or parameters to prove their distinct metabolic activity.

We agree with the Reviewer that this point is worth addressing, and we have given considerable thought to how we can solve this question with the very small number (few hundreds) of effector TILs in the 2-NBDG (or Hoechst) Hi and Lo subsets we get after sorting. We think that perhaps our best option here could be to try to analyze metabolic parameters, such as their dependence on glycolysis versus mitochondrial respiration for ATP production, using SCENITH or a similar assay based on puromycin incorporation and flow cytometry, as this technique is suitable for small numbers of cells. We have also considered isolating whole TILs (without sorting into high and low blood accessibility subsets) and stimulating them in culture in normal or nutrient-depleted medium, and then probing their sensitivity to different metabolic inhibitors for ATP production. We plan to test this approach, as it has the advantage of allowing us to expand the cultures and obtaining more cells. However, it also has the caveat that TILs cultured in a different medium than they were *in vivo* might not necessarily exhibit equivalent properties to TILs originally residing in higher and lower blood accessibility regions.

3. Beyond the effector function, the availability of glucose or their metabolites certainly shape the differentiation status of tumor infiltrating T cells. In this regard, how about the stemness or exhaustion status of those 2-NBDG low or high T cells?

This is a very interesting point, as stemness and exhaustion of tumor-infiltrating T cells are relevant in tumor progression. We have analyzed the expression of PD-1 in Figure 7, where we show that 2-NBDG ^{Lo} and Hoechst ^{Lo} CD4 and CD8 T cells have lower surface expression of PD-1 than their 2-NBDG ^{Hi} and Hoechst ^{Hi} counterparts. Our RNA-seq data for exhaustion-associated markers TIM-3 (*Havcr2*) and LAG-3 show moderately higher expression of both in 2-NBDG ^{Lo} effector CD8 TILs than in 2-NBDG ^{Hi} ones, but fewer reads for them in 2-NBDG ^{Lo} than in 2-NBDG ^{Hi} effector CD4 TILs. Regarding the stemness factor TCF1 (encoded by *Tcf7*), our RNA-seq data show a very mild (~ 1.2-fold) increase in its expression in 2-NBDG ^{Lo} effector CD4 and CD8 TILs. Our revision plan will analyze mRNA and protein levels for these markers in 2-NBDG or Hoechst Hi/Lo CD4 and CD8 TILs.

4. What are the underlying mechanisms of differential CXCR3 expression pattern?

Our current thinking about how CXCR3 is more expressed in 2-NBDG ^{Lo} and Hoechst ^{Lo} effector TILs is based on our findings and the literature. One possibility is that it could result from the enhanced responsiveness to IFNs in low-accessibility TILs, as we see that CXCR3 is partially dependent on IFNAR signaling (Figure 4). In addition, it has been described that IFNγ is a potent inducer of CXCR3 (Nakajima C et al., (2002) *Eur. J. Immunol*. 32:1792). On the other hand, CXCR3 expression can be repressed by the cytokine TFGβ and STAT3-activating signals (Gunderson AJ et al., (2020) *Nature Comm*. 11:1749; Yue C et al., (2015) *Cancer Immunol. Res*. 3:864; Sun Q et al., (2023) *J. Exp. Med*. 220:20220686).

In our revision we will discuss these possibilities, and in terms of experiments, we plan to isolate different immune cell types, as well as tumor cells with low or high blood accessibility to analyze

their expression of cytokines (IFNs, TGFβ, IL-10 as a STAT3-activating cytokine) that could regulate CXCR3 expression in T cells.

5. How about the CD8+ T cells?

Our experiments in Figures 3 to 7 and in supplementary Figure S2 include a parallel analysis of CD4 and CD8 TILs. As for the new experiments we plan to address the reviewers' questions, they will also be done in CD4 as well as CD8 cells.

Reviewer #3 (Significance (Required)):

The conceptual novelty is limited given a lot of studies in this relevant field.

We would like to point out the relevant findings in our manuscript that support its novelty. First, our results uncover a remarkable efficiency of activated T cells to maintain glucose-dependent functions under very low glucose levels. Second, we identify markers of tumor-infiltrating, effector CD4 and CD8 T cells that differ between cells with higher and lower accessibility to blood, such as the expression of IFN-I response signatures, the chemokine receptor CXCR3, and PD-1. Thus, our results show that tumor-infiltrating effector T cells with reduced access to blood exhibit some characteristics of glucose-restricted cells, but also show characteristics of activated and potentially antitumor-capable cells. Previous works about tumor-infiltrating T cells had not identified specific features of effector TIL subsets distinguishable by their accessibility to blood, and our findings here offer an alternative scenario to the commonly held view that T cells in poorly perfused tumor regions are largely inactive due to nutrient insufficiency.

3. Description of the revisions that have already been incorporated in the transferred manuscript

Please insert a point-by-point reply describing the revisions that were <u>already carried out and included</u> in the transferred manuscript. If no revisions have been carried out yet, please leave this section empty.

No revisions have been carried out yet, but we have explained how we will approach them under each Reviewer's points in our revision plan.

4. Description of analyses that authors prefer not to carry out

Please include a point-by-point response explaining why some of the requested data or additional analyses <u>might not be necessary or cannot be provided within the scope of a revision</u>. This can be due to time or resource limitations or in case of disagreement about the necessity of such additional data given the scope of the study. Please leave empty if not applicable.

As discussed in our reply to Reviewer #2, we lack the resources and methodology to carry out 13C-glucose tracing. Also, as discussed in our reply to this reviewer, for tracing glucose to cytokine expression we would need to connect lower glucose availability with quantitative changes in posttranslational modifications of histones and transcription factors with glucose-derived metabolites. We respectfully think that these experiments, while interesting, would be out of our reach and perhaps would not contribute substantially to other points of the manuscript raised by the Reviewer, which we think are more relevant to address.

Dear Dr. López-Rodríguez,

Thank you for the transfer of your research manuscript from Review Commons to EMBO reports. I now went through your manuscript, the referee reports from Review Commons (attached again below) and your revision plan. The referees have several comments, concerns, and suggestions to improve the manuscript, indicating that a major revision of the manuscript is necessary to allow publication of the study.

Going through your revision plan, it seems that the referee points will be adequately addressed during revision. I thus invite you to revise your manuscript accordingly with the understanding that all concerns must be addressed in the revised manuscript and/or in a final detailed point-by-point response (as indicated in your revision plan). It will be important, though, to provide more mechanistic insight, performing the experiments proposed by the reviewers (in particular of referee #2) to improve the work.

Acceptance of your manuscript will depend on a positive outcome of another round of review using the same set of referees. It is EMBO reports policy to allow a single round of major revision only and acceptance of the manuscript will therefore depend on the completeness of your responses included in the next, final version of the manuscript.

Revised manuscripts should be submitted within three months of a request for revision. Please contact me to discuss the revision (also by video chat) if you have questions or comments regarding the revision, or should you need additional time.

When submitting your revised manuscript, please also carefully review the instructions that follow below.

PLEASE NOTE THAT upon resubmission revised manuscripts are subjected to an initial quality control prior to exposition to re-review. Upon failure in the initial quality control, the manuscripts are sent back to the authors, which may lead to delays. Frequent reasons for such a failure are the lack of the data availability section (please see below) and the presence of statistics based on n=2 (the authors are then asked to present scatter plots or provide more data points).

When submitting your revised manuscript, we will require:

1) a .docx formatted version of the final manuscript text (including legends for main figures, EV figures and tables), but without the figures included. Figure legends should be compiled at the end of the manuscript text.

2) individual production quality figure files as .eps, .tif, .jpg (one file per figure), of main figures (up to 8) and EV figures (up to 5). Please upload these as separate, individual files upon re-submission.

The Expanded View format, which will be displayed in the main HTML of the paper in a collapsible format, has replaced the Supplementary information. You can submit up to 5 images as Expanded View. Please follow the nomenclature Figure EV1, Figure EV2 etc. The figure legend for these should be included in the main manuscript document file in a section called Expanded View Figure Legends after the main Figure Legends section. Additional Supplementary material should be supplied as a single pdf file labeled Appendix. The Appendix should have page numbers and needs to include a table of content on the first page (with page numbers) and legends for all content. Please follow the nomenclature Appendix Figure Sx, Appendix Table Sx etc. throughout the text, and also label the figures and tables according to this nomenclature.

For more details, please refer to our guide to authors:
http://www.embopress.org/page/journal/14693178/authorguide#manuscriptpreparation

Please consult our guide for figure preparation:
http://wol-prod-cdn.literatumonline.com/pb-assets/embo-site/EMBOPress_Figure_Guidelines_061115-1561436025777.pdf

See also the guidelines for figure legend preparation:
https://www.embopress.org/page/journal/14693178/authorguide#figureformat

3) one final .docx formatted letter INCLUDING the reviewers' reports and your detailed point-by-point responses to their comments. As part of the EMBO Press transparent editorial process, the point-by-point response is part of the Review Process File (RPF), which will be published alongside your paper.

4) a complete author checklist, which you can download from our author guidelines (https://www.embopress.org/page/journal/14693178/authorguide). Please insert page numbers in the checklist to indicate where the requested information can be found in the manuscript. The completed author checklist will also be part of the RPF.

Please also follow our guidelines for the use of living organisms, and the respective reporting guidelines:
http://www.embopress.org/page/journal/14693178/authorguide#livingorganisms

5) that primary datasets produced in this study (e.g. RNA-seq, ChIP-seq, structural and array data) are deposited in an appropriate public database. If no primary datasets have been deposited, please also state this in a dedicated section (e.g. 'No primary datasets have been generated and deposited'), see below.

See also: http://embor.embopress.org/authorguide#datadeposition

Please remember to provide a reviewer password if the datasets are not yet public.

The accession numbers and database should be listed in a formal "Data Availability" section (placed after Materials & Methods) that follows the model below. This is now mandatory (like the COI statement). Please note that the Data Availability Section is restricted to new primary data that are part of this study. This section is mandatory. As indicated above, if no primary datasets have been deposited, please state this in this section

Data availability

The datasets produced in this study are available in the following databases:

- RNA-Seq data: Gene Expression Omnibus GSE46843 (https://www.ncbi.nlm.nih.gov/geo/query/acc.cgi?acc=GSE46843)
- [data type]: [name of the resource] [accession number/identifier/doi] ([URL or identifiers.org/DATABASE:ACCESSION])

*** Note - All links should resolve to a page where the data can be accessed. ***

Moreover, I have these editorial requests:

6) We now request the publication of original source data with the aim of making primary data more accessible and transparent to the reader. Our source data coordinator will contact you to discuss which figure panels we would need source data for and will also provide you with helpful tips on how to upload and organize the files.

7) Our journal encourages inclusion of *data citations in the reference list* to directly cite datasets that were re-used and obtained from public databases. Data citations in the article text are distinct from normal bibliographical citations and should directly link to the database records from which the data can be accessed. In the main text, data citations are formatted as follows: "Data ref: Smith et al, 2001" or "Data ref: NCBI Sequence Read Archive PRJNA342805, 2017". In the Reference list, data citations must be labeled with "[DATASET]". A data reference must provide the database name, accession number/identifiers and a resolvable link to the landing page from which the data can be accessed at the end of the reference. Further instructions are available at: http://www.embopress.org/page/journal/14693178/authorguide#referencesformat

8) Regarding data quantification and statistics, please make sure that the number "n" for how many independent experiments were performed, their nature (biological versus technical replicates), the bars and error bars (e.g. SEM, SD) and the test used to calculate p-values is indicated in the respective figure legends (also for potential EV figures and all those in the final Appendix). Please also check that all the p-values are explained in the legend, and that these fit to those shown in the figure. Please provide statistical testing where applicable. Please avoid the phrase 'independent experiment', but clearly state if these were biological or technical replicates. Please also indicate (e.g. with n.s.) if testing was performed, but the differences are not significant. In case n=2, please show the data as separate datapoints without error bars and statistics. See also: http://www.embopress.org/page/journal/14693178/authorguide#statisticalanalysis

If n<5, please show single datapoints for diagrams.

9) Please also note our reference format:
http://www.embopress.org/page/journal/14693178/authorguide#referencesformat

10) We updated our journal's competing interests policy in January 2022 and request authors to consider both actual and perceived competing interests. Please review the policy https://www.embopress.org/competing-interests and update your competing interests if necessary. Please name this section 'Disclosure and Competing Interests Statement' and put it after the Acknowledgements section.

11) We now use CRediT to specify the contributions of each author in the journal submission system. CRediT replaces the author contribution section. Please use the free text box to provide more detailed descriptions and do NOT provide an author contributions section in the revised manuscript text file. See also guide to authors:
https://www.embopress.org/page/journal/14693178/authorguide#authorshipguidelines

12) Please add scale bars of similar style and thickness to microscopic images, using clearly visible black or white bars (depending on the background). Please place these in the lower right corner of the images themselves. Please do not write on or near the bars in the image but define the size in the respective figure legend.

13) Please make sure that all the funding information is also entered into the online submission system and that it is complete and similar to the one in the acknowledgement section of the manuscript text file.

14) All Materials and Methods need to be described in the main text using our 'Structured Methods' format, which is required for all research articles. According to this format, the Materials and Methods section should include a Reagents and Tools Table (listing key reagents, experimental models, software and relevant equipment and including their sources and relevant identifiers), uploaded as separate file, followed by a Methods and Protocols section in which we encourage the authors to describe their methods using a step-by-step protocol format with bullet points, to facilitate the adoption of the methodologies across labs. More information on how to adhere to this format as well as downloadable templates (.doc or .xls) for the Reagents and Tools Table can be found in our author guidelines (section 'Structured Methods'):

https://www.embopress.org/page/journal/14693178/authorguide#manuscriptpreparation

15) Please order the manuscript sections like this, using these names:
Title page - Abstract - Keywords - Introduction - Results - Discussion - Methods - Data availability section - Acknowledgements (including funding information) - Disclosure and Competing Interests Statement - References - Figure legends - Expanded View Figure legends

We would also welcome the submission of cover suggestions, or motifs to be used by our Graphics Illustrator in designing a cover.

As part of the EMBO publication's Transparent Editorial Process, EMBO reports publishes online a Review Process File (RPF) to accompany accepted manuscripts. This File will be published in conjunction with your paper and will include the referee reports, your point-by-point response and all pertinent correspondence relating to the manuscript.

You are able to opt out of this by letting the editorial office know (emboreports@embo.org). If you do opt out, the Review Process File link will point to the following statement: "No Review Process File is available with this article, as the authors have chosen not to make the review process public in this case."

I look forward to seeing a revised version of your manuscript when it is ready. Please let me know if you have questions or comments regarding the revision.

Best,

Achim Breiling
Senior editor
EMBO reports

Referee #1:

The research article "Effector T cells in poorly perfused tumor regions exhibit a distinct signature of augmented IFN response and reduced PD-1 expression" by Riera-Borrull et al. investigates the glucose-dependent functions of Th17 and Th1 cells activated under very low glucose concentrations similar to those encountered in tumors. This research has the potential to significantly impact our understanding of T cell function in tumor microenvironments. Their main claim is that T lymphocytes can achieve substantial activation and maintain energy sufficiency under deficient levels of glucose. In addition, they also claim that effector TILS with very low accessibility to glucose (blood) express multiple glucose-dependent genes and markers associated with enhanced antitumor capacity.

The study is clear and easy to follow, significant in the field, and has well-described and, in principle, adequate experimental procedures. However, the authors need to perform additional experiments to fully support their claims.

Major comments:

- Figure 1. The authors start the experiments by placing polyclonally activated CD4 lymphocytes under basal (5 mM) or low glucose levels (0.3 mM) and studying the expression of hallmark Th17 and Th1 cytokines. They compared the expression of these cytokines using two conditions: 1) CD4 T cells isolated from LN and spleen activated with anti-CD3 and anti-CD28 and differentiated towards Th17 (TGF-β and IL-6) with low or high glucose concentrations. 2) Activation of lymphocytes from LN in the presence of IL-2 for 5 days, followed by the re-activation with anti-CD3 and CD28 and polarization toward Th17. The experimental design is incorrect. Cells from condition 1) are correctly termed 1ry Th17, but the cells so-called 2ry Th17 are not Th17. In order to really compare the two types of Th17, a condition where cells are polarized twice toward Th17. Can Th17 cells, freshly activated and differentiated in a normal glucose medium,< produce the same amount of cytokines as cells in a low

glucose medium? Are the cells from condition 1 naïve or only isolated CD4 T cells?
- Figure 2. The authors use a very nice approach, injecting anti-CD3 antibodies to activate T cells in vivo. Nevertheless, they take total CD4 T cells without looking for the activated ones, for example, by sorting using activation markers such as CD25, CD69, or even CD62L and CD44 to take the Teff cells. In addition, the Isotype IgG control is missing, and the control t=0 included in the figures is insufficient.
- Figure 3. The title is misleading as the authors study both CD8 and CD4. The gating strategy described in Sup2 is insufficient. What is the expression of other immune checkpoint genes such as TIM-3, PD-1, or LAG-3? Can authors complement Figure 3D with cytometry to know the protein levels?
The same with Figure 6, can the authors show protein levels (IFNg, CTLA4, Foxp3)
- Figure 7. To determine the functionality of the CD4 and CD8 TILS, the authors need to transfer those cells to congenic (CD45.1) tumor-bearing mice and see if they can conserve the described phenotype.

- Are the claims and the conclusions supported by the data or do they require additional experiments or analyses to support them?

The conclusions are supported, despite the needed experiments and some flaws in the controls. However, further investigation would be beneficial.

- Please request additional experiments only if they are essential for the conclusions. Alternatively, ask the authors to qualify their claims as preliminary or speculative, or to remove them altogether.

See above

- Are the suggested experiments realistic in terms of time and resources? It would help if you could add an estimated time investment for substantial experiments.

The suggested experiments are easy to perform, and maybe the authors already have some of them. I would say that three months would be enough.

- Are the data and the methods presented in such a way that they can be reproduced?

Yes, they are well described and can be reproduced.

- Are the experiments adequately replicated and statistical analysis adequate?

Yes

Significance (Required):

Provide contextual information to readers (editors and researchers) about the novelty of the study, its value for the field and the communities that might be interested.

The study is novel and studies an important subject: the conditions encountered by TILS, poor glucose access, and, therefore, metabolic pressure. The subject is important, and the approach is adequate. There are some flaws in the experimental design, mostly in the in vitro/ex vivo experiments, but they can be resolved by the authors.

The following aspects are important:

- *Audience:* basic and translational research in cancer but also applicable to other diseases such as autoimmune disease where T cell metabolism is pivotal.
- Please define your field of expertise with a few keywords to help the authors contextualize your point of view. Indicate if there are any parts of the paper that you do not have sufficient expertise to evaluate.

Immunology of T cells, cancer, immunotherapy

Referee #2:

Summary

In this manuscript, Riera-Borrull et al. first analyzed how glucose restriction might affect T lymphocyte function in vitro and found that T cells were capable of expressing certain cytokines such as IFNy and IL17A after stimulation, even with limited glucose availability. They then conducted in vivo experiments to assess expression profiles in tumor infiltrating T cells (TILs) in vivo with differential uptake of the glucose analog (i.e. 2-NBDG) and their accessibility to blood. Their findings revealed that 2-NBDGlo

cells (i.e., presumably cells with less glucose uptake) exhibited a pronounced type I interferon signature compared to the 2-NBDGhi cells. Additionally, the uptake of 2-NBDG correlated with accessibility of TILs to blood vessels. The authors concluded that TILs with reduced access to blood upregulate ACSS2 to produce cellular acetyl-CoA, thereby sustaining gene expression, including ISGs. Lastly, the author observed that limited access to blood increased CXCR3 expression but decreased PD-1 expression in effector CD4 and CD8 TILs.

Major comments:

Overall, the story is highly descriptive. For example, cells consuming more glucose exhibit the same cellular ATP levels as the cells with limited glucose (Figures S1B and C). Why is this the case? Do these cells have the same mitochondrial respiration, TCA cycle activity and etc? Additionally, although glucose restriction exerts similar effects on ATP levels in Th1 and Th17 cells ex vivo, the cells display different cytokine expression changes in response to varying glucose concentrations, suggesting that glucose-derived ATP may not be the sole factor impacting cytokine expression in those cells. Therefore, additional experiments such as seahorse and 13C-labeled glucose tracing are required to further characterize the role of glucose in mediating cytokine expression in those cells. Moreover, the cytokine expression in stimulated T cells can be dynamic. Thus, a few more time points would be helpful to more precisely assess how glucose availability affects cytokine expression.

The authors identified enhanced type I interferon signaling in 2-NBDGlo cells but did not investigate this further. Questions remain, such as the source of IFN (tumors, T cells or other cells) and why 2-NBDGlo cells more responsive to IFN. The lack of explanations or mechanistic studies renders these results less compelling.

The authors stated "2-NBDG uptake is thought to correlate with the metabolic activity of T cells(37), and our results below indicate that TILs with poor 2-NBDG uptake resemble T cells under glucose limitation" (Page 10, lines 200-201). However, the author should validate that 2-NBDGlo cells indeed have lower glucose concentration compared to the 2-NBDGhi cells. Otherwise, conclusions based on this assumption are not convincing. For instance, ISGs were reduced in T cells in low glucose medium but augmented in 2-NBDGlo cells, contradicting the statement that "our results below indicate that TILs with poor 2-NBDG uptake resemble T cells under glucose limitation"!

Some conclusions lack solid supporting evidence. Although the authors cited previous studies, certain experiments are necessary to substantiate their conclusions. For example, the authors concluded that 2-NBDGlo cells upregulate ACSS2 to produce acetyl-CoA for gene expression without actually measuring acetyl-CoA in those cells. We do not know whether: 1) 2-NBDGlo cells have low glucose; 2) 2-NBDGlo cells have low acetyl-CoA (Acetyl-CoA can be generated through other routes, such as fatty acid oxidation); 3) Increased ACSS2 necessarily leads to more acetyl-CoA production in those cells.

ACSSi experiments are also unconvincing, as the authors do not provide any data to demonstrate that the inhibitors function properly and that the downstream effects are not due to off-target effects. Moreover, it is possible that TILs with different accessibility to blood take up different amounts of the ACSSi, which could affect the comparison of cytokine expression in Hoechsthi/lo cells.

Significance (Required):

The major issue of this study is the lack of mechanistic insights, which significantly diminishes both impact and novelty of the findings. Much of the data is descriptive. The findings are not particularly exciting, also partially due to the absence of mechanisms. With more mechanistic insights and solid characterizations, the study could be of interest in the fields of immunometabolism and tumor immunotherapy.

My expertise includes metabolism and cell signaling. I find that the metabolic studies in this manuscript are disappointing and require further investigations.

Referee #3:

In the present study, Riera-Borrull et al aimed to characterize the functional and transcriptional signatures of tumor infiltrating CD4+ T cells, in the context of distinct location or differential access to blood-delivered nutrients, in particular the glucose (mainly based on 2-NBDG staining pattern). The impact of glucose on Th1 or Th17 differentiation has been well studied. I have the following major concerns:

1. The cytokine production profile was mainly measured by real-time PCR assay, and the key factors should also be measured at the protein level, either with FACS or ELISA or CBA.
2. The authors aimed to compare T cell characteristics with differential access to blood-delivered glucose, however, the authors did not provide any metabolic assay or parameters to prove their distinct metabolic activity.
3. Beyond the effector function, the availability of glucose or their metabolites certainly shape the differentiation status of tumor infiltrating T cells. In this regard, how about the stemness or exhaustion status of those 2-NBDG low or high T cells?
4. What are the underlying mechanisms of differential CXCR3 expression pattern?

5. How about the CD8+ T cells?

Significance (Required):

The conceptual novelty is limited given a lot of studies in this relevant field.

Full Revision

Manuscript number: EMBOR-2024-60965V1-T | [RC-2024-02596] [REV]
Corresponding author(s): Cristina López-Rodríguez

Authors' note: We submitted our manuscript originally to Review Commons, and once we received the reviewers' comments, we proposed a Revision Plan and transferred it to EMBO Reports. At that point we had not done any experimental revision yet. We are now submitting to EMBO Reports a fully revised manuscript with new sets of experimental results and figures.

In our reply to the reviewers, we have used the same template from Review Commons, and maintained the text of our *Revision Plan in lighter blue italics*, followed by the new, point-by-point reply with the description of the revisions done, in darker blue. We think that having both texts provides a fuller perspective on how we have revised our manuscript, the rationale behind our experiments, and the obstacles encountered. In order to facilitate reading, we have added in the text of our previous *Revision Plan* the new numbering of figures in the revised manuscript. The reviewers' comments are in *crimson italics*.

1. General Statements [optional]

This section is optional. Insert here any general statements you wish to make about the goal of the study or about the reviews.

Summary of the goal, rationale, and main findings of our study.

A main goal of our work was to analyze the characteristics of effector T lymphocytes in tumor regions with substantially restricted access to blood. Previous works have studied metabolic and nutrient utilization characteristics of T cells in tumors, but had not distinguished between cells with higher and lower access to blood-transported nutrients. Addressing this question is relevant to better define the heterogeneity of T lymphocytes in the tumor and to understand their potential in response to immunotherapeutic approaches.

Effector T cells are avid nutrient consumers, and in the tumor microenvironment they compete for nutrients with cancer cells and other immune cells. This competition can be more intense in poorly perfused regions, where T cells may not obtain enough nutrients to sustain antitumor function; so it was generally thought that the anti-tumor capability of T cells in these regions will be largely limited due to nutrient insufficiency. However, previous works showing that tumor-infiltrating T lymphocytes (TILs) respond to stimulation with anti-checkpoint antibodies led us to think that T cells might possibly be more competent than commonly thought in metabolically challenging microenvironments. At present, it is not known to what extent T cells in tumor regions with poor access to blood really are impaired or dysfunctional, and which characteristics distinguish them from effector T cells in well irrigated tumor areas. Here we have addressed these questions by characterizing effector CD4 and CD8 T lymphocytes isolated from highly and poorly accessible tumor regions, where they may face different metabolic conditions, and compared them with

tumor-inexperienced CD4 T cells from lymph nodes, as well as TILs activated *ex vivo* in well-defined glucose-restricted conditions.

In the first part of our manuscript, we used *in vitro* assays to ask how tumor-inexperienced CD4 T cells preactivated in different ways would respond to stimulation via T cell receptor in glucose-poor conditions. We chose glucose restriction because activated effector T cells are heavy glucose consumers and much dependent on glucose to maintain their function. We found that CD4 T cells activated as T helper (Th) 17 or Th1, known to be highly dependent on glucose, were capable of maintaining an activated state for days and even induce diverse glucose-dependent cytokines under severe glucose restriction. Notably, T cells activated in low glucose, down to levels reported in tumors and less than 20-50 times lower than in blood, still used glucose as their main fuel for ATP production, which altogether suggested that they can display substantial functionality with little glucose.

These results were intriguing, but glucose is just one nutrient, and so we asked which characteristics could be expected in T cells *in vivo* in a metabolically challenging milieu with reduced access to blood-transported nutrients. To address this, we reasoned that tumor-infiltrating T cells (TILs) more likely to be subjected to a general nutrient deficiency would be found in tumor regions poorly perfused by blood, and used the uptake of intravenously injected fluorescent probes by T cells as an indicator of their accessibility to blood-transported nutrients. This approach allowed us to isolate and characterize different populations of effector CD4 and CD8 TILs *in vivo*.

We have found that effector T lymphocytes infiltrating tumors express different gene signatures in regions with different accessibility to blood, and can maintain specific glucose-dependent responses even in poorly perfused tumor regions. The novelty of our work is the identification of specific features that distinguish effector TIL subsets by their accessibility to blood, and the finding that effector CD4 and CD8 T cells in poorly accessible tumor regions do not just show some features expected in nutrient-restricted cells, but also exhibit characteristics of activated T cells with potentially enhanced antitumor capacity. Our results open new questions for exploring the functional heterogeneity and compartmentalization of TILs in tumor regions with higher or lower accessibility to blood.

We are grateful to the reviewers for their insight, critiques, and questions, since by addressing them, we have uncovered relevant new findings, reassessed some of our previous results from new perspectives, and strengthened the key conclusions of our work. This revision has taken considerable time, in part because we had to set up and troubleshoot new methodology, circumvent failed approaches, and find alternative ones. Our revised manuscript includes new results that address the points raised by the reviewers, of which we would highlight that: i) TILs in poorly accessible tumor regions have reduced biosynthetic activity but still utilize glucose as a major energy source; ii) they are intrinsically as competent as TILs from well-perfused regions to produce IFNγ and lytic granule proteins, present an IFN-I-response signature, likely induced by myeloid cells in their microenvironment expressing IFNα and IFNβ1, and can respond to

increases in available glucose by enhancing their output of antitumor cytokines; and iii) however, they express significantly lower levels of T regulatory and exhaustion-associated proteins, which suggests that they might be less vulnerable to suppressor mechanisms from the tumor microenvironment than cells in regions with better blood accessibility. Accordingly, we have updated the title, abstract and all sections of the main text; included three full new figures (new Figs, 5, 6, 7), and expanded or rearranged previous ones (in new Figs. 1, 3, new Figs. EV1, 2, 3, 4, 5 and new Appendix Fig. S1); and made a new graphical abstract. We have also updated the information in former Figure 7 with additional results from a new experiment, and it is now shown in this letter as Fig. R2 in the reply to Reviewer 3. All these changes are described in the point-by-point reply to the reviewers.

In summary, we have addressed experimentally most of the reviewers' questions, and as result of the revision we have obtained significant new results *in vivo* and *ex vivo* that we feel enhance the novelty and relevance of our work. We also found obstacles in trying to address some of the questions raised by the reviewers, but have done alternative experiments that at least provide new information to help answer the reviewers' questions. This is all discussed in detail in our point-by-point reply. We believe that our improved manuscript has clearly gained in relevance to be positively considered by the reviewers and the editor.

2. Point-by-point description of the revisions

This section is mandatory. Please insert a point-by-point reply describing the revisions that were <u>already carried out and included</u> in the transferred manuscript.

We appreciate the work done by the reviewers and their thoughtful comments and recommendations for improvement. By addressing these points, we have uncovered new original findings, which we have included in the revised manuscript, and reassessed some of our previous results to provide a clearer perspective of their significance. Overall, we believe that our manuscript has gained a stronger experimental base and greater significance, and we hope that the editor and reviewers will now find it suitable for publication in EMBO Reports.

Below each of the reviewers' points, we have included *our initial Revision Plan*, as it discusses relevant points about specific reviewers' comments, followed by the detailed point-by-point reply describing the revisions done and the corresponding modifications in the manuscript to incorporate them.

Reviewer #1 (Evidence, reproducibility and clarity (Required)):

The research article "Effector T cells in poorly perfused tumor regions exhibit a distinct signature of augmented IFN response and reduced PD-1 expression" by Riera-Borrull et al. investigates the glucose-dependent functions of Th17 and Th1 cells activated under very low glucose concentrations similar to those encountered in tumors. This research has the potential to significantly impact our understanding of T cell function in tumor microenvironments. Their main claim is that T lymphocytes can achieve substantial activation and maintain energy sufficiency under deficient levels of glucose. In addition, they also claim that effector TILS with very low accessibility to glucose (blood) express multiple glucose-dependent genes and markers associated with enhanced antitumor capacity.

The study is clear and easy to follow, significant in the field, and has well-described and, in principle, adequate experimental procedures. However, the authors need to perform additional experiments to fully support their claims.

We thank the Reviewer for their appreciation of our work and their positive overall assessment. We are also grateful for the insightful comments and helpful suggestions.

Major comments:

-Figure 1. The authors start the experiments by placing polyclonally activated CD4 lymphocytes under basal (5 mM) or low glucose levels (0.3 mM) and studying the expression of hallmark Th17 and Th1 cytokines. They compared the expression of these cytokines using two conditions: 1) CD4 T cells isolated from LN and spleen activated with anti-CD3 and anti-CD28 and differentiated towards Th17 (TGF-β and IL-6) with low or high glucose concentrations. 2) Activation of lymphocytes from LN in the presence of IL-2 for 5 days, followed by the re-activation with anti-CD3 and CD28 and polarization toward Th17. The experimental design is incorrect. Cells from condition 1) are correctly termed 1ry Th17, but the cells so-called 2ry Th17 are not Th17. In order to really compare the two types of Th17, a condition where cells are polarized twice toward Th17. Can Th17 cells, freshly activated and differentiated in a normal glucose medium, produce the same amount of cytokines as cells in a low glucose medium? Are the cells from condition 1 naïve or only isolated CD4 T cells?

***Initial revision plan**:*

We thank the Referee for the correction about our having named the cells 1ry and 2ry Th17 cells. This will be corrected in the revised version of the manuscript and preactivated T cells restimulated with TGF-β and IL-6 will not be referred to as 2ry Th17. Also, cells from condition 1 were freshly isolated from LNs, but not naïve. This will be made clear in the revised manuscript.

Regarding the question of whether freshly activated Th17 cells would produce the same amounts of cytokines in normal or low glucose medium, our results show they do at the mRNA level.

Measurement of cytokine protein levels produced by these cultures in 5 or 0.3 mM glucose will be included in our revision plan.

Point-by-point reply description of the revisions done:

The term "secondary Th17 cells" has been eliminated and these cells are now referred to as "preactivated T cells restimulated as Th17" or with an equivalent description.

We have analyzed cytokine mRNA and protein production in naïve (CD62L $^{+}$ CD44 ^{neg}) CD4 T cells stimulated as Th17 cells (new Fig. 1A and Fig. EV1A), and show that they express IL-17A mRNA comparably when activated in 0.3 or 5 mM glucose, but IL-17A protein was better induced in 5 mM (new Fig. 1A). By contrast to preactivated CD4 T cells, we neither detected IL-22 and IFNγ mRNA reliably in naïve ones after 48 hours of stimulation, nor significant accumulation of either cytokine in the culture supernatant (new Fig. 1A). In the same experiments we found that TNFα was better produced in 5 mM than 0.3 mM glucose, whereas IL-2 production was not decreased in low glucose (new Fig. EV1A).

We have now moved the part of former Figure 1A that showed this experiment in freshly isolated, but not naïve purified CD4 T cells, to Fig. EV1B.

-Figure 2. The authors use a very nice approach, injecting anti-CD3 antibodies to activate T cells in vivo. Nevertheless, they take total CD4 T cells without looking for the activated ones, for example, by sorting using activation markers such as CD25, CD69, or even CD62L and CD44 to take the Teff cells. In addition, the Isotype IgG control is missing, and the control t=0 included in the figures is insufficient.

Initial revision plan:

We agree with the referee that an analysis of sorted effector T cells activated in vivo would be needed. In this regard, we think that the results in Figure 2 (new Fig. EV1, C and D) might be a bit redundant with those in Figure 1 and Figure S1C (new Fig. 1J) for the analysis of ATP sources, as the three figures show that T cells stimulated in culture in low glucose can still use glucose as a main fuel for ATP production and also increase their dependence on mitochondrial respiration. Likewise, both Figures 1 and 2 show that expression of several cytokines is affected to a greater or lesser extent by low glucose depending on how T cells were stimulated. As the next figures and the main focus of the manuscript are on tumor-infiltrating T cells (TILs), we propose to do our revision with TILs sorted as effector, which already show characteristics of activated T cells.

Point-by-point reply description of the revisions done:

In order to address the glucose dependence of *in vivo*-activated T cells, we have used tumor-infiltrating T cells (TILs) isolated from LLC tumors and FACS-sorted as effector memory (CD62L ^{neg} CD44 $^{+}$) CD4 T cells, excluding naïve and central memory T cells. Effector memory

(abbreviated as effector) represent the large majority of TILs. We stimulated them *ex vivo* with anti-CD3 and anti-CD28 plus IL-2 in normal (5 mM) or low-glucose (0.3 mM) medium, and found that, similarly to other CD4 T cell sources used above in Fig. 1 and Fig. EV1, effector TILs could induce IL-17A and IFNγ mRNA when stimulated in 0.3 mM glucose (new Fig. 3A). However, this experiment also showed that TILs stimulated in low glucose had more mRNA and a higher mRNA-to-protein ratio than cells in 5 mM glucose, but produced less protein than those in 5 mM glucose over 48 hours (new Fig. 3A). TILs also produced IL-2 and TNFα better in 5 mM than 0.3 mM glucose (new Fig. EV2F). Nonetheless, TILs in low-glucose medium could still produce substantial amounts of cytokine proteins, similarly to what we had seen with lymph node T cells in new Fig. 1 and Fig. EV1.

Since mRNA and protein levels for IL-17A and IFNγ were measured at a single time point (48 h), their differences might reflect their respective expression kinetics, but could as well suggest that TILs under glucose limitation had reduced protein translation capacity, which we confirmed experimentally (new Fig. 7 see comment in the next paragraph and also in our reply to Reviewer #3), and would also be consistent with the reduced mTORC1 activity we saw in LN T cells activated in low glucose (now shown as Fig. 2A), Regarding our former Figure 2 in which we had not sorted effector T cells after *in vivo* anti-CD3 injection, this result could reflect the response of a mixture of heterogeneously activated cells. Nonetheless, we have kept these data in the manuscript, as we think they are useful, but moved them to Fig. EV1, C and D.

In addition, we have used the SCENITH assay to assess the dependence of effector CD4 and CD8 TILs on glycolysis and mitochondrial respiration, with respect to their better or poorer accessibility to blood. Please see our reply to Reviewer #3 for a detailed description of the protein translation and metabolic analysis in TILs using the SCENITH methodology (manuscript Refs. Argüello et al, 2020; Lopes et al, 2021). We found that effector CD4 and CD8 TILs with greater blood accessibility *in vivo* were more metabolically active than less accessible ones, as shown by their significantly higher protein translation rate. But then, we observed that TILs in both high and low blood accessibility compartments were largely dependent on glucose rather than mitochondrial respiration for ATP production. This result is shown in new Fig. 7. Altogether, these findings support the conclusion that T cells activated from naïve, or preactivated and effector TIL populations, use glucose, even when present at very low concentrations, as a main fuel for ATP production, and to produce hallmark glucose-dependent cytokines such as IFNγ or IL-17.

-Figure 3. The title is misleading as the authors study both CD8 and CD4. The gating strategy described in Sup2 is insufficient. What is the expression of other immune checkpoint genes such as TIM-3, PD-1, or LAG-3? Can authors complement Figure 3D with cytometry to know the protein levels? The same with Figure 6, can the authors show protein levels (IFNγ, CTLA4, Foxp3)

Initial revision plan:

We see the point of the Reviewer with the figure title, and we will change it to reflect that the figure shows results in CD4 and CD8 cells. Likewise, we will include the complete stepwise gating strategy used for the sorting of 2-NBDG ^{Hi} and ^{Lo} cells. Regarding the expression of TIM-3, PD-1 and LAG-3, PD-1 was analyzed in Figure 7, where we show that 2-NBDG ^{Lo} and Hoechst ^{Lo} CD4 and CD8 T cells have lower surface expression of PD-1 than their 2-NBDG ^{Hi} and Hoechst ^{Hi} counterparts. For TIM-3 and LAG-3, our RNA-seq data show moderately higher expression of TIM-3 (Havcr2) and LAG-3 in 2-NBDG ^{Lo} effector CD8 TILs than in 2-NBDG ^{Hi} ones, but fewer reads for both in 2-NBDG ^{Lo} than in 2-NBDG ^{Hi} effector CD4 TILs. Our revision plan will analyze protein levels for these markers in Hi/Lo CD4 and CD8 TILs. Likewise, we plan to analyze the expression of IFNγ, CTLA4, and Foxp3 proteins in the experiments of Figure 6 (new Fig. EV5).

Point-by-point reply description of the revisions done:

The title of new Fig. 3 has been changed, and a complete gating strategy that includes the step-by-step flow cytometry analysis sequence is now shown in new Fig. EV2, A, C, D. We have also analyzed the expression of immune checkpoint and inhibitory molecules (surface and intracellular protein) in effector TILs in high and poor blood-accessibility regions. These experiments were done with cells labeled with Hoechst *in vivo*, but not with 2-NBDG because the permeabilization needed for intracellular protein staining caused the loss of the 2-NBDG staining.

In our original version we showed that TILs with better access to intravenously injected Hoechst and 2-NBDG had higher expression of PD-1 (CD4 and CD8), and now we have extended these results to include that TILs with higher Hoechst uptake, besides expressing more PD-1, also express more FOXP3 (CD4), CTLA4 (CD4), LAG3 (CD4 and CD8), and TIM3 (CD8) proteins, shown as new Fig, 3, B and C. Our new results with the metabolic activity assays (SCENITH puromycin incorporation assays shown in new Fig. 7) and the protein analysis of PD-1 and diverse Treg and exhaustion-related markers indicate that effector TILs in tumor regions with higher blood accessibility are metabolically more active than low-accessibility TILs, but also express significantly higher levels of T regulatory and exhaustion-associated proteins, which suggests that they might be more vulnerable to suppressor mechanisms from the tumor microenvironment. We found it initially surprising that TILs more accessible to blood expressed more of Treg and exhaustion-associated markers than those in poorly perfused regions. But then, while setting up the puromycin incorporation assay for SCENITH, we found an article that analyzed how mTOR activity and protein translation rate correlated with activation and exhaustion features in TILs, and whose conclusions are complementary to our new findings (manuscript Ref. De Ponte Conti et al, 2021). The authors identified TILs with higher or lower protein translation capacity by their ability to incorporate puromycin *in vivo*, and showed that CD4 TILs with higher biosynthetic capacity contained higher proportions of Treg cells by FOXP3 and CTLA4 protein expression, and that CD8 TILs with higher translation capacity also expressed more PD-1 and TIM3 surface proteins. These findings recall our own, which show that TILs in regions more accessible to blood (higher *in vivo* uptake of Hoechst 33342), are more metabolically active and have higher expression of Treg, checkpoint and exhaustion-associated proteins. That complementary results were obtained by De Ponte Conti and us through independent approaches and using different

strategies and tumor models to analyze TILs, indicates that poorly perfused tumor regions could contain a previously unappreciated reserve of functionally competent effector T cells, in contrast with areas better irrigated by blood, where TILs might face stronger pressure from inhibitory (Treg, immune checkpoints) and exhaustion-inducing mechanisms.

With regard to IFNγ protein expression, CD4 and CD8 effector TILs in both high and low accessibility compartments rapidly produced IFNγ upon a short (4 hours) stimulation *ex vivo* with PMA plus ionomycin (new Fig. 3D). Intriguingly, we observed a mildly higher expression of IFNγ in lower accessibility TILs, and this difference was detectable also in unstimulated cells, which suggests that they might have been producing IFNγ *in vivo* while in the tumor. Although we are not sure of why this difference, it is possible that cells isolated from poorly perfused tumor regions respond more strongly when placed in a richer medium. In any case, this result suggests that TILs in low-accessibility tumor regions are as intrinsically capable of rapid response to stimulation as those with better accessibility to blood. Nonetheless, as shown by our longer-term (48 hours) stimulation experiments in new Fig. 3A, prolonged glucose restriction would be expected to decrease their cytokine output. In new Fig. 3D we also analyzed the expression of granzyme B and the capacity to release lytic granules (detected by the increase surface staining of CD107a) in effector CD8 TILs, and found comparable responses in cells with low and high accessibility. Altogether, our new findings suggest that TILs in poorly or well-perfused tumor regions have comparable intrinsic effector capability, but their actual activity *in vivo* could differ because they express different levels of immunomodulatory receptors (PD-1, CTLA4, TIM3, LAG3) to interact with other cells in their vicinity, and might also be exposed to different microenvironments, for instance higher expression of IFN-I by myeloid cells in low accessibility regions (please see new Fig. 5, in reply to Reviewer #2).

In our previous version of this manuscript, we had analyzed the expression of IFNγ, FOXP3, and CTLA4 in TILs only by mRNA but not protein. Our data show that the expression of these mRNAs in TILs sorted as high- or low-accessibility varies depending on whether we used 2-NBDG or Hoechst. *In vivo*, 2-NBDG ^{Lo} CD4 effector TILs had higher expression of FOXP3 and CTLA4 mRNA than 2-NBDG ^{Hi} cells. However, differences in FOXP3 and CTLA4 mRNA levels between Hoechst ^{Hi} and ^{Lo} CD4 TILs were less consistent between experiments (new Fig. EV3A shows a compilation of previous plus additional sorting assays with 2-NBDG or Hoechst). Our results for FOXP3 and CTLA4 with 2-NBDG coincided with recent work that showed that FOXP3 $^{+}$ Treg cells isolated from mouse tumors exhibit lower intrinsic 2-NBDG uptake capacity when cultured *ex vivo* than non-Treg cells (manuscript Ref. Watson et al, 2021). We also found that CD4 TILs in the 2-NBDG ^{Hi} fraction expressed more IFNγ mRNA than 2-NBDG ^{Lo} cells, but not between Hoechst ^{Hi} vs ^{Lo}, whereas for CD8 TILs it was Hoechst ^{Hi} cells that expressed more IFNγ mRNA than Hoechst ^{Lo} cells (new Fig. EV3A). In our revised manuscript we discuss that whereas Hoechst uptake mainly depends on accessibility to blood, the uptake of 2-NBDG *in vivo* might depend on both TILs accessibility to blood and their polarization state, in line with results from the Delgoffe laboratory (manuscript Ref. Watson et al, 2021), who showed that Treg TILs cultured *ex vivo* had lower cell-intrinsic 2-NBDG uptake capacity than non-Treg TILs. In sum, and in view of our new, extended protein and mRNA analyses with TILs *in vivo* and *ex vivo*, and complementary findings

in an independent publication (manuscript Ref. De Ponte Conti et al, 2021), we favor the interpretation that FOXP3 and CTLA4 protein are better indicators than their mRNA levels of the presence of more Tregs among TILs with higher accessibility to blood.

Regarding the analysis of IFNγ, CTLA4 and FOXP3 protein in previous Figure 6, this experiment tested how the inhibition of ACSS2 *in vivo* affected the expression of diverse genes in TILs in tumor regions with better and poorer accessibility. As Reviewer #2 had several questions about the selectivity and *in vivo* effect of the ACSS2 inhibitor used (ACSS2i/VY-3-249), we decided to repeat the experiment with a chemically independent, recently developed ACSS2 inhibitor (VY-3-135) (manuscript Ref. Miller et al, 2023). Our new results showed that VY-3-135 significantly inhibited the growth of LLC tumors (new Fig. EV5G), indicating its antitumor effectiveness in agreement with published work on other mouse tumor models (manuscript Ref. Miller et al, 2023). But then, VY-3-135 affected IFN-I response genes differently from VY-3-249 in our original experiment (new Fig. EV5, E and H). Whereas VY-3-249 had attenuated the expression of CXCL10 and IFIT1 mRNAs in TILs, VY-3-135 did not inhibit them. Miller et al., had shown that a 7-day regime of VY-3-135, longer than our 3 doses, enhanced the expression of several IFN-I response genes including *Ifit1* and *Cxcl10*, in TILs from Brpkp110 mouse mammary tumors, and also showed that a much longer treatment (28 days) enhanced the protein expression of IFNγ (CD4) and granzyme B (CD8) in TILs. In our experiment, VY-3-135 did not cause the mild inhibition of IFNγ mRNA expression that we had seen with ACSS2i/VY-3-249, but neither did it increase it. Finally, Miller et al., showed that ACSS2-deficient immune cells were as competent as wild-type ones in antitumor capacity, and concluded that the overall antitumor effect of the VY-3-135 was attributable to the inhibition of ACSS2 in tumor cells, which freed available acetate to be used by TILs.

The results by Miller et al., differed from a previous work (manuscript Ref. Qiu et al, 2019) who showed that reducing ACSS2 expression by shRNA knockdown in OT-I TCR transgenic CD8 T cells made them poorer IFNγ producers and weaker tumor controllers when infiltrating EL4-OVA subcutaneous tumors *in vivo*. The differing results between Miller et al. and Qiu et al. about the role of ACSS2 in the antitumor activity of TILs could be due to differences in the degree of inhibition of ACSS2 in their experimental systems, and the balance of acetate (the substrate for ACSS2) versus other metabolites in the TIL microenvironment. In this regard, as we discuss in our revised manuscript, work by the Hess laboratory showed that acetate can downregulate the expression of ACSS2 itself, and have pro- and anti-inflammatory effects (such as enhancing or inhibiting the expression of IFNγ) depending on the activation stage of the T cell (manuscript Ref. Balmer et al, 2020).

Seeing that these previous works reported different results as to the possible dependence of TILs on ACSS2, and that the ACSS2 inhibitors in our experiments *in vivo* had different, and relatively modest, effects on gene expression in TILs with better or poorer blood accessibility, we tested them directly in effector CD4 TILs activated *ex vivo* with anti-CD3 and anti-CD28 plus IFNα and IFNβ1. Both inhibitors were similarly effective at reducing the expression of IFNγ and CXCL10 mRNA in CD4 effector TILs, but ACSS2i/VY-3-249 caused a better inhibition of IFIT1 induction

(new Fig. 6A). Induction of STAT1 was mildly inhibited by ACSS2i/VY-3-249 and not by VY-3-135 (new Fig. 6A). Analysis of cytokine proteins showed that both ACSS2 inhibitors reduced the production of IFNγ and IL-17A, but only ACSS2i/VY-3-249 was moderately inhibitory for TNFα and IL-2 (new Fig. 6B). Therefore, our results indicate that ACSS2 can contribute to the expression of cytokines and several interferon-I response genes in effector TILs activated via T cell receptor and type I IFN stimulation. However, of the two inhibitors only ACSS2i/VY-3-249 showed a similar effect *in vivo* and *ex vivo* on TILs, whereas VY-3-135 did not inhibit TILs, and yet it significantly reduced tumor growth. As we discuss in our revised manuscript, these differences *in vivo* might involve indirect effects from other cells in the tumor microenvironment that could be variably sensitive to the inhibitors, or differences in the pharmacokinetics and metabolizing of these drugs *in vivo*.

-Figure 7. To determine the functionality of the CD4 and CD8 TILS, the authors need to transfer those cells to congenic (CD45.1) tumor-bearing mice and see if they can conserve the described phenotype.

Initial revision plan:

We agree with the Reviewer and appreciate this suggestion. However, a substantial limitation that we will find in this approach is the very small number of effector TILs in the 2-NBDG (or Hoechst) Hi and Lo subsets we can get after sorting. This number is in the hundreds of cells of each subset per tumor, far below to what we would need for adoptive transfer assays. Nonetheless, we think that even with few cells we could try some experiments to test their functionality and infer their antitumor potential better than by only analyzing gene expression. For instance, testing CD107a degranulation as an indicator of cytotoxic capacity, which would also complement our protein expression analyses in the previous point. We plan to carry out these assays in our revision plan. In addition, we plan to expand TILs in culture in normal or nutrient-depleted medium, which we hope would yield sufficient cells for testing their anti-tumor capacity in adoptive transfer assays. One potential problem with this experiment though is that TILs expanded ex vivo might adapt to the new culture conditions and lose part of the original characteristics they had in the tumor. Nonetheless, we think it is worth trying it and see how they compare in the expression of relevant ISGs and activation and polarization markers with freshly isolated 2-NBDG (or Hoechst) Hi and Lo TIL subsets, as this approach could inform about their distinct functional profiles.

Point-by-point reply description of the revisions done:

We were perhaps overenthusiastic when we proposed to grow TILs in culture in large numbers for adoptive transfer assays. We have been able to stimulate FACS-sorted effector TILs in experiments ranging from 4 to 48 hours (new Fig. 3, A-D, new Fig. 6, and new Fig. 7), but could not expand them beyond 48 h, as cultures after this point suffered a substantial loss of viability (please see also our reply to Reviewer #2 in pages 21 and 23). We think that this could be workable with time and sufficient optimization, but we have considerably exceeded the time permitted for this revision, and we think it appropriate to submit our current new results to the

reviewers, as they provide some answers to this question (see next paragraph). We have also discussed this limitation in the discussion of our revised manuscript.

Our short-term assays *ex vivo* (4h to 24h and 48h) suggest that TILs in better or poorly perfused tumor regions have comparable intrinsic effector capability (Fig. 3, C and D), but their actual activity *in vivo* could differ because they express different levels of immunomodulatory receptors (PD-1, CTLA4, TIM3, LAG3) (Fig. 3, B and C) to interact with other cells in their vicinity, and might be exposed to different microenvironments, for instance higher expression of IFN-I by myeloid cells in low accessibility regions.

- Are the claims and the conclusions supported by the data or do they require additional experiments or analyses to support them?

The conclusions are supported, despite the needed experiments and some flaws in the controls. However, further investigation would be beneficial.

- Please request additional experiments only if they are essential for the conclusions. Alternatively, ask the authors to qualify their claims as preliminary or speculative, or to remove them altogether.

See above

- Are the suggested experiments realistic in terms of time and resources? It would help if you could add an estimated time investment for substantial experiments.

The suggested experiments are easy to perform, and maybe the authors already have some of them. I would say that three months would be enough.

- Are the data and the methods presented in such a way that they can be reproduced?

Yes, they are well described and can be reproduced.

- Are the experiments adequately replicated and statistical analysis adequate?

Yes

Reviewer #1 (Significance (Required)):

Provide contextual information to readers (editors and researchers) about the novelty of the study, its value for the field and the communities that might be interested.

The study is novel and studies an important subject: the conditions encountered by TILS, poor glucose access, and, therefore, metabolic pressure. The subject is important, and the approach

is adequate. There are some flaws in the experimental design, mostly in the in vitro/ex vivo experiments, but they can be resolved by the authors.

We thank the Reviewer for their positive assessment of our manuscript and for their thoughtful ideas to better strengthen our original findings.

The following aspects are important:

- Audience: basic and translational research in cancer but also applicable to other diseases such as autoimmune disease where T cell metabolism is pivotal.

- Please define your field of expertise with a few keywords to help the authors contextualize your point of view. Indicate if there are any parts of the paper that you do not have sufficient expertise to evaluate.

Immunology of T cells, cancer, immunotherapy

Reviewer #2 (Evidence, reproducibility and clarity (Required)):

Summary
In this manuscript, Riera-Borrull et al. first analyzed how glucose restriction might affect T lymphocyte function in vitro and found that T cells were capable of expressing certain cytokines such as IFNy and IL17A after stimulation, even with limited glucose availability. They then conducted in vivo experiments to assess expression profiles in tumor infiltrating T cells (TILs) in vivo with differential uptake of the glucose analog (i.e. 2-NBDG) and their accessibility to blood. Their findings revealed that 2-NBDGlo cells (i.e., presumably cells with less glucose uptake) exhibited a pronounced type I interferon signature compared to the 2-NBDGhi cells. Additionally, the uptake of 2-NBDG correlated with accessibility of TILs to blood vessels. The authors concluded that TILs with reduced access to blood upregulate ACSS2 to produce cellular acetyl-CoA, thereby sustaining gene expression, including ISGs. Lastly, the author observed that limited access to blood increased CXCR3 expression but decreased PD-1 expression in effector CD4 and CD8 TILs.

Major comments:

Overall, the story is highly descriptive. For example, cells consuming more glucose exhibit the same cellular ATP levels as the cells with limited glucose (Figures S1B and C). Why is this the case? Do these cells have the same mitochondrial respiration, TCA cycle activity and etc? Additionally, although glucose restriction exerts similar effects on ATP levels in Th1 and Th17 cells ex vivo, the cells display different cytokine expression changes in response to varying glucose concentrations, suggesting that glucose-derived ATP may not be the sole factor impacting cytokine expression in those cells. Therefore, additional experiments such as seahorse and 13C-labeled glucose tracing are required to further characterize the role of glucose in mediating cytokine expression in those cells. Moreover, the cytokine expression in stimulated T cells can be dynamic. Thus, a few more time points would be helpful to more precisely assess how glucose availability affects cytokine expression.

***Initial revision plan**:*

We appreciate the Reviewer's summary of our manuscript and the interesting points raised. We agree that our story is highly descriptive, but we think that the novelty of the findings we describe, as well as that they were not obviously predictable nor expected from the current literature makes our results highly original. Regarding their comments, some we can discuss here, whereas others we plan to address experimentally.

Our experiments show that T cells activated in culture in low glucose still use glucose as a main nutrient for ATP production, but are also more dependent on mitochondrial respiration (sensitivity to oligomycin and sodium azide) than cells in higher glucose (Figures 1F, 2B and S1C) (now renamed as Fig. 1, H and J, and Fig. EV1D). In view of these results, we think that Seahorse experiments would provide similar conclusions, that cells in low glucose would be more

dependent on respiration to sustain ATP. In this regard, early experiments by the Thompson lab showed that T cells activated with anti-CD3 plus anti-CD28 antibodies in 0.4 mM versus 11 mM glucose increased oxygen consumption, which the authors discussed as being necessary for maximal ATP generation under limited glucose supply (Frauwith K et al., (2002) Immunity 16:769). Also, Argüello and colleagues used SCENITH analysis, which quantifies protein translation in the presence of metabolic inhibitors as the equivalent to ATP availability, and found that it recapitulates Seahorse metabolic profiling, also showing that CD8 and CD4 T cell naïve versus effector memory subsets were all substantially and comparably dependent on glucose as a main energy source, but varied widely in their dependence on mitochondrial respiration (Argüello R et al., (2020) 32:1063, and Lopes N et al., (2021) Nat Immunol 22:179). Here we have used the same conceptual idea and tested the dependence of ATP on specific pathways by using metabolic inhibitors, as it is done with the SCENITH or Seahorse methods, but measuring intracellular ATP directly.

As to the question of how cells in low glucose can maintain similar ATP levels as those in higher glucose, our interpretation is that this can be so because they spend less ATP in low glucose conditions. For example, they do not proliferate (Figure 1D) (now Fig. 1E), and also have reduced mTORC1 activity (Figure S1D) (now Fig. 2A). Both effects would be expected to reduce overall translation and transcription rates, in turn reducing ATP expenditure (Argüello R et al., (2020) 32:1063; also He S. et al, (2011) PLoS ONE 6:e20107).

We agree with the Reviewer that glucose-derived ATP may not be the sole factor impacting cytokine expression in our experiments, and that other glucose-derived metabolites could influence the expression of different genes to varying degrees. However, regarding 13C-glucose tracing, if we wanted to trace glucose to cytokine expression, we would probably need to identify quantitative changes in glucose-derived metabolites affecting transcription regulatory processes in cells with low glucose availability. One parameter to analyze would be the incorporation of 13C-glucose-derived acetyl-CoA or glycosylation intermediates in histones and transcription factors, since T cell gene expression can be regulated by histone acetylation (Peng M et al., (2016) Science 354:481), and glycosylation and acetylation of transcription factors, for instance NF-kappaB and FOXP3 (Chen LF et al (2002) EMBO J 21:6539; Ramakrishnan P et al., (2014) Science Signaling 6:ra75; Loosdregt J et al., (2010) Blood 115:965; Gao C et al., (2023) BBA-Gene Regulatory Mechanisms 1866:194992). However, the problem here is to know how the label is distributed in promoters of glucose-sensitive genes, as it would be necessary to separate a precise chromatin region of interest from the rest of the cell chromatin and analyze glucose-derived posttranslational modifications in histones and transcription factors there. Techniques of reverse chromatin immunoprecipitation coupled with high-resolution mass spectrometry has been described to identify protein complexes at specific chromatin regions (MacKenzie T et al., (2023) Cells 12:1860). However, this is a very difficult methodology when non-repetitive regions such as promoters or enhancers are studied, and we have neither the expertise nor the instrumentation for approaching it and identifying the 13C labels in the relevant proteins. We respectfully think that these experiments, while interesting, would be out of our reach and perhaps would not

contribute substantially to other points of the manuscript raised by the Reviewer, which we think are more relevant to address.

We appreciate the Reviewer's suggestion that more time points could give us more precise information about cytokine expression, which will complement our current results. Nonetheless, our ELISA experiments in Figure 1C, measuring the cytokines accumulated in T cell culture supernatants 24 and 48 hours after activation, already suggest that at least IFNγ and IL-17 seem to plateau by 48 hours under either low or normal glucose.

Point-by-point reply description of the revisions done:

We again thank the Reviewer and acknowledge that the points raised are quite relevant and present major open questions about how T lymphocytes respond to altered nutrient microenvironments. As proposed in our *Revision Plan*, we have been able to address experimentally a basic metabolic analysis, and assess the dependence on glucose of TILs biosynthetic capacity in high and low blood accessibility tumor regions; their ability to produce cytokines freshly out of the tumor, and in normal and low glucose culture *ex vivo*; and analyzed their activation *ex vivo* at different time points (4h, 24h, 48h). These new results are detailed below.

i) We have used the SCENITH assay (manuscript Refs. Argüello R et al, 2020, and Lopes et al, 2021) to assess the dependence of effector CD4 and CD8 TILs on glycolysis and mitochondrial respiration, with respect to their better or poorer accessibility to blood. In this assay, the metabolic proficiency of the cells and their dependence on different ATP sources (glycolysis, mitochondrial respiration) are assessed by their rate of puromycin incorporation, a measure of protein translation and biosynthetic capacity, which can be quantified by flow cytometry. As shown in the original article describing this methodology (manuscript Ref. Argüello R et al, 2020), the incorporation of puromycin by T lymphocytes and diverse immune cell types is proportional to their ATP content, and so the assay is also informative about the energy balance of the cells.

We analyzed effector CD4 and CD8 TILs identifiable by their blood accessibility *in vivo* immediately after isolating them from the tumor (less than 1 hour), and found that the more blood-accessible TILs (Hoechst ^{Hi}) were also more metabolically active and likely had greater ATP production capacity than less accessible cells, as shown by their significantly higher protein translation rate (new Fig. 7A). We also observed that TILs in both high and low blood accessibility compartments were largely dependent on glucose rather than respiration for ATP production (new Fig. 7B). Therefore, our new results support the conclusion that effector TIL populations use glucose, even when present at very low concentrations, as a main fuel for ATP production. They also suggest that the reduced protein translation activity of TILs in tumor regions with poor blood accessibility could be due to their having less ATP production capacity, which in turn would lead to an attenuation of energy-expensive processes, like protein translation, to save ATP.

ii) We have also analyzed the production of IFNγ protein in CD4 and CD8 effector TILs in both high and low accessibility compartments upon a short (4 hours) stimulation *ex vivo* with PMA plus ionomycin (new Fig. 3D). Intriguingly, we observed a mildly higher expression of IFNγ protein in lower accessibility TILs, and this difference was detectable also in unstimulated cells, which suggests, as we discuss in the revised manuscript, that they might have been producing IFNγ *in vivo* while in the tumors. Although we are not sure of why this difference, it is possible that cells isolated from poorly perfused tumor regions respond more strongly when placed in a richer medium. Our result suggests that TILs in low-accessibility tumor regions are as intrinsically capable of rapid response to stimulation as those with better accessibility to blood. In the same experiment we also analyzed the expression of granzyme B and the capacity to release lytic granules (detected by the increase surface staining of CD107a) in effector CD8 TILs, and found comparable responses in cells with low and high accessibility. These new results suggest that TILs in better or poorly perfused tumor regions have comparable intrinsic effector capability, at least in the short term. However, as shown by our longer-term (48 hours) stimulation assays in new Fig. 3A, prolonged glucose restriction would be expected to decrease their cytokine output (please see results in iii) below). Besides their cytokine production profile, the activity of low accessibility TILs *in vivo* could be influenced by their lower expression of immunomodulatory receptors (PD-1, CTLA4, TIM3, LAG3) to interact with other cells in their vicinity (please see our reply to Reviewer #1 about the expression of immune checkpoint and exhaustion-exhaustion markers by TILs), and their exposure to different microenvironments, for instance IFN-I produced by myeloid cells also in low accessibility regions, which we describe below in the point about IFN-I sources.

iii) We have addressed the glucose dependence for cytokine expression of tumor-infiltrating T cells (TILs) isolated from LLC tumors and FACS-sorted as effector memory ($CD62L^{neg}$ $CD44^{+}$) CD4 T cells, excluding naïve and central memory T cells. Effector memory (abbreviated as effector) represent the large majority of TILs. We stimulated them *ex vivo* with anti-CD3 and anti-CD28 plus IL-2 in normal (5 mM) or low-glucose (0.3 mM) medium, and found that, similarly to other CD4 T cell sources used above in Fig. 1 and Fig. EV1, effector TILs could induce IL-17A and IFNγ mRNA when stimulated in 0.3 mM glucose (new Fig. 3A). However, this experiment also showed that TILs stimulated in low glucose had more mRNA and a higher mRNA-to-protein ratio than cells in 5 mM glucose, but produced less protein than those in 5 mM glucose over 48 hours (new Fig. 3A). TILs also produced IL-2 and TNFα better in 5 mM than 0.3 mM glucose (new Fig. EV2F). Still, TILs in low-glucose medium could produce substantial amounts of cytokine proteins, similarly to what we had seen with lymph node T cells in new Fig. 1 and Fig. EV1.

The authors identified enhanced type I interferon signaling in 2-NBDGlo cells but did not investigate this further. Questions remain, such as the source of IFN (tumors, T cells or other cells) and why 2-NBDGlo cells more responsive to IFN. The lack of explanations or mechanistic studies renders these results less compelling.

Initial revision plan:

We show that enhanced expression of type I interferon-response signature genes is a distinctive feature of 2-NBDG ^{Lo} and Hoechst ^{Lo} cells, and assessed the degree of dependence of these genes on the IFN alpha receptor (IFNAR) in experiments with IFNAR1 knockout mice. In other contexts, it has been found that bystander T cells that are not actually responding to antigens can be activated by local IFN-I, for instance in bystander-activated resident memory T cells in the lung (manuscript Ref. Low et al, 2020), or in CD8 infiltrating MC38 tumors (Lee KJ., (2024) Cell Reports Med 5:101567); and consequently display a characteristic signature of IFN-stimulated genes (ISGs).

We also coincide with the Reviewer in the importance of knowing the sources of IFN-I in the tumor. In order to identify possible sources of IFN-I in the tumor, in our revision plan we will isolate different immune cell types, as well as tumor and stromal cells to analyze their expression of type I IFNs. As to why 2-NBDG ^{Lo} T cells respond better, the experiments above could explain it if we find that T cells or other cells in low accessibility regions express higher levels of IFNs. In addition, we will test whether 2-NBDG ^{Lo} TILs express more IFNAR than 2-NBDG ^{Hi} ones, and will analyze their intracellular levels of phospho-STAT1 as a potential indicator of stronger IFNAR signaling. We have already seen that 2-NBDG ^{Lo} TILs have higher expression of STAT1 mRNA (an IFN-I-induced gene) (Figures 4C, 4D and 6C) (now Fig. 4, C and D, and Appendix Fig. S1, A and B), which makes it possible that they could accumulate more STAT1 protein to transduce IFNAR signals.

Point-by-point reply description of the revisions done:

We have now identified possible sources of IFN-I in the tumor microenvironment. First, TILs themselves were very poor IFN-I producers, as we had seen already in the RNA-seq analysis (deposited in GEO with accession number GSE250248). In this regard, here we show a table with the reads obtained for the respective mRNAs of several IFNα-encoding genes, whose expression is nearly undetectable. In the same analysis no reads were obtained for IFNβ. The table includes the IFN-induced chemokine CXCL10 and the cytokine IFNγ as a reference of two genes clearly detectable. Besides the RNA-seq, we could not detect IFNα and IFNβ1 mRNA in additional samples, the same ones in which we had detected IFN-I response genes (Fig. 4). We have not included this negative result as a figure because the RT-qPCR amplification of IFNα and IFNβ1 showed an “invalid” reading.

					RNA-seq reads			
chromosome	start position	end position	description	gene name	CD4 2NBDG Lo cDNA	CD4 2NBDG Hi cDNA	CD8 2NBDG Lo cDNA	CD8 2NBDG Hi cDNA
5	92346638	92348889	chemokine (C-X-C motif) ligand 10 [Source:MGI Symbol;Acc:MGI:1352450]	*Cxcl10*	11845	2801	3208	1368
10	118441046	118445892	interferon gamma [Source:MGI Symbol;Acc:MGI:107656]	*Ifng*	2778	5169	4060	3145
4	88841861	88842421	interferon alpha 4 [Source:MGI Symbol;Acc:MGI:107664]	*Ifna4*	1	0	0	2
4	88602580	88603376	interferon alpha 12 [Source:MGI Symbol;Acc:MGI:2676324]	*Ifna12*	0	0	2	0
4	88682881	88683912	interferon alpha 2 [Source:MGI Symbol;Acc:MGI:107666]	*Ifna2*	1	0	1	1
4	88675915	88676924	interferon alpha 16 [Source:MGI Symbol;Acc:MGI:3649260]	*Ifna16*	0	0	0	9
4	88591813	88592385	interferon alpha 9 [Source:MGI Symbol;Acc:MGI:107659]	*Ifna9*	0	0	0	2

In view of these results, we hypothesized that IFN-I could be produced by non-T cell sources, and sorted cell suspensions from tumors labeled *in vivo* with 2-NBDG into 2-NBDG ^{Lo} and ^{Hi} CD45-negative cells, which would comprise tumor cells and stroma, myeloid cells (pooled as CD11b $^{+}$ cells), NK1.1 $^{+}$ cells (NK and NKT), and B220 $^{+}$ B cells (new Fig. 5A). We found that IFNα and IFNβ1 were much more expressed in myeloid cells than in the other populations, and their expression was significantly higher in the 2-NBDG ^{Lo} fraction (new Fig. 5B). Likewise, 2-NBDG ^{Lo} myeloid cells also expressed the highest levels of the IFN-I-induced gene products IFIT1, CXCL10 and CXCL9. B cells and NK/NKT cells expressed low levels of IFNα but not IFNβ1; and NK/NKT had the highest expression of IFNγ, something expected since NK, NKT and T cells are main IFNγ producers (new Fig. 5B). These results showed that tumor regions with poor uptake of 2-NBDG harbored populations of IFN-I-producing myeloid cells, and suggested that their expression of IFNβ1 and IFNα led to the enhanced IFN-I response we had identified in 2-NBDG ^{Lo} TILs. In addition, we analyzed the expression of the IFN-I receptor (IFNAR1) in effector TILs and found a mildly higher surface expression in TILs that had lower accessibility to blood (Hoechst ^{Lo}) (new Fig. 5C). Altogether, these results support a scenario in which TILs in poorly accessible tumor regions present an IFN-I response signature, likely induced by myeloid cells in their microenvironment expressing IFNα and IFNβ1. We believe that these findings advance our current understanding of the effects of IFN-I in the tumor microenvironment.

The authors stated "2-NBDG uptake is thought to correlate with the metabolic activity of T cells (37), and our results below indicate that TILs with poor 2-NBDG uptake resemble T cells under glucose limitation" (Page 10, lines 200-201). However, the author should validate that 2-NBDGlo cells indeed have lower glucose concentration compared to the 2-NBDGhi cells. Otherwise, conclusions based on this assumption are not convincing. For instance, ISGs were reduced in T cells in low glucose medium but augmented in 2-NBDGlo cells, contradicting the statement that "our results below indicate that TILs with poor 2-NBDG uptake resemble T cells under glucose

limitation"!

Initial revision plan:

We thank the Reviewer for pointing this out, as it needs to be rewritten in a clearer way to avoid confusing statements. In view of our results, we would conclude that TILs with poor 2-NBDG uptake exhibit some features found in T cells under glucose limitation (as we wrote at the end of that section of results), but also exhibit higher expression of diverse glucose-responsive ISGs. One possible explanation, which we plan to address experimentally, could be that there was more IFN-I in the local microenvironment of 2-NBDG ^{Lo} cells and even if they had low glucose, they would still induce higher expression of ISGs than cells in a more blood-accessible region. This is consistent with our results in Supplementary Figure S2 (now Fig. EV4) showing that adding IFN-I to T cells activated in low-glucose induces a higher expression of ISGs (CXCL10, IFIT1, STAT1) than stimulating cells in normal glucose but without extra IFN-I. We should also include in the paper the discussion that T cells displaying signs of being under limited glucose availability does not mean that they are starved of glucose and therefore they could maintain some glucose-dependent functions while downregulating others. This notion connects with recent results of the Rathmell lab (manuscript Ref. Reinfeld et al, 2021) showing that on average TILs in subcutaneous MC38 tumors take as much glucose per cell as the bulk of the whole tumor, and even more than T cells in the spleen do, which suggests that TILs could have enough glucose to maintain significant functionality. However, this work did not separate TILs in high versus low glucose uptake, and it is likely that our 2-NBDG ^{Lo} / Hoechst ^{Lo} cells might represent the subsets with the lowest, but not entirely lacking, nutrient availability in a generally glucose-sufficient microenvironment. Besides rewriting these sentences, we hope that the experiments we will do to address the previous and next points would provide additional answers.

Point-by-point reply description of the revisions done:

We agree with the reviewer that the previous writing could be confusing. Our results (former Supplementary Figure S2, now Fig. EV4) showed that IFN-I stimulation can synergize with T cell receptor signaling to induce glucose-dependent IFN-I-stimulated genes (ISGs), even in low glucose conditions, so that adding IFN-I to T cells activated in 0.3 mM glucose induced higher expression of ISGs than stimulating cells in 15 mM glucose but without extra IFN-I (now Fig. EV4). In this situation, it would not be contradictory that cells in low glucose expressed ISGs better than cells in normal glucose, if they had extra IFN-I stimulation. Nonetheless, whereas cells in low glucose can maintain transcription of several glucose-dependent genes, they also exhibit reduced overall protein translation and proliferation, both glucose-dependent functions. We have rewritten the sections of results describing the potential similarities of TILs with reduced uptake of 2-NBDG and Hoechst probes to cells under glucose limitation. We have also included in the paper the discussion that T cells displaying signs of being under limited glucose availability does not mean that they are starved of glucose and therefore they could maintain some glucose-dependent functions while downregulating others.

Regarding the higher expression of ISGs in 2-NBDG ^{Lo} TILs, an additional point is our finding that myeloid cells in poorly accessible tumor regions express more IFN-I than in highly accessible regions and also more than any other immune or non-immune cell type, which could explain the higher expression of ISGs in TILs in poorly perfused regions (new Fig. 5B).

Some conclusions lack solid supporting evidence. Although the authors cited previous studies, certain experiments are necessary to substantiate their conclusions. For example, the authors concluded that 2-NBDGlo cells upregulate ACSS2 to produce acetyl-CoA for gene expression without actually measuring acetyl-CoA in those cells. We do not know whether: 1) 2-NBDGlo cells have low glucose; 2) 2-NBDGlo cells have low acetyl-CoA (Acetyl-CoA can be generated through other routes, such as fatty acid oxidation); 3) Increased ACSS2 necessarily leads to more acetyl-CoA production in those cells.

ACSSi experiments are also unconvincing, as the authors do not provide any data to demonstrate that the inhibitors function properly and that the downstream effects are not due to off-target effects. Moreover, it is possible that TILs with different accessibility to blood take up different amounts of the ACSSi, which could affect the comparison of cytokine expression in Hoechst hi/lo cells.

***Initial revision plan**:*

These are valid points and indeed we thought about them while working on this manuscript. One of our main concerns here when trying to measure intracellular acetyl-CoA or glucose in Hoechst or 2-NBDG ^{Hi} versus ^{Lo} (Hi vs Lo) effector CD4 or CD8 cells is that the sorting of these subsets yields very few cells (in the range of a few hundreds) from each tumor, and so we might be unable to detect these molecules. In case we could not detect acetyl-CoA or glucose in so few cells after sorting, we can ask about acetyl-CoA and glucose levels in TILs expanded ex vivo, which we hope would allow us to obtain more cells, and then cultured in nutrient-rich or nutrient-poor medium, without or with acetate supplementation. If these experiments worked, we could use them as well to test the effect of ACSS2 inhibitors on the intracellular levels of acetyl-CoA in TILs grown in glucose-sufficient or restricted conditions.

As to the possibility that TILs with different accessibility to blood take up different amounts of ACSS2i, we can discuss that our results show that whereas IFNγ is more inhibited by ACSS2i in more accessible cells, the opposite is seen with IFIT1, whose expression is more repressed by ACSS2i in less accessible cells (Figure 6C) (now Fig. EV5). In the same experiments, expression of CXCL10, CTLA4 and FOXP3 mRNA in CD4 effector TILs was comparably inhibited by ACSS2i in higher and lower accessibility cells. Our interpretation of these results is that ACSS2i, after an administration regimen of 5 consecutive daily injections, could inhibit the expression of sensitive genes in both high and low accessibility regions. Nonetheless, the sensitivity of specific genes to ACSS2 inhibitors could be tested in TILs expanded in culture to ensure homogenous access to the inhibitors. With regard to the off-target effects of ACSS2i, we plan to use two chemically independent drugs, ACSS2i (VY-3-249), and the more recently developed and more potent VY-

3-135, which we expect will reduce the likelihood that convergent results are due to off-target ACSS2i effects.

Point-by-point reply description of the revisions done:

We had hoped to expand in culture FACS-sorted effector TILs isolated by their higher or lower blood accessibility, in order to obtain sufficient numbers of cells for metabolite detection. Whereas we have successfully carried out experiments stimulating FACS-sorted TILs ranging from 4 to 48 hours (new Fig. 3, A-D, new Fig. 6, and new Fig. 7), we could not expand them beyond 48 h, as cultures after this point suffered a substantial loss of viability. In the revised manuscript we have discussed that a limitation of our work is that we could not determine intracellular levels of glucose and acetate, and the fate of their metabolites in *in vivo* effector TILs in tumor regions with poor or high blood accessibility.

As to the role of ACSS2 in gene expression in TILs with different accessibility to blood *in vivo*, we have now tested a chemically independent, recently developed ACSS2 inhibitor (VY-3-135) (manuscript Ref. Miller et al, 2023). We observed that 3 doses of VY-3-135 starting at day 10 after tumor inoculation caused a significantly inhibition of LLC tumor growth soon after having begun the treatment (new Fig. EV5G). This antitumor effectiveness was in agreement with published work on other mouse tumor models (manuscript Ref. Miller et al, 2023). But then, we found that VY-3-135 affected IFN-I response genes differently from ACSS2i/VY-3-249 in our original experiment, and whereas ACSS2i/VY-3-249 had attenuated the expression of CXCL10 and IFIT1 mRNA in TILs, VY-3-135 did not inhibit them (new Fig. EV5, E and H). Miller et al., had shown that a 7-day regime of VY-3-135, longer than our 3 doses, enhanced the expression of several IFN-I response genes including *Ifit1* and *Cxcl10*, in TILs from Brpkp110 mouse mammary tumors, and also showed that a much longer treatment (28 days) enhanced the protein expression of IFNγ (CD4) and granzyme B (CD8) in TILs (manuscript Ref. Miller et al., 2023). We did not see that VY-3-135 increased the levels of IFNγ mRNA in TILs, although it did not cause either the mild inhibition of their expression that we had seen with ACSS2i/VY-3-249 (new Fig. EV5, E and H). Finally, Miller et al, also reported that ACSS2-deficient immune cells were as competent as wild-type ones in antitumor capacity, and concluded that the overall antitumor effect of the VY-3-135 was attributable to the inhibition of ACSS2 in tumor cells, which freed available acetate to be used by TILs.

The results by Miller et al., differed from a previous work (manuscript Ref. Qiu et al, 2019) who showed that reducing ACSS2 expression by shRNA knockdown in OT-I TCR transgenic CD8 T cells made them poorer IFNγ producers and weaker tumor controllers when infiltrating EL4-OVA subcutaneous tumors *in vivo*. As we discuss in the revised manuscript in connection with our results, the differing results between Miller et al. and Qiu et al. about the role of ACSS2 in the antitumor activity of TILs could be due to differences in the degree of inhibition of ACSS2 in their different experimental systems, and the balance of acetate (the substrate for ACSS2) versus other metabolites in the TIL microenvironment. In this regard, work by the Hess laboratory showed that acetate can downregulate the expression of ACSS2 itself, and have pro- and anti-inflammatory

effects (such as enhancing or inhibiting the expression of IFNγ) depending on the activation stage of the T cell (manuscript Ref. Balmer et al, 2020).

Seeing that these previous works reported different results as to the possible dependence of TILs on ACSS2, and that the ACSS2 inhibitors in our experiments *in vivo* had different, and relatively modest, effects on gene expression in TILs with better or poorer blood accessibility, we tested them directly in effector CD4 TILs activated *ex vivo* with anti-CD3 and anti-CD28 plus IFNα and IFNβ1. Both inhibitors were similarly effective at reducing the expression of IFNγ and CXCL10 mRNA in CD4 effector TILs, but only ACSS2i/VY-3-249 inhibited the induction of IFIT1(new Fig. 6A). Induction of STAT1 was only mildly inhibited by ACSS2i/VY-3-249 and not by VY-3-135 (new Fig. 6A). Analysis of cytokine proteins showed that both ACSS2 inhibitors reduced the production of IFNγ and IL-17A, but only ACSS2i/VY-3-249 was moderately inhibitory for TNFα and IL-2 (new Fig. 6B). Therefore, our results indicated that ACSS2 can contribute to the expression of cytokines and several interferon-I responsive genes in effector TILs activated via T cell receptor and type I IFN stimulation. However, of the two inhibitors only ACSS2i/VY-3-249 showed a similar effect *in vivo* and *ex vivo*, whereas VY-3-135 did not inhibit TILs, and yet it significantly reduced tumor growth. We have discussed in the revised manuscript that differences between both ACSS2 inhibitors *in vivo* might involve indirect effects from other cells in the tumor microenvironment that could be variably sensitive to the inhibitors, or differences in the pharmacokinetics and metabolizing of these drugs. We consider that the results from our new experiments with TILs *in vivo* and *ex vivo* provide useful information toward understanding the role of ACSS2 in T cells in the tumor microenvironment.

Reviewer #2 (Significance (Required)):

Significance
The major issue of this study is the lack of mechanistic insights, which significantly diminishes both impact and novelty of the findings. Much of the data is descriptive. The findings are not particularly exciting, also partially due to the absence of mechanisms. With more mechanistic insights and solid characterizations, the study could be of interest in the fields of immunometabolism and tumor immunotherapy.

Initial revision plan:

We agree that carrying out the diverse experiments proposed by the reviewers will yield new mechanistic insights to improve our work. However, we would like to point out the novelty of our manuscript in uncovering the remarkable efficiency of activated T cells to maintain glucose-dependent functions under very low glucose levels, and identifying markers of tumor-infiltrating, effector CD4 and CD8 T cells that differ between cells with higher and lower accessibility to blood, such as the expression of IFN-I response signatures, the chemokine receptor CXCR3, and PD-1. Previous works analyzing the function and metabolic characteristics of tumor-infiltrating T cells had not identified specific features of effector TIL subsets distinguishable by their accessibility to

blood. Our findings here offer an alternative scenario to the commonly held view that T cells in poorly perfused tumor regions are largely inactive due to nutrient insufficiency.

Point-by-point reply description of the revisions done:

We agree with the Reviewer about the importance of determining the actual content of glucose and metabolites like acetate, which might modulate glucose-dependent gene expression. Also in this direction, it would be useful to track the fate of their intracellular metabolites *in vivo* in effector TILs in tumor regions with poor or high blood accessibility. As we had discussed in our *Revision Plan*, detecting intracellular glucose and acetate, or their derived metabolites such as Ac-CoA, in the small number of sorted Hoechst ^{Hi} and ^{Lo} TILs is beyond our capabilities, as we lack the resources and methodological expertise for it. This is why we had initially proposed to expand TILs, which we would have used to culture them in larger numbers in nutrient-rich or nutrient-poor medium, without or with acetate supplementation, and then analyzed intracellular metabolites. One potential problem with this approach, though, is that TILs expanded *ex vivo* might adapt to the new culture conditions and lose part of the original characteristics they had in the tumor. Unfortunately, as explained above and to Reviewer #1, we have not been able to expand TILs. We knew from previous observations in our group that T cells loaded with the 2-deoxyglucose analog 2-NBDG and the DNA intercalating agent Hoechst 33342 do not proliferate well, and in order to avoid the possible toxicity of these compounds, we tried expanding only unlabeled cells. But although we could activate them for up to 48 hours (as in Fig. 3A and Fig. EV2F), we could not get them to proliferate. Despite these obstacles, we have been able to carry out a metabolic profiling of TILs fresh out of the tumor using the SCENITH assay, and assessed the dependence on glucose and mitochondrial respiration of TILs biosynthetic capacity in high and low blood accessibility tumor regions (new Fig. 7). We have also compared two independent ACSS2 inhibitors *in vivo* and with isolated TILs *ex vivo*, which has yielded some answers about to the potential role of ACSS2 in TILs (new Fig. 6 and Fig. EV6).

Finally, throughout the revision work we have been able to address experimentally and solve important questions about the functionality of TILs in poorly accessible tumor regions, and their responsiveness to the microenvironment. We have obtained new results that we believe enhance significantly the novelty and mechanistic insights of our manuscript. To highlight from our new findings:

i) Effector TILs in poorly accessible tumor regions have reduced metabolic activity but fundamentally depend on glucose for ATP production, as do TILs in highly blood-accessible regions.

ii) TILs with reduced access to blood had comparable cell-intrinsic capacity for inducing IFNγ and granzyme B to TILs with fuller accessibility to blood, however, as suggested by our longer-term stimulation experiments prolonged glucose restriction would decrease their cytokine output.

iii) Low-accessibility TILs exhibited a constitutively elevated signature of type I IFN (IFN-I) response genes, characteristic of bystander resident memory cells. Here we have identified myeloid cells in tumor regions with poor blood accessibility as the major source of IFN-I.

iv) Unexpectedly, effector TILs in poorly accessible tumor regions had reduced expression of immune checkpoint, exhaustion and Treg-associated proteins. Thus, effector T lymphocytes in poorly perfused tumor regions can maintain specific glucose-dependent responses, and might be partially protected from inhibitory and exhausting pressure from the tumor microenvironment.

My expertise includes metabolism and cell signaling. I find that the metabolic studies in this manuscript are disappointing and require further investigations.

Reviewer #3 (Evidence, reproducibility and clarity (Required)):

In the present study, Riera-Borrull et al aimed to characterize the functional and transcriptional signatures of tumor infiltrating CD4+ T cells, in the context of distinct location or differential access to blood-delivered nutrients, in particular the glucose (mainly based on 2-NBDG staining pattern). The impact of glucose on Th1 or Th17 differentiation has been well studied. I have the following major concerns:

We thank the reviewer for their positive assessment of our study and we acknowledge their suggestions below for strengthening our findings.

1. The cytokine production profile was mainly measured by real-time PCR assay, and the key factors should also be measured at the protein level, either with FACS or ELISA or CBA.

***Initial revision plan**:*

We thank the Reviewer for this comment, and in our revision plan we will analyze the expression of key proteins as suggested.

Point-by-point reply description of the revisions done:

This is a relevant point, especially after having found that TILs with reduced accessibility to blood have reduced translation activity. We have analyzed cytokine protein production and mRNA expression in naïve (CD62L $^{+}$ CD44 ^{neg}) CD4 T cells stimulated as Th17 cells (new Fig. 1A, and Fig. EV1A), and show that they express IL-17A mRNA comparably when activated in 0.3 or 5 mM glucose, but IL-17A protein was better induced in 5 mM (new Fig. 1A). By contrast to preactivated CD4 T cells, we did not detect IL-22 and IFNγ mRNA reliably in naïve ones after 48 hours of Th17 stimulation, nor significant accumulation of either cytokine in the culture supernatant (new Fig. 1A). In the same experiments we found that TNFα was better produced in 5 mM than 0.3 mM glucose, whereas IL-2 production was not decreased in low glucose (new Fig. EV1A).

We have addressed the glucose dependence for cytokine protein expression of tumor-infiltrating T cells (TILs) isolated from LLC tumors and FACS-sorted as effector memory (CD62L ^{neg} CD44 $^{+}$) CD4 T cells, excluding naïve and central memory T cells. We found that effector TILs stimulated in low glucose expressed more IL-17A and IFNγ mRNA and had a higher mRNA-to-protein ratio than cells in 5 mM glucose, but cells in 5 mM produced more protein over 48 hours (Fig. 3A). TILs also produced IL-2 and TNFα better in 5 mM than 0.3 mM glucose (new Fig. EV2E). Nonetheless, production of cytokine proteins by TILs in low-glucose medium was still substantial, similarly to what we had seen with lymph node T cells in new Fig. 1 and Fig. EV1.

We have also analyzed the production of IFNγ protein in CD4 and CD8 effector TILs in both high and low accessibility compartments upon a short (4 hours) stimulation *ex vivo* with PMA plus ionomycin (new Fig. 3D). Intriguingly, we observed a mildly higher expression of IFNγ in lower

accessibility TILs, and this difference was detectable also in unstimulated cells, which suggests that they might have been producing IFNγ *in vivo* while in the tumors. Although we are not sure of why this difference, it is possible that cells isolated from poorly perfused tumor regions respond more strongly when placed in a richer medium. This result suggests that TILs in low-accessibility tumor regions are as intrinsically capable of rapid response to stimulation as those with better accessibility to blood. Nonetheless, as suggested by our longer-term (48 hours) stimulation experiments in new Fig. 3A and new Fig. EV2F, prolonged glucose restriction would be expected to decrease their cytokine output.

2. The authors aimed to compare T cell characteristics with differential access to blood-delivered glucose, however, the authors did not provide any metabolic assay or parameters to prove their distinct metabolic activity.

Initial revision plan:

We agree with the Reviewer that this point is worth addressing, and we have given considerable thought to how we can solve this question with the very small number (few hundreds) of effector TILs in the 2-NBDG (or Hoechst) Hi and Lo subsets we get after sorting. We think that perhaps our best option here could be to try to analyze metabolic parameters, such as their dependence on glycolysis versus mitochondrial respiration for ATP production, using SCENITH or a similar assay based on puromycin incorporation and flow cytometry, as this technique is suitable for small numbers of cells. We have also considered isolating whole TILs (without sorting into high and low blood accessibility subsets) and stimulating them in culture in normal or nutrient-depleted medium, and then probing their sensitivity to different metabolic inhibitors for ATP production. We plan to test this approach, as it has the advantage of allowing us to expand the cultures and obtaining more cells. However, it also has the caveat that TILs cultured in a different medium than they were in vivo might not necessarily exhibit equivalent properties to TILs originally residing in higher and lower blood accessibility regions.

Point-by-point reply description of the revisions done:

We have used the SCENITH assay to address this relevant point and assess the dependence of effector CD4 and CD8 TILs on glycolysis and mitochondrial respiration, with respect to their better or poorer accessibility to blood. In this assay, the metabolic proficiency of the cells and their dependence on different ATP sources (glycolysis, mitochondrial respiration) are assessed by their rate of puromycin incorporation, a measure of protein translation and biosynthetic capacity (manuscript Ref. Argüello et al, 2020). We used explants of tumor labeled with Hoechst *in vivo* to measure the rate of puromycin incorporation in TILs identifiable as cells with higher or lower blood accessibility. We found that effector CD4 and CD8 TILs with greater blood accessibility *in vivo* were more metabolically active than less accessible cells, as shown by their significantly higher protein translation rate (new Fig. 7A). We also observed that TILs in both high and low blood accessibility compartments were largely dependent on glucose rather than mitochondrial respiration for ATP production (new Fig. 7B). Our new findings are in line with our previous ATP

production analyses with lymph node T cells, in showing that effector T cells, even in low glucose and nutrient-limited conditions, still use glucose as a fundamental source for ATP production. They also suggest that the reduced protein translation activity of TILs in tumor regions with poor blood accessibility could be due to their having less ATP production capacity, which in turn would lead to an attenuation of energy-expensive processes, like protein translation, to save ATP.

3. Beyond the effector function, the availability of glucose or their metabolites certainly shape the differentiation status of tumor infiltrating T cells. In this regard, how about the stemness or exhaustion status of those 2-NBDG low or high T cells?

Initial revision plan:

This is a very interesting point, as stemness and exhaustion of tumor-infiltrating T cells are relevant in tumor progression. We have analyzed the expression of PD-1 in Figure 7, where we show that 2-NBDG ^{Lo} and Hoechst ^{Lo} CD4 and CD8 T cells have lower surface expression of PD-1 than their 2-NBDG ^{Hi} and Hoechst ^{Hi} counterparts. Our RNA-seq data for exhaustion-associated markers TIM-3 (Havcr2) and LAG-3 show moderately higher expression of both in 2-NBDG ^{Lo} effector CD8 TILs than in 2-NBDG ^{Hi} ones, but fewer reads for them in 2-NBDG ^{Lo} than in 2-NBDG ^{Hi} effector CD4 TILs. Regarding the stemness factor TCF1 (encoded by Tcf7), our RNA-seq data show a very mild (~ 1.2-fold) increase in its expression in 2-NBDG ^{Lo} effector CD4 and CD8 TILs. Our revision plan will analyze mRNA and protein levels for these markers in 2-NBDG or Hoechst Hi/Lo CD4 and CD8 TILs.

Point-by-point reply description of the revisions done:

We have extended our previous finding that TILs with better access to intravenously injected Hoechst and 2-NBDG had higher expression of PD-1 (CD4 and CD8 TILs), and now we show that, besides expressing more PD-1, TILs with higher Hoechst uptake express more of the Treg markers FOXP3 and CTLA4 (CD4 TILs), and the exhaustion proteins LAG3 (CD4 and CD8 TILs), and TIM3 (CD8 TILs), shown as new Fig, 3, B and C. Regarding the stemness marker TCF1, we have analyzed its mRNA levels in 2-NBDG ^{Lo} and ^{Hi} CD4 and CD8 effector TILs and did not find any difference between cells with higher or lower 2-NBDG uptake. We are including this analysis here, (Reviewer Response Fig. R1), but we think that it would not be necessary to show it in the revised manuscript.

Review COMMONS

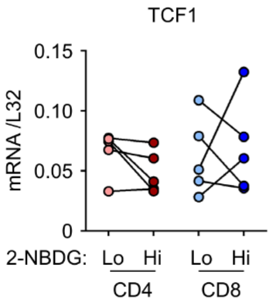

Reviewer Response Figure R1. mRNA expression of the stemness marker TCF1 in tumor-infiltrating CD4 and CD8 T lymphocytes with reduced uptake of 2-NBDG *in vivo*. Expression of TCF1 mRNA in effector CD4 and CD8 TILs sorted by their 2-NBDG uptake *in vivo*. Results are from one experiment. Dots are connected by lines to better visualize differences between 2-NBDG ^{Lo} vs ^{Hi} cells within each individual tumor.

Altogether, our new results show that TILs in low accessibility tumor regions express significantly lower levels of T regulatory and exhaustion-associated proteins, which suggests that they might be less vulnerable to suppressor mechanisms from the tumor microenvironment than cells in regions with better blood accessibility. We found this initially surprising, but then, while setting up the puromycin incorporation assay for SCENITH, we found an article that analyzed how mTOR activity and protein translation rate correlated with activation and exhaustion features in TILs, and whose conclusions are complementary to our new findings (manuscript Ref. De Ponte Conti et al, 2021). The authors identified TILs with higher or lower protein translation capacity by their ability to incorporate puromycin *in vivo*, and showed that CD4 TILs with higher biosynthetic capacity contained higher proportions of Treg cells by FOXP3 and CTLA4 protein expression, and that CD8 TILs with higher translation capacity also expressed more PD-1 and TIM3 surface proteins. These findings recall our own, which show that TILs in regions more accessible to blood, identified by their higher *in vivo* uptake of Hoechst 33342, are more metabolically active and have higher expression of Treg, checkpoint and exhaustion-associated proteins. That complementary results were obtained by De Ponte Conti and us through independent approaches and using different strategies and tumor models to analyze TILs, is quite relevant as it indicates that poorly perfused tumor regions could contain a previously unappreciated reserve of functionally competent effector T cells, in contrast with areas better irrigated by blood, where TILs might face stronger pressure from inhibitory (Treg, immune checkpoints) and exhaustion-inducing mechanisms.

4. What are the underlying mechanisms of differential CXCR3 expression pattern?

Initial revision plan:

Our current thinking about how CXCR3 is more expressed in 2-NBDG ^{Lo} and Hoechst ^{Lo} effector TILs is based on our findings and the literature. One possibility is that it could result from the enhanced responsiveness to IFNs in low-accessibility TILs, as we see that CXCR3 is partially dependent on IFNAR signaling (Figure 4). In addition, it has been described that IFNγ is a potent inducer of CXCR3 (Nakajima C et al., (2002) Eur. J. Immunol. 32:1792). On the other hand, CXCR3 expression can be repressed by the cytokine TFGβ and STAT3-activating signals (Gunderson AJ et al., (2020) Nature Comm. 11:1749; Yue C et al., (2015) Cancer Immunol. Res. 3:864; Sun Q et al., (2023) J. Exp. Med. 220:20220686).

In our revision we will discuss these possibilities, and in terms of experiments, we plan to isolate different immune cell types, as well as tumor cells with low or high blood accessibility to analyze their expression of cytokines (IFNs, TGFβ, IL-10 as a STAT3-activating cytokine) that could regulate CXCR3 expression in T cells.

Point-by-point reply description of the revisions done:

We have devoted a considerable effort to trying to get clear answers about CXCR3 expression in TILs in our assays. In our previous version of the manuscript, we analyzed the expression of CXCR3 in TILs from LLC tumors, since CXCR3 is the receptor for cytokines like CXCL10 whose mRNA was more expressed in low accessibility cells (new Figs. 4, 5; and Appendix Fig. S1, A and B). In our original experiment we had already noticed that the expression of CXCR3 (detected with an Alexa Fluor 700-labeled antibody, R&D Systems, catalog: FAB1685N) was quite low and close to background levels (see below Exp #1 in Reviewer Response Fig. R2A), and in later flow cytometry experiments we have again found very poor CXCR3 expression in TILs. We have tried a different antibody and fluorochrome combination (PE-labeled anti-mouse CXCR3, Miltenyi, catalog: 130-111-087), but again the staining was low (Exp #2 in Reviewer Response Fig. R2A). However, when we have tested the antibody in splenocytes, we did obtain a clear CXCR3 signal in spleen effector T cells, in contrast with the much weaker CXCR3 expression in TILs (Exp #2 in Reviewer Response Fig. R2A). This result confirmed that TILs in LLC tumors expressed very low to low levels of surface CXCR3 in comparison with other, non-TIL, T lymphocytes. Also, when we compared the expression of CXCR3 in Hoechst ^{Lo} versus ^{Hi} in this experiment, we now found moderately higher CXCR3 surface staining in Hoechst ^{Hi} TILs (Reviewer Response Fig. R2, A and B). However, in both the previous and recent experiments PD-1 surface expression was noticeably and consistently higher in Hoechst ^{Hi} TILs (Reviewer Response Fig. R2, A and B). Seeing that CXCR3 detection in LLC TILs is poor, and that we could not establish conclusive differences in its expression between Hoechst ^{Lo} and ^{Hi} effector TIL subsets, we are unsure about the functional relevance of these variations in highly or poorly blood-accessible TILs. We think that these results have limited value in the manuscript and have consolidated them in the figure below (Reviewer Response Fig. R2) instead of showing them in the manuscript. In the manuscript we have discussed that it remains to be determined whether TILs in less accessible tumor regions are poorer or better responders to CXCR3 ligands than those in well-perfused regions. Nonetheless, if the reviewers and Editor consider that it might be useful for readers to see them in the manuscript, we can add them to Appendix Figure S1.

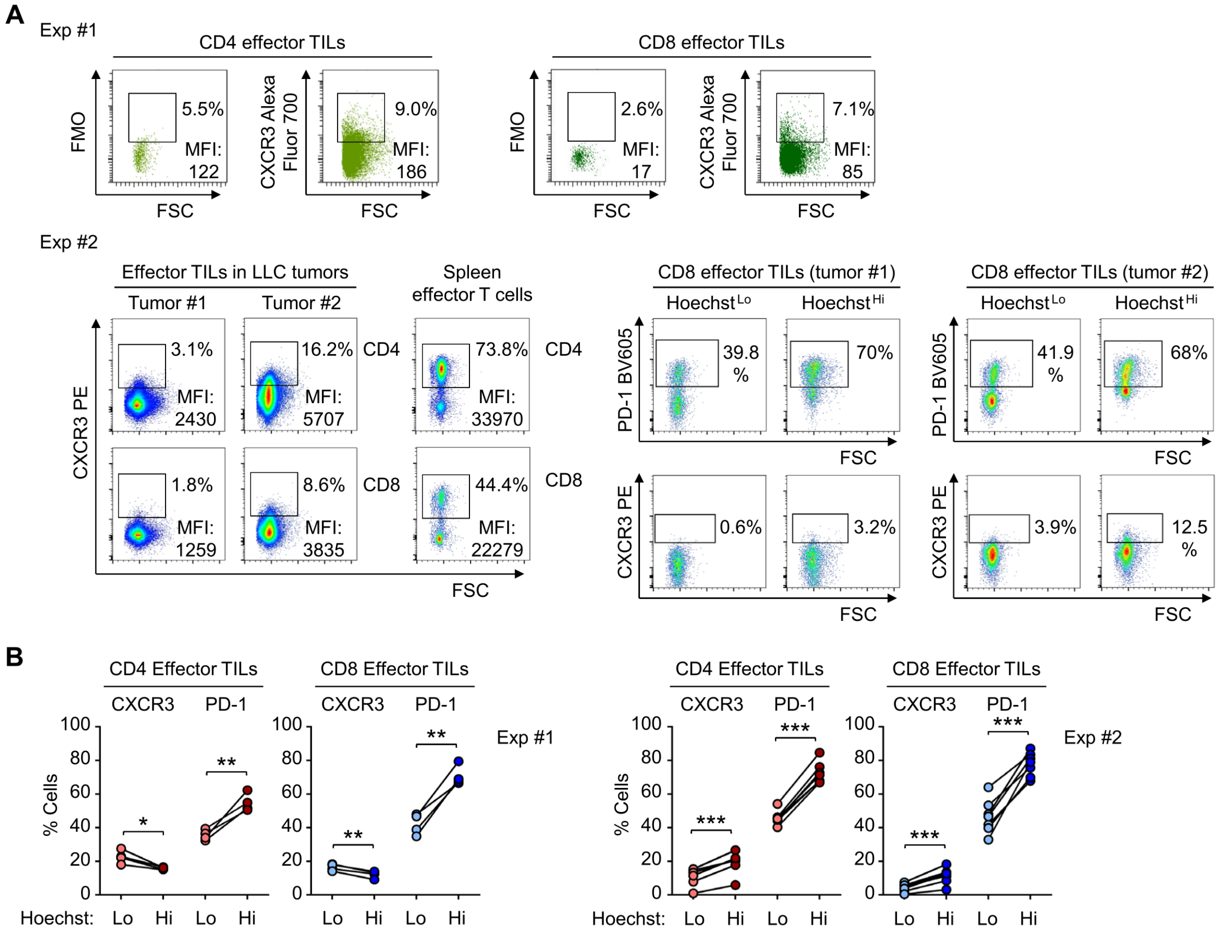

Reviewer Response Figure R2. Protein expression of CXCR3 and PD-1 in TILs of poorly and highly perfused tumor regions. (A) Plots illustrate the surface expression of CXCR3 in CD4 and CD8 effector TILs in one tumor from Exp #1, and in CD8 and CD4 effector T cells from spleen, and TILs from two tumors in Exp #2. **(B)** % cells expressing CXCR3 and PD-1 surface proteins in Hoechst ^{Lo} versus ^{Hi} cells CD4 and CD8 effector TILs from experiment #1 (4 mice) and experiment #2 (7 mice). Expression of CXCR3 and PD-1 was quantified in the 15% lowest or highest Hoechst-stained populations. Experiments #1 and #2 used the same anti-PD-1 antibody (Brilliant Violet 605, clone 29F.1A12), but different antibodies for CXCR3 (Alexa Fluor 700-labeled, clone 220803 in Exp #1, and PE-labeled, clone REA724 in Exp #2), and different cytometers. Statistical significance was assessed with a paired *t* test. * $P < 0.05$; ** $P < 0.01$; *** $P < 0.001$.

5. How about the CD8+ T cells?

Initial revision plan:

Our experiments in Figures 3 to 7 and in supplementary Figure S2 include a parallel analysis of CD4 and CD8 TILs. As for the new experiments we plan to address the reviewers' questions, they will also be done in CD4 as well as CD8 cells.

Point-by-point reply description of the revisions done:

In addition to experiments in the previous version that already included a parallel analysis of CD4 and CD8 TILs, our new experiments analyzing checkpoint and exhaustion markers, effect of the new ACSS2 inhibitor VY-3-135 *in vivo*, and metabolic activity and ATP dependence on glucose, in TILs include both CD4 and CD8 effector T cells (new Fig. 3, C, E, F; Fig. 4; Fig. 5C; Fig. 7; and new Fig. EV3, A-C; Appendix Fig. S1, A-D; Fig. EV4; and Fig. EV5, B, E, H)

Reviewer #3 (Significance (Required)):

The conceptual novelty is limited given a lot of studies in this relevant field.

Initial revision plan:

We would like to point out the relevant findings in our manuscript that support its novelty. First, our results uncover a remarkable efficiency of activated T cells to maintain glucose-dependent functions under very low glucose levels. Second, we identify markers of tumor-infiltrating, effector CD4 and CD8 T cells that differ between cells with higher and lower accessibility to blood, such as the expression of IFN-I response signatures, the chemokine receptor CXCR3, and PD-1. Thus, our results show that tumor-infiltrating effector T cells with reduced access to blood exhibit some characteristics of glucose-restricted cells, but also show characteristics of activated and potentially antitumor-capable cells. Previous works about tumor-infiltrating T cells had not identified specific features of effector TIL subsets distinguishable by their accessibility to blood, and our findings here offer an alternative scenario to the commonly held view that T cells in poorly perfused tumor regions are largely inactive due to nutrient insufficiency.

Point-by-point reply description of the revisions done:

An important point of novelty in our original manuscript was the uncovering of several unexcepted features of TILs in low accessibility tumor regions, and as result of the revision, we have now obtained significant new results that we think further enhance the novelty and mechanistic insights of our manuscript and contribute relevant findings to the study of TIL regulation by nutrients. We would like to highlight the following:

i) Effector TILs in poorly accessible tumor regions have reduced metabolic activity but fundamentally depend on glucose for ATP production, as do TILs in highly blood-accessible regions.

ii) TILs with reduced access to blood had comparable cell-intrinsic capacity for inducing IFNγ and granzyme B to TILs with fuller accessibility to blood, however, as suggested by our longer-term stimulation experiments prolonged glucose restriction would decrease their cytokine output.

iii) Low-accessibility TILs exhibited a constitutively elevated signature of type I IFN (IFN-I) response genes, characteristic of bystander resident memory cells. Here we have also identified myeloid cells in tumor regions with poor blood accessibility as the major source of IFN-I.

iv) Unexpectedly, effector TILs in poorly accessible tumor regions had reduced expression of immune checkpoint, exhaustion and Treg-associated proteins. Thus, effector T lymphocytes in poorly perfused tumor regions can maintain specific glucose-dependent responses, and might be partially protected from inhibitory and exhausting pressure from the tumor microenvironment.

Thus, our results show that tumor-infiltrating effector T cells with reduced access to blood exhibit some characteristics of nutrients-restricted cells, but also show characteristics of activated T cells with potentially enhanced antitumor capacity. Our new results offer an alternative scenario to the commonly held view that TILs in poorly perfused tumor regions are largely inactive due to nutrient insufficiency, and suggest that on the contrary, they have substantial cell-intrinsic functionality and might be partially protected from inhibitory and exhausting pressure from the tumor microenvironment. We hope that the Reviewer will appreciate the conceptual novelty of our findings.

Review COMMONS

3. Description of analyses that authors prefer not to carry out

Please include a point-by-point response explaining why some of the requested data or additional analyses might not be necessary or cannot be provided within the scope of a revision. This can be due to time or resource limitations or in case of disagreement about the necessity of such additional data given the scope of the study. Please leave empty if not applicable.

Initial revision plan:

As discussed in our reply to Reviewer #2, we lack the resources and methodology to carry out 13C-glucose tracing. Also, as discussed in our reply to this reviewer, for tracing glucose to cytokine expression we would need to connect lower glucose availability with quantitative changes in posttranslational modifications of histones and transcription factors with glucose-derived metabolites. We respectfully think that these experiments, while interesting, would be out of our reach and perhaps would not contribute substantially to other points of the manuscript raised by the Reviewer, which we think are more relevant to address.

Updated reply

A discussion about obstacles encountered, and alternative approaches to carry out metabolic analyses with TILs and assess the dependence on glucose of TILs biosynthetic capacity in high and low blood accessibility tumor regions, has been updated in our reply to Reviewer #2.

Dear Dr. López-Rodríguez,

Thank you for the submission of your revised manuscript to our editorial offices. I have already forwarded to you the reports I received from the three referees that I asked to re-evaluate your study. Please find them again below. After going through your preliminary point-by-points response (further revision plan), I have decided to proceed with the manuscript.

I would thus like to invite you to further revise your manuscript with the understanding that the concerns of the referees will be addressed in a final revised manuscript and/or in a final detailed point-by-point response, as indicated in your revision plan. Acceptance of your manuscript will depend on a positive outcome of a final round of review, involving referee #1. Please try to visualize T cell proximity to vessels together with in vivo Hoechst uptake, as suggested in your revision plan. Moreover, please do all the textual changes suggested and improve the clarity of the manuscript. It will, however, not be necessary to validate the conclusions with CD8 T cells or Th1 cells (as requested by referee #3), but please rewrite all partes related to the points of referee #3 as indicated in your revision plan. Moreover, please provide a detailed final p-b-p-response to the referee concerns and to the editorial requests below.

Editorial requests:

- Please provide the abstract written in present tense throughout.

- Please order the manuscript sections like this, using only these names:
Title page - Abstract (max. 175 words) - Keywords (up to five) - Introduction - Results - Discussion - Methods - Data availability section - Acknowledgements (please include here all the funding information) - Disclosure and Competing Interests Statement - References - Figure legends - Expanded View Figure legends

Thus, please add the funding information to the acknowledgements. Moreover, please make sure that all the funding information is also entered into the online submission system and that it is complete and similar to the one in the acknowledgement section of the manuscript text file. Presently, it seems that these grants are missing in the submission system: 10.13039/501100011033; the European Fund for Regional Development "ERDF/EU A way of making Europe"; a predoctoral fellowship of the Spanish Ministry of Science and Innovation: PRE2022-102056; (MICIU/AEI/10.13039/501100011033). Please check.

- We now use CRediT to specify the contributions of each author in the journal submission system. CRediT replaces the author contribution section. Please use the free text box to provide more detailed descriptions and do NOT provide your final manuscript text file with an author contributions section. See also our guide to authors (section 'Author contributions'): https://link.springer.com/journal/44319/submission-guidelines#cms-Revised-submissions

- Please check again that the number "n" for how many independent experiments were performed, their nature (biological versus technical replicates), the bars and error bars (e.g. SEM, SD) and the test used to calculate p-values is indicated in the respective figure legends (main, EV and Appendix figures). Please also check that all the p-values are explained in the legend, and that these fit to those shown in the figure. Please provide statistical testing where applicable. Please avoid the phrase 'independent experiment' but clearly state if these were biological or technical replicates. Please also indicate (e.g. with n.s.) if testing was performed, but the differences are not significant. In case n=2, please show the data as separate datapoints without error bars and statistics. See also:

https://link.springer.com/journal/44319/submission-guidelines#cms-Figure-and-data-presentation

If n<5, please show single datapoints for diagrams. It seems that presently some diagrams show only partial statistics or miss the 'n.s.'. Moreover:
Please note that the exact p values are not provided in the legends of figures 1B, C, D, E, H, J; 2A-C; 3A-D; 4C, D; 5B, C; 6A, B; 7A-C; EV1 A, C, D; EV2 E, F; EV3 A-C; EV4, EV5 B, D, E, H, G.
- Please indicate the statistical test used for data analysis in the legends of figures 3G, 4A.

- Please add the primer information (Appendix Table S1) to the Reagents & Tools Table and remove the Table from the Appendix. Please update any callouts. Moreover, please upload the Reagents and Tools Table as separate file only. Please remove the table from the manuscript text file.

- Please add Appendix Fig. S1 to one of the EV figures. In case, we can also accommodate a sixth EV figure. Then we do not need the Appendix. Please update the callouts and the EV Figure legends.

- Please remove the referee token from the Data Availability Section and make sure that the GEO dataset is public latest upon online publication of the paper.

- Please remove the 'Numerical data for Reviewer Response Fig. R2, specifically R2B' from the manuscript files. If these figures will be shown in the final manuscript, then please add these files to the respective source data folders.

- Please remove the 'address correspondence' information from present page 45. This should be indicated only on the title page.

- Thanks for providing the source data. Please provide a final source data checklist (created by you - not the old one sent by our source data coordinator) and add any new data that is added during final revision. Make sure that all links to externally deposited source data are updated and present in the final Data Availability Section. Please upload all the numerical source data to the submission system. Please upload the source data as one folder per main figure, grouping together all the files (separate excel files for each panel) for this figure (and ZIPed together), and one folder for all the EV Figures, grouping together all the files for each EV Figure in separate folders (and ZIPed together).

In addition, I would need from you uploaded separately:
- a short, two-sentence summary of the manuscript (not more than 35 words).
- two to four short (!) bullet points highlighting the key findings of your study (two lines each).
- a schematic summary figure as separate file that provides a sketch of the major findings (not a data image) in jpeg or tiff format (with the exact width of 550 pixels and a height of not more than 400 pixels) that can be used as a visual synopsis on our website.

I look forward to seeing the further revised version of your manuscript when it is ready. Please let me know if you have questions regarding the revision.

Yours sincerely,

Achim Breiling
Senior Editor
EMBO Reports

Referee #1:

The revised manuscript shows substantial improvement. While I do not doubt the data, I remain concerned about certain interpretations. The authors used high and low uptake of Hoechst and 2-NBDG to infer the proximity of T cells to blood vessels within tumors. However, the evidence provided does not convincingly support this assumption. For example, could the authors perform staining on tumor sections to demonstrate that T cells located near blood vessels indeed exhibit stronger Hoechst signals?

Moreover, 2-NBDG uptake can be influenced by cell-intrinsic factors. How can the authors be certain that the lower uptake observed is primarily due to limited access to blood vessels rather than intrinsic transporter expression or other metabolic factors? Does high intracellular 2-NBDG affect metabolism in cells? Additionally, it is unclear why some analyses were performed on cells sorted based on Hoechst uptake, while others used 2-NBDG. A more consistent approach across analyses would strengthen the conclusions.

Finally, although the ACSS2 data are compelling, I do not think they are sufficient to conclude that Hoechst-high cells rely on acetate as a major fuel for ATP production without supporting metabolic tracing evidence.

Overall, I found the manuscript challenging to follow. The figures were not always clearly explained, and in a few cases, I had to refer to the Methods section to understand the experimental design or treatments used.

Referee #2:

In their revised manuscript, Riera-Borrull et al. have addressed all my comments/concerns. The authors did significant work, improving their manuscript's impact and clarity. Thus, I recommend its publication in Embo Reports. Congratulations.

Referee #3:

I have the following major concerns:

1. The authors choose Th17 cells for monitoring their differentiation and effector function in the context of high or low glucose conditions, however, this is artificial since endogenous tumor infiltrating T cells barely harbor Th17 polarization phenotype though Th17 cells are largely considered to be highly glycolytic. In this regard, the authors need to validate the key conclusions with CD8 T cells or Th1 cells, which will make these findings more relevant.
2. It is not convincing to use 0.3mM glucose for interpretation of poorly perfused regions of tumor bed, which is far more complexed in vivo setting. I am concerned about the physiological or pathological relevance of the in vitro culture model.
3. The logic of the study is not very clear and the way of presenting data is not convincing.
4. A graphic abstract is missing and what is the key message the authors want to deliver??

Point-by-point response to Reviewers, manuscript EMBOR-2024-60965V3

Note about numbering of figures and panels within: throughout this reply to reviewers, we will refer to the figures with the numbering they have in this latest revision. New data added during this revision are shown in Figs 4A, 5, A to D, and EV3A. The rest of the data had already been shown in the previous version, but we have rearranged the order in which some of them are shown, in order to achieve a more fluid narrative. At the end of this reply we have included a list with the new and updated figures and their correspondence with those in the previous version.

Referee #1

1- The revised manuscript shows substantial improvement. While I do not doubt the data, I remain concerned about certain interpretations. The authors used high and low uptake of Hoechst and 2-NBDG to infer the proximity of T cells to blood vessels within tumors. However, the evidence provided does not convincingly support this assumption. For example, could the authors perform staining on tumor sections to demonstrate that T cells located near blood vessels indeed exhibit stronger Hoechst signals?

We appreciate the positive assessment of our revised manuscript and its data by the Referee. We also understand their concern about the extent to which the uptake of Hoechst and 2-NBDG reflects proximity to blood vessels. As cited in our manuscript, Hoechst injected intravenously before isolating the tumor has been used to identify cells with better or poorer accessibility to vessels (Huang et al, 2012, *Proc Natl Acad Sci USA* 109: 17561–17566; Chaplin et al, 1985, *Br J Cancer* 51: 569–572; Kumar et al, 2019, *Cell Metabolism*, 30: 201-211.e6; Kumar et al, 2020, *Bio-protocol* 10: e3628; Zheng et al, 2018, *J Clin Invest* 128: 2104–2115), and tumor regions with poorer Hoechst perfusion have been shown to overlap with hypoxic, glucose-poor niches (Schroeder et al, 2005, *Cancer Res*. 65: 5163-5171). Nonetheless, we concur with the Referee that directly visualizing T cell proximity to vessels together with *in vivo* Hoechst uptake in tumor sections would provide much more convincing evidence in support of our flow cytometry data. As we did not have experience with this methodology, we have collaborated with another group to carry out these experiments, and finally answered this question. Our new results show that the Hoechst signal was highest near vessels (identified by the endothelial cell marker CD31) and decreased steeply with distance (Figs 5, C and D, and EV3A). Likewise, CD4 and CD8 TILs showed a clear and significant inverse correlation between Hoechst uptake and distance from blood vessels, and TILs 20 to 40 µm away from the nearest vessel displayed a substantial lower uptake of Hoechst than those in the near vicinity of the vessel (Fig. 5D).

We would also like to comment that while setting up these experiments we encountered one unexpected problem that we had to troubleshoot. We found that the signal (intensity and concentration in perivascular regions) of *in vivo*-injected Hoechst was preserved in the frozen tissue blocks for up to at least one week, but it diffused and washed away substantially if the time from thawing the frozen tumor to immunostaining, mounting and imaging was longer than 20-24 hours, so that the Hoechst staining that remained was very weak and essentially homogeneous across the tissue section without distinction between regions near or far from vessels. This can be really limiting since most protocols for antibody staining recommend overnight incubations, and we have included this observation in our material and methods section. Among the works we used to set up this method, we only found the need for shortening the staining time to minimize Hoechst diffusion mentioned in Schroeder et al, 2005, *Cancer Res*. 65: 5163-5171.

2- Moreover, 2-NBDG uptake can be influenced by cell-intrinsic factors. How can the authors be certain that the lower uptake observed is primarily due to limited access to blood vessels rather than intrinsic transporter expression or other metabolic factors?

We agree with the Referee that the uptake of 2-NBDG can be influenced by factors besides proximity to blood vessels. Our initial hypothesis was that 2-NBDG uptake by TILs *in vivo* would reflect their capacity to capture glucose, which would depend on two main parameters, glucose (or 2-NBDG) availability in the fluid surrounding the T cells, and their intrinsic metabolic and functional characteristics that would cause them to use more or less glucose.

After this starting hypothesis, the chronological order of our 2-NBDG experiments began with the original RNA-seq analysis and further validations (now shown in Figs 3, B to H, and EV2, C and D). Fig 4, identifying myeloid cells in the tumor as producers of IFN-I, was added later during revision, but used 2-NBDG for consistency with Figs 3 and EV2.

While we were progressing through these analyses, a paper by Linda Sinclair and colleagues was published showing that glucose and 2-NBDG are captured by T cells through different mechanisms, and that 2-NBDG uptake cannot be considered equivalent to glucose uptake (Sinclair et al, 2020, *Immunometabolism*, 2: e200029). Although recent works have still used 2-NBDG to assess glucose uptake (for instance, Lopes et al, 2021. *Nat Immunol* 22:179-192; Watson et al, 2021. *Nature* 591. 645-651), several articles have reached similar conclusions as Sinclair's (Reinfeld et al, 2021. *Nature* 593: 282–288; Pelgrom et al, 2023. Cell Reports 42: 112828). Since Sinclair's work also reported that higher 2-NBDG uptake could correlate with more metabolically active T cells, something to be expected from TILs with better access to blood, we were uncertain about whether the uptake of 2-NBDG by TILs could parallel that of glucose or was affected by other variables. We have elaborated this point in the revised manuscript (lines 289-297).

Here we reasoned that our *in vivo* 2-NBDG uptake experiments could at least be informative about the better or poorer accessibility of TILs to blood-transported cargo and nutrients. To test this, we set up the Hoechst 33342 uptake experiments, and found a significant correlation between the uptake of Hoechst and 2-NBDG in leukocytes and TILs within the same tumor (Fig 5F). Then we confirmed that the enhanced IFN-I response signature that we had identified in TILs with poor 2-NBDG uptake was also found in TILs with poor Hoechst uptake (mRNA of CXCL10, IFIT1, STAT1) (Fig EV3, B and C), indicating that this signature was characteristic of effector TILs in poorly perfused tumor regions. On the other hand, variations in the levels of other mRNAs (for instance, IFNγ, CTLA4 and FOXP3) between 2-NBDG ^{Lo} and ^{Hi} TILs were not consistent between Hoechst ^{Lo} and ^{Hi} cells (new Fig EV3E). In line with the Reviewer question, we discuss in our manuscript that whereas Hoechst uptake could mainly depend on perfusion, the uptake of 2-NBDG could also be affected by changes in TIL polarization and activation state in addition to localization (commented in manuscript lines 350-354). We also discuss that *in vivo* 2-NBDG ^{Lo} TILs exhibit some similarities with glucose-restricted T lymphocytes in our *in vitro* assays and in earlier works (Ho et al, 2015, *Cell* 162: 1217-1228; Safford et al, 2005, *Nat Immunol*, 6: 472-480; Watson et al, 2021, *Nature*, 591: 645-651) (discussed in manuscript lines 493-497), suggesting that 2-NBDG ^{Lo} TILs could have an attenuated activation state. In this regard, though, we think that it is likely that the phenotype of TILs with poor uptake of blood-delivered probes will result from their being exposed to a complex metabolic microenvironment, not one just low in glucose (discussed in manuscript lines 497-500).

Regarding expression of glucose transporters, our RNA-seq results showed that effector CD4 and CD8 TILs predominantly expressed Glut1 (*Slc2a1*) and Glut3 (*Slc2a3*) at comparable levels, with much lower expression of Glut6 (*Slc2a6*) and Glut8 (*Slc2a8*), in agreement with a previous analysis of glucose transporter expression in non-tumor T cells (Macintyre et al, 2014, *Cell Metabolism*, 20: 61-72). Here we observed that Glut1 expression was about 15% lower and Glut3 50% lower in 2-NBDG ^{Hi}

CD4 and CD8 TILs with respect to their 2-NBDG ^{Lo} counterparts. We have included this information in a table below. We also did RT-qPCR analyses with independent samples and found similar Glut1 expression between 2-NBDG ^{Hi} and ^{Lo} TILs, both for CD4 and CD8, and comparable mRNA levels as well for glycolysis enzymes PFKP, GAPDH, PKM2, LDHA and LDHB. These analyses have been not been included in the manuscript, but if the editor and reviewers consider it useful, we could incorporate the RNA-seq data for the glucose transporters and the RT-qPCR of the glycolysis enzymes in one of the figures. In summary, our observations based on mRNA expression data suggest that TILs with lower 2-NBDG uptake would not have impaired expression of glucose

						RNA-seq reads			
chromosome	start position	end position	description	gene name	Protein	CD4 2NBDG ^{Lo} cDNA	CD4 2NBDG ^{Hi} cDNA	CD8 2NBDG ^{Lo} cDNA	CD8 2NBDG ^{Hi} cDNA
4	119108711	119137983	solute carrier family 2 (facilitated glucose transporter), member 1 [Source:MGI Symbol;Acc:MGI:95755]	*Slc2a1*	Glut1	1292	1073	511	438
2	165503787	165519917	solute carrier family 2 (facilitated glucose transporter), member 10 [Source:MGI Symbol;Acc:MGI:2156687]	*Slc2a10*	Glut10	0	0	0	2
10	22645011	22704285	solute carrier family 2 (facilitated glucose transporter), member 12 [Source:MGI Symbol;Acc:MGI:3052471]	*Slc2a12*	Glut12	1	1	0	8
15	91267696	91573261	solute carrier family 2 (facilitated glucose transporter), member 13 [Source:MGI Symbol;Acc:MGI:2146030]	*Slc2a13*	Glut13	2	13	2	19
3	28697903	28731359	solute carrier family 2 (facilitated glucose transporter), member 2 [Source:MGI Symbol;Acc:MGI:1095438]	*Slc2a2*	Glut2	2	1	2	29
6	122727809	122801640	solute carrier family 2 (facilitated glucose transporter), member 3 [Source:MGI Symbol;Acc:MGI:95757]	*Slc2a3*	Glut3	1085	633	749	386
11	69942539	69948188	solute carrier family 2 (facilitated glucose transporter), member 4 [Source:MGI Symbol;Acc:MGI:95758]	*Slc2a4*	Glut4	0	0	1	5
4	150119283	150144169	solute carrier family 2 (facilitated glucose transporter), member 5 [Source:MGI Symbol;Acc:MGI:1928369]	*Slc2a5*	Glut5	0	1	0	3
2	27021363	27027998	solute carrier family 2 (facilitated glucose transporter), member 6 [Source:MGI Symbol;Acc:MGI:2443286]	*Slc2a6*	Glut6	37	51	59	52
2	32972990	32982083	solute carrier family 2, (facilitated glucose transporter), member 8 [Source:MGI Symbol;Acc:MGI:1860103]	*Slc2a8*	Glut7	73	47	8	15
5	38349273	38503143	solute carrier family 2 (facilitated glucose transporter), member 9 [Source:MGI Symbol;Acc:MGI:2152844]	*Slc2a9*	Glut8	66	27	47	35

transporters or glycolysis enzymes.

3- Does high intracellular 2-NBDG affect metabolism in cells?

We have not found studies analyzing this, except an early work in *E. coli* showing that 2-NBDG is phosphorylated intracellularly, which indicates that at least in bacteria it is metabolized (Yoshioka et al, 1996, *Bioscience, Biotechnology, and Biochemistry*. 60: 1899-1901). Early on in our study we did 2-NBDG labeling experiments with *in vitro*-activated CD4 T cells (spleen and lymph nodes), in which we incubated cells at 10 x 10^6 cells/ml with up to 45 µg/ml (131 µM) 2-NBDG during 1h, and did not observe short-term loss of viability in comparison with unlabeled T cells. However, in longer tests (16-24 hours), 2-NBDG-loaded T cells did not proliferate well. These were preliminary experiments and have not been included in the manuscript. In our *in vivo* assays shown in the paper, T cells were immediately processed after euthanizing the mice injected with 2-NBDG 10 minutes before, and we did not do *ex vivo* experiments that required culture of 2-NBDG-labeled cells, therefore we would not expect much impact of a short *in vivo* 2-NBDG labeling on TILs.

4- Additionally, it is unclear why some analyses were performed on cells sorted based on Hoechst uptake, while others used 2-NBDG. A more consistent approach across analyses would strengthen the conclusions.

We agree with the Reviewer on the need of making the narrative of our manuscript clearer. A similar point was also raised by Reviewer #3.

After our initial findings that T cells activated in culture could maintain substantial functionality under very low glucose (Figs 1, 2 and EV1), we decided to use the uptake of 2-NBDG as an approximation

to identify TILs with higher or lower glucose uptake capacity *in vivo*. However, as discussed above in our reply to this Reviewer's question 2, by the time we had done the RNA-seq analysis with 2-NBDG ^{Hi} and ^{Lo} TILs, Sinclair's paper and others showed that the uptake of 2-NBDG by T cells was not equivalent to glucose uptake. At this point we were uncertain about how to interpret our results with 2-NBDG in TILs, and reasoned that perhaps what we were seeing reflected differences in TILs accessibility to blood circulation.

We then found a significant within-tumor correlation between Hoechst 33342 and 2-NBDG in TILs (Fig 5F), and confirmed an enhanced IFN-I response signature both in TILs with poor 2-NBDG uptake and TILs with poor Hoechst uptake (Fig EV3B). Our assumption from these results was that at least some characteristics of TILs with poor 2-NBDG uptake *in vivo*, such as an enhanced IFN-I response, could define TILs in poorly perfused tumor regions. In this regard, our new microscopy results in Fig 5, C and D confirm a highly significant inverse correlation between Hoechst uptake in CD4 and CD8 TILs and their distance from blood vessels. Taking into account that Hoechst is a widely accepted indicator of tumor perfusion, and that the uptake of 2-NBDG, despite being influenced by blood accessibility, could also respond to other as yet undefined variables, we decided to continue our study with Hoechst. In this way we could characterize TILs from different tumor regions (better or poorly accessible to blood) whose phenotype would result from their being exposed to a complex metabolic microenvironment, not one just low in glucose.

The way we had narrated our manuscript after the extensive previous revision did not follow this chronological order and combined experiments with 2-NBDG and Hoechst without adequate explanation. We think that this could have made it hard to follow, and have now rearranged the content of Figs 3 through 6, including new results and narrating them with a clearer storyline. Also, at the end of the point-by-point reply to reviewers we have provided a list with the new order of figures, including the new results, and the correspondence of our updated figures with those in the previous version. We hope that the Reviewer will find that our revised manuscript has improved in clarity and consistency.

***5- Finally, although the ACSS2 data are compelling, I do not think they are sufficient to conclude that Hoechst-high cells rely on acetate as a major fuel for ATP production without supporting metabolic tracing evidence*.**

We appreciate that the Referee found our new data compelling. However, it was not our conclusion that Hoechst ^{Hi} cells rely on acetate as a major fuel for ATP production, and we fear that perhaps we might have confused the Referee with the diagram in Fig EV5A, which was adapted from Qiu et al, 2019, Cell Rep. 27: 2063-2074.e5. We have now rephrased the description of this set of results to make them clearer, and written in the figure legend that the diagram has been adapted from Qiu et al. Our findings with the ACCS2 inhibitors are summarized below.

Our experiments with TILs stimulated *ex vivo* (without separating Hoechst ^{Hi} or ^{Lo} cells) indicate that ACSS2 can contribute to the expression of IFNγ, other cytokines, and several IFN-stimulated genes (ISGs) in effector TILs activated via T cell receptor and type I IFNs (Fig 7), but our *in vivo* results with the ACSS2 inhibitors were rather modest to conclude any major role of ACSS2 in one or another population. We also discuss other works in the literature differing in their results about the contribution of ACSS2 to TIL function (Qiu et al, 2019, *Cell Rep*. 27: 2063-2074.e5; Miller et al, 2023, *Nat Cancer*, 4: 1491–1507; Balmer et al, 2020, *Cell Metab*. 32: 457-467.e5).

Regarding ATP production, we did not test acetate as a fuel, and our analysis of ATP-contributing pathways showed that both Hoechst ^{Hi} and ^{Lo} effector CD4 and CD8 TILs exhibit fundamentally glucose-dependent and respiration-independent ATP production (Fig 8B). We also found that TILs in LLC tumors were better producers of IFNγ than IL-17A, which is a quite interesting result in view of an

earlier work (Lopes et al 2021. *Nat Immunol* 22:179-192) that showed that IFNγ-producing antitumor γδ T cells are markedly glycolytic, whereas IL-17-producing pro-tumor γδ T cells depend on glucose and mitochondrial respiration.

***6- Overall, I found the manuscript challenging to follow. The figures were not always clearly explained, and in a few cases, I had to refer to the Methods section to understand the experimental design or treatments used*.**

As commented above, we agree with the Referee that the clarity of our manuscript could be improved, and we thank them again for their clear and helpful comments. We hope that the Reviewer will find that our revised version has been significantly improved.

Referee #2

***In their revised manuscript, Riera-Borrull et al. have addressed all my comments/concerns. The authors did significant work, improving their manuscript's impact and clarity. Thus, I recommend its publication in EMBO Reports. Congratulations*.**

We are grateful to the Reviewer for their very positive comments and recommending publication in EMBO reports. We hope that they will also appreciate positively the changes in this last revision.

Referee #3

***1- The authors choose Th17 cells for monitoring their differentiation and effector function in the context of high or low glucose conditions, however, this is artificial since endogenous tumor infiltrating T cells barely harbor Th17 polarization phenotype though Th17 cells are largely considered to be highly glycolytic. In this regard, the authors need to validate the key conclusions with CD8 T cells or Th1 cells, which will make these findings more relevant*.**

i) We agree with the Referee that Th17 cells may not be a main phenotype among TILs in our LLC tumor model, as already suggested by our RNA-seq analysis (see table below showing the data for Th17 genes versus the much higher expression of the Th1 gene *Ifng*), and our *ex vivo* stimulation assays with TILs (Fig 3A and EV2B, and lines 218-219 of the revised manuscript).

Nonetheless, Th17 cells exist in tumors, although different studies have found them to have divergent, anti-tumor to pro-tumor, functions (Váraljai et al, 2023, *Nature Cancer*, 4: 1292–1308; and references therein; Lopes et al, 2021, *Nature Immunology* 22: 179–192). Our choice of using Th17-promoting stimulation at the beginning of our work to study effector T cells under low glucose was because, as the Referee correctly indicates, Th17 cells are highly glycolytic. Glucose is a key nutrient for T cell function and its availability is reduced in the TME (Hirayama et al, 2009, *Cancer Res*. 69: 4918–4925; Schroeder et al, 2005, *Cancer Res*. 65: 5163-5171; Sullivan et al, 2019, *eLife* 8: e44235). The reduced availability of glucose in the TME has been reported to contribute to weakening anti-tumor T cell capacity (Chang et al, 2013, *Cell* 153: 1239–1251; Ho et al, 2015, *Cell* 162: 1217–1228).

					RNA-seq reads			
chromosome	start position	end position	description	gene name	CD4 2NBDG ^{Lo} cDNA	CD4 2NBDG ^{Hi} cDNA	CD8 2NBDG ^{Lo} cDNA	CD8 2NBDG ^{Hi} cDNA
10	118441046	118445892	interferon gamma [Source:MGI Symbol;Acc:MGI:107656]	*Ifng*	2778	5169	4060	3145
1	20730905	20734496	interleukin 17A [Source:MGI Symbol;Acc:MGI:107364]	*Il17a*	0	45	1	1
18	61684887	61692538	interleukin 17B [Source:MGI Symbol;Acc:MGI:1928397]	*Il17b*	0	0	0	7
8	122422020	122423639	interleukin 17C [Source:MGI Symbol;Acc:MGI:2446486]	*Il17c*	0	2	0	1
14	57524777	57543166	interleukin 17D [Source:MGI Symbol;Acc:MGI:2446510]	*Il17d*	0	0	1	1
1	20777146	20790617	interleukin 17F [Source:MGI Symbol;Acc:MGI:2676631]	*Il17f*	1	22	21	23
3	37222759	37232636	interleukin 21 [Source:MGI Symbol;Acc:MGI:1890474]	*Il21*	27	325	0	23
10	118204942	118210047	interleukin 22 [Source:MGI Symbol;Acc:MGI:1355307]	*Il22*	0	24	0	4
6	67422932	67491855	interleukin 23 receptor [Source:MGI Symbol;Acc:MGI:2181693]	*Il23r*	0	113	0	31

ii) As to validating key conclusions in Th17, Th1 and Th0 stimulation, we did not limit our glucose-sensitivity assays to Th17 stimulation, but also included experiments with CD4 T cells in Th1 stimulatory conditions (Fig EV1, C and D), and tested effector CD4 TILs sorted from tumors for their induction of IL-17A, IFNγ, IL-2 and TNFα (Fig 3A and EV2B). In the experiments with TILs we used unpolarized stimulation, “Th0”, so that cells could better reveal potential biases in cytokine production that would be masked if we added polarizing cytokines. Indeed, we see that effector CD4 TILs display a clear Th1-like profile (IFNγ, TNFα, IL-2) upon *ex vivo* Th0 stimulation.

Importantly, in these experiments we found a common theme between spleen/lymph node CD4 T cells stimulated in Th17 conditions (Figs 1, A and D, and EV1A) and CD4 TILs stimulated in unpolarized conditions (Th0) (Fig 3A and EV2B), in that the accumulated production of IL-17A, IFNγ, and TNFα protein over 48 hours in both cell types was lower under 0.3 mM than 5 mM glucose. Nonetheless, in both types of experiments, cells still produced substantial levels of these cytokines when stimulated in 0.3 mM glucose as compared to unstimulated conditions. With regard to cytokine mRNA, the samples were collected at 48 hours, at the same time as the supernatants for measuring the accumulated cytokines, and it is possible that mRNA levels could have peaked at a different time and we might have seen an incomplete picture without a time course analysis.

Finally, while we did not quantify cytokine protein in Th1-stimulated spleen/lymph node CD4 T cells (only mRNA, Fig EV1C), we tested the glucose dependence for ATP production in these cells stimulated as Th17 and Th1 (Fig EV1D), and also in effector TILs (CD4 and CD8) freshly isolated from better and poorly perfused-tumor regions (Fig 8B). These assays showed that all had a marked glucose dependence for energy production.

Therefore, our *ex vivo* and *in vitro* stimulation assays with TILs, and also spleen and lymph node CD4 T cells, have covered Th17, Th1 and Th0 stimulatory conditions. We believe that these experiments support the conclusion that activated T cells from different sources can induce substantial cytokine production and maintain glucose-dependent ATP under severe glucose limitation.

iii) Regarding the comparison of CD4 and CD8 T cells, we summarize below the results that support similar conclusions for both cell types.

- Expression of surface PD-1 and LAG-3 enhanced in Hoechst ^{Hi} versus ^{Lo} effector TILs, both in CD4 and CD8 cells (Fig 6C). In the same experiments, TIM-3 expression was found to be enhanced in Hoechst ^{Hi} versus ^{Lo} CD8 effector TILs, but constant in CD4 TILs (Fig 6C).

- Expression of IFNγ protein upon short (4h) stimulation of freshly sorted Hoechst ^{Hi} and ^{Lo} CD4 and CD8 effector TILs shows that CD4 and CD8 cells were comparably competent to express this cytokine (Fig 6, D and E). In the same experiments, we found a greater expression of granzyme B in CD8 than CD4 TILs, as expected (Fig 6, D and E).

- Lower overall metabolic activity in Hoechst ^{Lo} versus ^{Hi} (more and less blood-accessible respectively) effector TILs, both in CD4 and CD8 cells, inferred from their reduced protein translation rate (Fig 8A). Our results also show that both Hoechst ^{Hi} and ^{Lo} effector CD4 and CD8 TILs exhibit fundamentally glucose-dependent and respiration-independent energy metabolism (Fig 8B). As discussed in our manuscript, this result is indeed quite interesting in view of an earlier work (Lopes et al 2021. *Nat Immunol* 22:179-192) that showed that among tumor-infiltrating γδ T cells, those producing IFNγ were fundamentally glucose-dependent, whereas IL-17A-expressing cells were strongly dependent on mitochondrial respiration.

- RNA-seq and independent validation experiments, including IFNAR1-deficient mice, show enhanced IFN-responses in both 2-NBDG ^{Lo} and Hoechst ^{Lo} CD4 and CD8 effector TILs with respect to their counterparts with higher probe uptake (Figs. 3, F to H, and EV3, B and C).

- Comparable glucose dependence of IFN-I responses in spleen and lymph node CD4 and CD8 T cells stimulated *in vitro* (Fig EV4).

Altogether, we believe that our experiments cover the comparison of different relevant parameters in CD4 and CD8 TILs to support that both cell types in poorly perfused (Hoechst ^{Lo}) tumor regions have an enhanced IFN-I response signature, also the capacity to rapidly induce robust IFNγ production, mostly comparable between CD4 and CD8 effector TILs. Both CD4 and CD8 effector TILs in poorly perfused regions (Hoechst ^{Lo}) also have reduced metabolic activity, but are as fundamentally glucose-dependent for energy production as their counterparts in better perfused (Hoechst ^{Hi}) regions.

2- It is not convincing to use 0.3mM glucose for interpretation of poorly perfused regions of tumor bed, which is far more complexed in vivo setting. I am concerned about the physiological or pathological relevance of the in vitro culture model.

We entirely agree with the Referee that lowering glucose to 0.3 mM in an otherwise complete medium is not equivalent to the microenvironment encountered by T cells in the tumor. After our initial *in vitro* experiments with spleen and lymph node T cells, we found it very intriguing that they were capable of substantial functionality under very low glucose concentrations, and moreover, were still prominently glucose-dependent to maintain ATP levels. As we had mentioned in our manuscript, the 0.3 mM glucose concentration we used is in the range (0.1 – 1 mM) measured in solid tumors (Hirayama et al, 2009, *Cancer Res*. 69: 4918–4925; Chang et al, 2013, *Cell* 153: 1239–1251; Ho et al, 2015, *Cell* 162: 1217–1228), and in inflamed tissues in various pathological conditions (Hurd et al, 1979, *Ann Rheum Dis* 38 Suppl 1: 55–58; Pettersson et al, 1982, *Thorax* 37: 354–361; Valdés et al, 2010, *Respir Med* 104: 1211–1217). However, a culture medium with reduced glucose concentration is not equivalent to the nutrient and metabolite microenvironment encountered by T cells in the tumor, and this is why we decided to compare TILs from poorly and well-perfused tumor regions.

As correctly pointed out by the Reviewer, that the TME might be low in glucose does not imply that glucose is the only metabolite affected or that the phenotype of TILs can be interpreted as if it was the result of only the reduction in glucose levels. This needed to be clarified in our manuscript to avoid confusing readers about our interpretation, and in the revised discussion of our manuscript we have written (manuscript lines 493 – 501) that "*Nonetheless our results indicate that some characteristics of effector TILs with poor uptake of 2-NBDG, such as the downregulation of cell proliferation and cell cycle-related gene hallmarks, remind of T lymphocytes with restricted access to blood-transported*

glucose and other nutrients (Ho et al, 2015; Safford et al, 2005; Watson et al, 2021). In this regard, it is likely that the phenotype we have seen in TILs with poor uptake of blood-delivered probes could result from their being exposed to a complex metabolic microenvironment (Sullivan et al, 2019; Lim et al, 2020; Chapman & Chi, 2022; Reinfeld et al, 2021), and not one just low in glucose". We hope that the new discussion makes this point clearer.

3. The logic of the study is not very clear and the way of presenting data is not convincing.

We understand the Reviewer's comment and agree with them on that our manuscript needed more clarity. Reviewer #1 also made a similar comment. One element that probably made our narrative not as clear as it should is that we began this study with a relatively simple question and experimental system and then we moved on to a complex *in vivo* scenario, for which our initial *in vitro* data were a partial approximation. Also, our previous revision to address the numerous comments by the reviewers, led to the inclusion of a substantial amount of new experimental data in our manuscript, and the order and way in which we described them was not as clear as it could have been.

After our initial observation that T cells in culture could maintain substantial functionality under very low glucose (Figs 1, 2 and EV1), we thought of studying T cells that could be distinguished by their *in vivo* higher or lower uptake of glucose, and used the deoxyglucose analog 2-NBDG to identify them in the tumor microenvironment. However, after having committed considerable effort to this approach and obtaining original results comparing 2-NBDG ^{Hi} and ^{Lo} TILs, an article by Linda Sinclair and colleagues showed that the uptake of 2-NBDG by T cells was not equivalent to the uptake of glucose uptake (Sinclair et al, 2020, *Immunometabolism*, 2: e200029). In light of those findings, we explored the possibility that some of the differences we were seeing between 2-NBDG ^{Hi} and ^{Lo} TILs reflected differences in their accessibility to blood. We found a significant within-tumor correlation between Hoechst 33342 and 2-NBDG uptake in TILs (Fig 5F), and now our new microscopy results in Fig 5, C and D, confirm a highly significant inverse correlation between Hoechst uptake in CD4 and CD8 TILs and their distance from blood vessels. From these results we reasoned that at least some characteristics of TILs with poor 2-NBDG and Hoechst uptake *in vivo*, such as an enhanced IFN-I response (Fig EV3, B and C) could define TILs in poorly perfused tumor regions, and decided to continue our experiments with Hoechst. In this way we could characterize TILs from different tumor regions (better or poorly accessible to blood) whose phenotype would result from their being exposed to a complex metabolic microenvironment, not one just low in glucose.

The narrative of our previous manuscript version after its extensive revision did not follow the chronological sequence described above, and combined experiments with 2-NBDG and Hoechst without adequate explanation, which made our manuscript hard to follow. We have now rearranged the content of Figs 3 through 6, including new results and narrating them in what we think is a better reasoned and more fluid sequence. Also, at the end of this reply we have included a list with the new and updated figures and their correspondence with those in the previous version. We hope that the Reviewer will find that our revised manuscript has improved in clarity and consistency.

4. A graphic abstract is missing and what is the key message the authors want to deliver?

We apologize about this. Indeed, the graphic abstract was missing, and is now included in the revised version. The key message in our manuscript is that tumor-infiltrating effector T lymphocytes in poorly perfused tumor regions are intrinsically competent to respond to stimulation comparably to TILs in better perfused regions, exhibit an enhanced type I IFN response signature supported by local myeloid cells, and unexpectedly, have reduced expression of immune checkpoint and Treg-associated markers. We also describe that TILs with poor blood accessibility have reduced biosynthetic activity in comparison with highly accessible TILs, but both depend fundamentally on glucose for their energy

metabolism. We trust that the Reviewer will appreciate that these original findings help advance our understanding of the different functional features of different TILs subsets.

We again thank the Reviewer for their helpful comments, and hope that they will appreciate the improvement to our manuscript and find the revised manuscript satisfactory.

EMBOR-2024-60965V3_New order of figures

Updated Figures		Correspondence with previous version	
Figure number	Figure panel	Figure number	Figure panel
Fig 1	A	Fig 1	A
	B		B
	C		C
	D		D
	E		E
	F		F
	G		G
	H		H
	I		I
	J		J
Fig 2	A	Fig 2	A
	B		B
	C		C
Fig 3	A	Fig 3	A
	B		E
	C		F
	D		G
	E		H
	F	Fig 4	A
	G		B
	H		C
Fig 4	A		
	B	Fig 5	A
	C		B
Fig 5	A		
	B		
	C		
	D		
	E	EV2	B
	F	Fig 5	C
Fig 6	A	EV2	D
	B	Fig 3	B
	C		C
	D		D
	E	EV3	C
Fig 7	A	Fig 6	A
	B		B
Fig 8	A	Fig 7	A
	B		B
EV1	A	EV1	A
	B		B
	C		C
	D		D
EV2	A	EV2	A
	B		F
	C		C
	D	Fig 4	D
EV3	A		
	B	Appendix Fig S1	A
	C		B
	D	Fig 5	C
	E	EV3	A + B
EV4	1 single panel	EV4	1 single panel
EV5	A	EV5	A
	B		B
	C		C
	D		D
	E		E
	F		F
	G		G

Color code
Same as in previous version
New results or data added
Diagram of experiment added
Order/numbering of panels/figure rearranged

Prof. Cristina López-Rodríguez
Universitat Pompeu Fabra
Medicine and Life Sciences
Carrer Dr Aiguader 88
Barcelona, Barcelona 08003
Spain

Dear Prof. López-Rodríguez,

Thank you for the submission of your further revised manuscript to our editorial offices. I have now received the report from the referee that was asked to re-evaluate the study, you will find below. As you will see, the referee now fully supports the publication of the study in EMBO reports.

I am thus very pleased to accept your manuscript for publication in the next available issue of EMBO reports. Thank you for your contribution to our journal.

Your manuscript will be processed for publication by EMBO Press. It will be copy edited and you will receive page proofs prior to publication. Please note that you will be contacted by Springer Nature Author Services to complete licensing and payment information.

You may qualify for financial assistance for your publication charges - either via a Springer Nature fully open access agreement or an EMBO initiative. Check your eligibility: https://link.springer.com/journal/44319/how-to-publish-with-us

Should you be planning a Press Release on your article, please get in contact with embo_production@springernature.com as early as possible in order to coordinate publication and release dates.

If you have any questions, please do not hesitate to contact the Editorial Office. Thank you for your contribution to EMBO Reports.

Yours sincerely,

Achim Breiling
Senior Editor
EMBO Reports

Referee #1:

I appreciate the authors' effort to address all my comments. The manuscript has been substantially improved. I have no other requests.

>>> Please note that it is EMBO Reports policy for the transcript of the editorial process (containing referee reports and your response letter) to be published as an online supplement to each paper. If you do NOT want this, you will need to inform the Editorial Office via email immediately. More information is available here: https://link.springer.com/partners/embo-press/editorial-policies#Peer%20review

EMBO Press Author Checklist

Corresponding Author Name: Cristina López-Rodríguez	
Journal Submitted to: EMBO Reports	
Manuscript Number: EMBOR-2024-60965V3	

USEFUL LINKS FOR COMPLETING THIS FORM
The EMBO Journal - Author Guidelines
EMBO Reports - Author Guidelines
Molecular Systems Biology - Author Guidelines
EMBO Molecular Medicine - Author Guidelines

Reporting Checklist for Life Science Articles (updated January
This checklist is adapted from Materials Design Analysis Reporting (MDAR) Checklist for Authors. MDAR establishes a minimum set of requirements in transparent reporting in the life sciences (see Statement of Task: 10.31222/osf.io/9sm4x). Please follow the journal's guidelines in preparing your
Please note that a copy of this checklist will be published alongside your article.

Abridged guidelines for figures
1. Data
The data shown in figures should satisfy the following conditions:
- the data were obtained and processed according to the field's best practice and are presented to reflect the results of the experiments in an accurate and unbiased manner.
- ideally, figure panels should include only measurements that are directly comparable to each other and obtained with the same assay.
- plots include clearly labeled error bars for independent experiments and sample sizes. Unless justified, error bars should not be shown for technical
- if n<5, the individual data points from each experiment should be plotted. Any statistical test employed should be justified.
- Source Data should be included to report the data underlying figures according to the guidelines set out in the authorship guidelines on Data

2. Captions
Each figure caption should contain the following information, for each panel where they are relevant:
- a specification of the experimental system investigated (eg cell line, species name).
- the assay(s) and method(s) used to carry out the reported observations and measurements.
- an explicit mention of the biological and chemical entity(ies) that are being measured.
- an explicit mention of the biological and chemical entity(ies) that are altered/varied/perturbed in a controlled manner.
- the exact sample size (n) for each experimental group/condition, given as a number, not a range;
- a description of the sample collection allowing the reader to understand whether the samples represent technical or biological replicates (including how many animals, litters, cultures, etc.).
- a statement of how many times the experiment shown was independently replicated in the laboratory.
- definitions of statistical methods and measures:
  - common tests, such as t-test (please specify whether paired vs. unpaired), simple χ2 tests, Wilcoxon and Mann-Whitney tests, can be unambiguously identified by name only, but more complex techniques should be described in the methods section;
  - are tests one-sided or two-sided?
  - are there adjustments for multiple comparisons?
  - exact statistical test results, e.g., P values = x but not P values < x;
  - definition of 'center values' as median or average;
  - definition of error bars as s.d. or s.e.m.

Please complete ALL of the questions below.
Select "Not Applicable" only when the requested information is not relevant for your study.

Materials

Newly Created Materials	Information included in the manuscript?	In which section is the information available? (Reagents and Tools Table, Materials and Methods, Figures, Data Availability Section)
New materials and reagents need to be available; do any restrictions apply?	Not Applicable	

Antibodies	Information included in the manuscript?	In which section is the information available? (Reagents and Tools Table, Materials and Methods, Figures, Data Availability Section)
For **antibodies** provide the following information:<br>- Commercial antibodies: RRID (if possible) or supplier name, catalogue number and or/clone number<br>- Non-commercial: RRID or citation	Yes	Reagents and Tools Table

DNA and RNA sequences	Information included in the manuscript?	In which section is the information available? (Reagents and Tools Table, Materials and Methods, Figures, Data Availability Section)
**Short novel DNA or RNA including primers, probes**: provide the sequences.	Yes	Reagents and Tools Table

Cell materials	Information included in the manuscript?	In which section is the information available? (Reagents and Tools Table, Materials and Methods, Figures, Data Availability Section)
**Cell lines:** Provide species information, strain. Provide accession number in repository **OR** supplier name, catalog number, clone number, and/**OR** RRID.	Yes	Materials and Mehods
**Primary cultures:** Provide species, strain, sex of origin, genetic modification status.	Yes	Materials and Mehods
Report if the cell lines were recently **authenticated** (e.g., by STR profiling) and tested for mycoplasma contamination.	Yes	Materials and Mehods

Experimental animals	Information included in the manuscript?	In which section is the information available? (Reagents and Tools Table, Materials and Methods, Figures, Data Availability Section)
**Laboratory animals or Model organisms:** Provide species, strain, sex, age, genetic modification status. Provide accession number in repository **OR** supplier name, catalog number, clone number, **OR** RRID.	Yes	Materials and Methods
**Animal observed in or captured from the field:** Provide species, sex, and age where possible.	Not Applicable	
Please detail **housing and husbandry conditions**.	Yes	Materials and Methods

Plants and microbes	Information included in the manuscript?	In which section is the information available? (Reagents and Tools Table, Materials and Methods, Figures, Data Availability Section)
**Plants:** provide species and strain, ecotype and cultivar where relevant, unique accession number if available, and source (including location for collected wild specimens).	Not Applicable	
**Microbes:** provide species and strain, unique accession number if available, and source.	Not Applicable	

Human research participants	Information included in the manuscript?	In which section is the information available? (Reagents and Tools Table, Materials and Methods, Figures, Data Availability Section)
If collected and within the bounds of privacy constraints report on age, sex and gender or ethnicity for all study participants.	Not Applicable	

Core facilities	Information included in the manuscript?	In which section is the information available? (Reagents and Tools Table, Materials and Methods, Figures, Data Availability Section)
If your work benefited from core facilities, was their service mentioned in the acknowledgments section?	Yes	Acknowledgements

Design

Study protocol	Information included in the manuscript?	In which section is the information available? (Reagents and Tools Table, Materials and Methods, Figures, Data Availability Section)
If study protocol has been **pre-registered, provide DOI in the manuscript**. For clinical trials, provide the trial registration number **OR** cite DOI.	Not Applicable	
Report the **clinical trial registration number** (at ClinicalTrials.gov or equivalent), where applicable.	Not Applicable	

Laboratory protocol	Information included in the manuscript?	In which section is the information available? (Reagents and Tools Table, Materials and Methods, Figures, Data Availability Section)
Provide DOI OR other citation details if **external detailed step-by-step protocols** are available.	Not Applicable	

Experimental study design and statistics	Information included in the manuscript?	In which section is the information available? (Reagents and Tools Table, Materials and Methods, Figures, Data Availability Section)
Include a statement about **sample size** estimate even if no statistical methods were used.	Not Applicable	
Were any steps taken to minimize the effects of subjective bias when allocating animals/samples to treatment (e.g. **randomization procedure**)? If yes, have they been described?	Not Applicable	
Include a statement about **blinding** even if no blinding was done.	Not Applicable	
Describe **inclusion/exclusion criteria** if samples or animals were excluded from the analysis. Were the criteria pre-established? If sample or data points were omitted from analysis, report if this was due to attrition or intentional exclusion and provide justification.	Not Applicable	
For every figure, are **statistical tests** justified as appropriate? Do the data meet the assumptions of the tests (e.g., normal distribution)? Describe any methods used to assess it. Is there an estimate of variation within each group of data? Is the variance similar between the groups that are being statistically compared?	Yes	Materials and Methods

Sample definition and in-laboratory replication	Information included in the manuscript?	In which section is the information available? (Reagents and Tools Table, Materials and Methods, Figures, Data Availability Section)
In the figure legends: state number of times the experiment was **replicated** in laboratory.	Yes	Figure legends
In the figure legends: define whether data describe **technical or biological replicates**.	Yes	Figure legends

Ethics

Ethics	Information included in the manuscript?	In which section is the information available? (Reagents and Tools Table, Materials and Methods, Figures, Data Availability Section)
Studies involving **human participants**: State details of **authority granting ethics approval** (IRB or equivalent committee(s), provide reference number for approval.	Not Applicable	
Studies involving **human participants**: Include a statement confirming that **informed consent** was obtained from all subjects and that the experiments conformed to the principles set out in the WMA Declaration of Helsinki and the Department of Health and Human Services Belmont Report.	Not Applicable	
Studies involving **human participants:** For publication of **patient photos**, include a statement confirming that consent to publish was obtained.	Not Applicable	
Studies involving experimental **animals**: State details of **authority granting ethics approval** (IRB or equivalent committee(s), provide reference number for approval. Include a statement of compliance with ethical regulations.	Yes	Materials and Mehods
Studies involving **specimen and field samples:** State if relevant **permits** obtained, provide details of authority approving study; if none were required, explain why.	Not Applicable	

Dual Use Research of Concern (DURC)	Information included in the manuscript?	In which section is the information available? (Reagents and Tools Table, Materials and Methods, Figures, Data Availability Section)
Could your study fall under dual use research restrictions? Please check biosecurity documents and list of **select agents and toxins** (CDC): https://www.selectagents.gov/sat/list.htm	Not Applicable	
If you used a select agent, is the security level of the lab appropriate and reported in the manuscript?	Not Applicable	
If a study is subject to dual use research of concern regulations, is the name of the **authority granting approval and reference number** for the regulatory approval provided in the manuscript?	Not Applicable	

Reporting

The MDAR framework recommends adoption of discipline-specific guidelines, established and endorsed through community initiatives. Journals have their own policy about requiring specific guidelines and recommendations to complement MDAR.

Adherence to community standards	Information included in the manuscript?	In which section is the information available? (Reagents and Tools Table, Materials and Methods, Figures, Data Availability Section)
State if relevant guidelines or checklists (e.g., **ICMJE, MIBBI, ARRIVE, PRISMA**) have been followed or provided.	Not Applicable	
For **tumor marker prognostic studies,** we recommend that you follow the **REMARK** reporting guidelines (see link list at top right). See author guidelines, under 'Reporting Guidelines'. Please confirm you have followed these guidelines.	Not Applicable	
For **phase II and III randomized controlled trials**, please refer to the **CONSORT** flow diagram (see link list at top right) and submit the CONSORT checklist (see link list at top right) with your submission. See author guidelines, under 'Reporting Guidelines'. Please confirm you have submitted this list.	Not Applicable	

Data Availability

Data availability	Information included in the manuscript?	In which section is the information available? (Reagents and Tools Table, Materials and Methods, Figures, Data Availability Section)
Have **primary datasets** been deposited according to the journal's guidelines (see 'Data Deposition' section) and the respective accession numbers provided in the Data Availability Section?	Yes	Materials and Methods
Were **human clinical and genomic datasets** deposited in a public access-controlled repository in accordance to ethical obligations to the patients and to the applicable consent agreement?	Not Applicable	
Are **computational models** that are central and integral to a study available without restrictions in a machine-readable form? Were the relevant accession numbers or links provided?	Not Applicable	
If publicly available data were reused, provide the respective **data citations in the reference list**.	Yes	Results, Materials and Methods, and Reference list
